# Tooth morphology elucidates shark evolution across the end-Cretaceous mass extinction

**DOI:** 10.1371/journal.pbio.3001108

**Published:** 2021-08-10

**Authors:** Mohamad Bazzi, Nicolás E. Campione, Per E. Ahlberg, Henning Blom, Benjamin P. Kear

**Affiliations:** 1 Subdepartment of Evolution and Development, Department of Organismal Biology, Uppsala University, Uppsala, Sweden; 2 Palaeoscience Research Centre, School of Environmental and Rural Science, University of New England, Armidale, New South Wales, Australia; 3 Museum of Evolution, Uppsala University, Uppsala, Sweden; Universidade de São Paulo, BRAZIL

## Abstract

Sharks (Selachimorpha) are iconic marine predators that have survived multiple mass extinctions over geologic time. Their prolific fossil record is represented mainly by isolated shed teeth, which provide the basis for reconstructing deep time diversity changes affecting different selachimorph clades. By contrast, corresponding shifts in shark ecology, as measured through morphological disparity, have received comparatively limited analytical attention. Here, we use a geometric morphometric approach to comprehensively examine tooth morphologies in multiple shark lineages traversing the catastrophic end-Cretaceous mass extinction—this event terminated the Mesozoic Era 66 million years ago. Our results show that selachimorphs maintained virtually static levels of dental disparity in most of their constituent clades across the Cretaceous–Paleogene interval. Nevertheless, selective extinctions did impact apex predator species characterized by triangular blade-like teeth. This is particularly evident among lamniforms, which included the dominant Cretaceous anacoracids. Conversely, other groups, such as carcharhiniforms and orectolobiforms, experienced disparity modifications, while heterodontiforms, hexanchiforms, squaliforms, squatiniforms, and †synechodontiforms were not overtly affected. Finally, while some lamniform lineages disappeared, others underwent postextinction disparity increases, especially odontaspidids, which are typified by narrow-cusped teeth adapted for feeding on fishes. Notably, this increase coincides with the early Paleogene radiation of teleosts as a possible prey source, and the geographic relocation of disparity sampling “hotspots,” perhaps indicating a regionally disjunct extinction recovery. Ultimately, our study reveals a complex morphological response to the end-Cretaceous mass extinction and highlights an event that influenced the evolution of modern sharks.

## Abstract 2

Analysis of the tooth morphology of sharks across the end-Cretaceous mass extinction, 66 million years ago, shows that while generally unaffected, some apex predator shark lineages were selectively impacted; changing habitats and the differential survival of ‘fish-eating’ sharks also reveals responses to ecological cataclysm.

## Introduction

Fossils provide the only direct evidence of interplay between organisms and their environments over vast evolutionary timescales [1–3]. They are, therefore, crucial for exploring the drivers of past biodiversity change and can shed light on the origins of modern ecosystems [3]. However, the analytical challenge is to discern a genuine biological signal from the combined obfuscations of geologic, taphonomic, sampling, taxonomic, analytical, and interpretive biases [4–7]. While these may be impossible to overcome in entirety, the fossil records of some widely distributed and chronostratigraphically extended clades provide exceptional opportunities to characterize macroevolutionary processes through deep time.

Sharks constitute one such group because their dental remains are abundant in Mesozoic and Cenozoic marine deposits—a timeframe covering approximately 250 million years (Ma) [8,9]. Extant shark species are also ecologically disparate, encompassing a spectrum of macrophagous to microphagous predators that account for nearly half (42%) of all the currently documented chondrichthyan biodiversity (*N =* 1,193 species) [10,11]. Nevertheless, the various biological and environmental factors that have shaped shark evolution remain obscure. In particular, their capacity to survive mass extinctions is relevant for understanding the dramatic decline of shark populations observed in our modern oceans [11–16].

The end-Cretaceous mass extinction (approximately 66 Ma), which marks the Cretaceous/Paleogene (K/Pg) chronostratigraphic boundary, is especially pertinent because it profoundly disrupted marine ecosystems but has disputed implications for shark species diversity and morphological disparity. Indeed, contrasting interpretations advocate either limited [17] or complex interrelationships of biotic and abiotic drivers that seemingly influenced shark evolution from before, during, and after the K/Pg mass extinction event [18–21]. Here, we explore these contentions via a comprehensive assessment of shark dental morphology across the end-Cretaceous mass extinction. Our study expands on previous studies that targeted either geographically localized [22,23] or clade-specific [21] assemblages. We use a dataset of 1,239 fossil shark teeth, representing 9 major selachimorph clades sampled at global and regional scales. These groups include the following: the Galeomorphii orders Carcharhiniformes, Heterodontiformes, Lamniformes, Orectolobiformes; the Squalomorphii orders Echinorhiniformes, Hexanchiformes, Squaliformes, Squatiniformes; and the extinct [†] Synechodontiformes. Our approach uses geometric morphometrics to compare disparity and morphospace patterns across a constrained 27.6-million-year interval spanning the Campanian and Maastrichtian ages of the Late Cretaceous (83.6 to 66 Ma) to the Danian, Selandian, and Thanetian ages (= Paleocene epoch) of the early Paleogene (66 to 56 Ma). We test the following hypotheses that: (1) selachimorph disparity was in decline before marine ecosystem disruption during the end-Cretaceous mass extinction, resulting from major marine transgressions during the Maastrichtian [23,24]; (2) selachimorph taxonomic richness depletion at the K/Pg boundary [18,19] was coupled with eco-morphological turnover; (3) apex predator shark lineages were disproportionately impacted, consistent with similar losses in marine tetrapods and osteichthyans [18,21,25]; and (4) extinction and recovery patterns were consistent at global and regional scales [21–23].

## Materials and methods

### Dataset assembly

Our dataset was compiled using photographs and graphic images derived from first-hand observations or the literature (S1 Data, Fig A and Tables A and B in S1 Text). Following recommended best practices [21,26,27], we screened the raw image data to include only those depicting complete tooth crowns with adequate resolution to determine the crown–root junction. The global sampling includes nearly all major selachimorph orders (Fig A in S1 Text, Fig B in S1 Text, panel A), except for Pristiophoriformes (Sawsharks), which are sparsely documented [8,28]. We also elevated Echinorhinidae to Echinorhiniformes [29–31] and employed a sensitivity analysis to test the morphospace occupation and disparity effects of †Synechodontiformes, which has been classified as either a clade within Galeomorphii or a neoselachian sister lineage (Fig B in S1 Text, panel A) [32–35]. Finally, most images were captured in the labial aspect, unless only a lingual view was available, and with the tooth apex directed to the left (Fig C in S1 Text). The equivalency of labial and lingual views [21] was tested using ordinary least-squares linear models based on a subsample of the labial and lingual view data (*N =* 866).

Time-binning encompassed 5 geochronological ages spanning the immediate K/Pg interval: Campanian and Maastrichtian/Danian, Selandian, and Thanetian. However, we also implemented an alternative 4-age time-binning scheme that pooled temporally ambiguous specimens assigned to the Danian and Selandian (Tables C and D in S1 Text). In addition, we carried out analyses using a subsample of the dataset (*N =* 659) for which “early” and “late” sub-ages could be defined for the Campanian and Maastrichtian and “early,” “middle,” and “late” for the Danian (Table E in S1 Text). Numerical age values, in Ma, were taken from the International Chronostratigraphic Chart v2020/03 [36] and used as references for plotting.

### Acquisition of geometric shape data

Landmark-based geometric morphometrics quantifies biological shapes as a series of evolutionary homologous points in Cartesian space [26,27,37–42]. However, evolutionary homology [37,38] between landmarks cannot be assumed because of the inherent morphological variability in shark teeth (e.g., the location and number of cusplets). As a result, we designated our landmark placements based on topological rather than evolutionary homology [41].

Landmark digitization was carried out in *tpsDig2* v. 2.31 [43] with resampling to a standard number of equidistant semilandmarks using customized code in *R* v. 4.0.5 [44]. The resulting scheme comprised 2 open curves defined by semilandmarks but anchored by 3 fixed landmarks: Type 1 landmarks delimited the mesial and distal crown–root junctions, and a Type 2 landmark pinpointed the tooth apex (sensu [37]; Fig B in S1 Text, panel B, Table F in S1 Text). The number of semilandmarks (k) was determined using resampling of the mesial and distal curves at equal spacings of k = 40, 60, 80, 100, 120, 140, and 160. Qualitative observations (Figs D and E in S1 Text) found that k = 160 best-captured tooth shape complexity and included distal and mesial curves of 78 and 79 sliding semilandmarks, respectively. Tooth serrations were not digitized because of limited image resolution, but we acknowledge that these structures are functionally important [45].

Lastly, to screen for possible digitization errors, we extracted a random subsample of 30 tooth images (Table G in S1 Text) and used a one-way ANOVA to calculate the intraclass correlation coefficient (*R*) [26,46,47] (also see S1 Text). We also ran a 2-block partial least-squares (2B-PLS) analysis to infer covariation between the landmark datasets. Other exploratory procedures were employed to assess measurement error: (1) a manual survey of digitized images to confirm landmark placement accuracy; (2) screening of outliers visualized in the morphospace plots and associated thin plate spline (TPS) deformation grids, as well as being identified using the *plotOutliers* search function in *geomorph* v. 4.0.0 [48].

### Morphometric analysis

To standardize our digitized specimens for unit size, position, and rotation, we used a generalized Procrustes analysis (GPA) [49,50] that minimizes the bending energies to optimize the positions of the sliding semilandmarks [50,51]. Because large numbers of semilandmarks can impinge on GPA convergence [26,38], we varied the iteration frequency by arbitrarily increasing the *max*.*iter* argument in *gpagan* to compare convergence criteria Q-values (= Procrustes sum of squares). The resulting consensus shape configurations were then inspected (Fig F and Table H in S1 Text).

The aligned Procrustes coordinates were ordinated via a principal components analysis (PCA) based on the singular value decomposition of the variance–covariance matrix. Shape variation was depicted as both TPS deformation grids and deformation isolines to generate a concentration “heat map” [52]. Morphospace was visualized using back-transformation [53,54]. All analyses were carried out in *R* v. 4.0.5 [44] with the *geomorph* v. 4.0.0 [48] and *RRPP* v. 1.0.0 [55] packages; visualization used the *ggplot2* package [56]. All data and *R* scripts are available from *Data Dryad* under DOI: https://doi.org/10.5061/dryad.c866t1g5n [57].

### Temporal analyses of morphospace

Morphospace was depicted as time-bin box plots that incorporate the arithmetic mean, median, and modal values. Confidence intervals were calculated using nonparametric bootstrapping with 1,000 resamples. Comparisons between multiple central tendency values accommodate for differences in frequency distributions. Modal shape configurations correspond to the region of maximum frequency calculated as:

$${Mo}_{{shape}_{j}}={\hat{\mu }}_{p}+{Mo}_{C_{j}}\times \Gamma_{j})$$ (1)

where ${Mo}_{{shape}_{j}}$ is the modal shape configuration of the *j*th PC; ${\hat{\mu }}_{p}$ represents the mean shape configuration of the whole sample; ${Mo}_{C_{j}}$ is the mode of the *j*th PC axis; and, Γ_*j*_ is the rotation matrix corresponding to the *j*th PC. ${Mo}_{{shape}_{j}},\;{\hat{\mu }}_{p}$ and Γ_*j*_ take the form of k × m matrices and were plotted as TPS deformation grids; “k” is the number of landmarks and “m” is the dimensions, in our case two.

Multivariate normality was assessed using a Henze–Zirkler test [58] (*HZ* = 4,956, *p* = 0) (Fig G in S1 Text). Statistical comparisons between time-bins used a nonparametric Procrustes analysis of variance implemented in the *RRPP* package [55].

### Temporal analyses of disparity

We calculated Procrustes variance (PV) as a measure of disparity [41,59] based on the 2D (k × m × N) Procrustes-aligned landmark dataset:

$${SSW}_{n}={\left(x_{1}-\bar{x_{1}}\right)}^{2}+{\left(y_{1}-\bar{y_{1}}\right)}^{2}+\cdots +{\left(x_{k}-\bar{x_{k}}\right)}^{2}+{\left(y_{k}-\bar{y_{k}}\right)}^{2}$$ (2)

where *SSW*_*n*_ is the sum of the square distances between the coordinates (*x*_*k*_ and *y*_*k*_) of observation *n* and their associated mean ($\bar{x_{k}}$ and $\bar{y_{k}}$) [48]. This equation can be alternatively depicted as:

$$SSW_{n}=\sum_{k=1}^{k}{\left(x_{k}-{\bar{x}}_{k}\right)}^{2}+{\left(y_{k}-\bar{y_{k}}\right)}^{2}$$ (3)

All *SSW*_*n*_ values in a given time-bin *t* are then summed and divided by the sample size at that time (*N*_*t*_) to measure PV across all observations [48]. Note that the following equation was erroneously presented in Bazzi and colleagues [21].


$${PV}_{t}=\frac{\sum_{n=1}^{n}{SSW}_{n}}{N_{t}}$$ (4)

Disparity within each time-bin was partitioned according to their taxonomic order-level classifications, which determined the clade-specific contributions to the overall disparity. This calculation equates to (3), but with distances measured relative to the mean of the group *i* ($\bar{x_{k|i}}$), rather than the overall mean [48]:

$$SSW_{n}=\sum_{k=1}^{k}{\left(x_{k}-\bar{x_{k|l}}\right)}^{2}+{\left(y_{k}-\bar{y_{k|l}}\right)}^{2}$$ (5)

The partial *PV* for the group *i* in a specific time-bin *t* is ∴

$${PV}_{i|t}=\frac{\sum_{n=1}^{n}{SSW}_{n}}{n_{i}}\times \frac{n_{i}}{N-1}$$ (6)

where *n*_*i*_ is the sample size of group *i* and *N* is the total sample size within *t* [48].

Computationally, these equations are solved in *geomorph* [48], with the expectation that additive partial disparities [60] for sampling within each time-bin approximate the total *PV* given *t*.

We used a residual randomization permutation procedure (RRPP) with 1,000 permutations [55] to test null hypotheses for our multivariate shape data as:

$$\begin{array}{l} H_{0}:|{PV}_{t1}-{PV}_{t2}|=0\\ H_{A}:|{PV}_{t1}-{PV}_{t2}|>0 \end{array}$$ (7)

*H*_*0*_ assumes that pairwise absolute differences between PVs across 2 given time-bins (e.g., *t1* and *t2*) will be zero. *H*_*A*_ alternatively stipulates that the difference will be greater than zero.

We also applied nonparametric bootstrap resampling to estimate confidence intervals around disparity. All post hoc pairwise comparisons of group means were subject to false discovery rate (FDR) adjustments of *p*-values to mitigate the increased risk of Type I errors associated with multiple comparisons [61].

### Geographic distribution in the fossil record

We accommodated for sample size biases inherent in the fossil record [4,62–64] via rarefaction comparisons of time-scaled PV [21]. These involved subsampling (999 iterations) of all time-bins to a minimum size (Tables A, C, and D in S1 Text), after which 95% prediction intervals were calculated. Geographic subsampling focused on the UNESCO World Heritage fossil locality at Stevns Klint in Denmark (Fig H in S1 Text), which preserves exceptionally rich selachimorph assemblages [65,66] spanning the K/Pg succession [67]. However, we also calculated partial disparities for each time-bin based on marine depositional basins, designated *i* in (6). Although the precise boundaries of these basins are ambiguous, they do provide a convenient proxy for comparing regional versus global disparity signals across a broader subsampled series.

### Influences of heterodonty

Sharks are known to exhibit both monognathic (variation along the tooth row) and dignathic (variation between the upper and lower jaws) heterodonty [8,68], although this can be difficult to discriminate from isolated fossil teeth [69]. To mitigate, we relied upon established diagnostic criteria [21,70] to categorize our specimens as representing parasymphyseal, anterior, lateroposterior, or posterior tooth positions (N_MH_ = 897), and deriving from either the upper or lower tooth rows (N_DH_ = 334) (see S1 Text)

Shape∼ToothPosition+t (8)

where the aligned Procrustes coordinates (*shape*) are described as a function of monognathic or dignathic *Tooth Position* at age *t*.

Because developmental [70,71] and ontogenetic factors [70,72–74] also affect selachimorph tooth morphology, our analyses are presented with the caveat that adequate intraspecific coverage was assumed for each order-level clade.

## Results

### Digitization measurement error

Visual comparison of the computed consensus (mean) tooth shapes (Fig I in S1 Text) indicates consistent digitization across landmark datasets. Accordingly, an intraclass correlation coefficient (*R*) of 2% (or 1 in 50) was calculated based on the aligned Procrustes coordinates and their error replicate counterparts (*N =* 30) (Table I in S1 Text). Pearson product–moment correlation (t = 1,874.7, *df* = 9598, *p*-value << 0.001, *R* = 0.99) and a 2B-PLS test (r-pls = 0.997, *p*-value = 0.001, Z = 7.136) unambiguously demonstrate dataset compatibility (Fig J in S1 Text).

### PCA visualization

**Fig 1:**
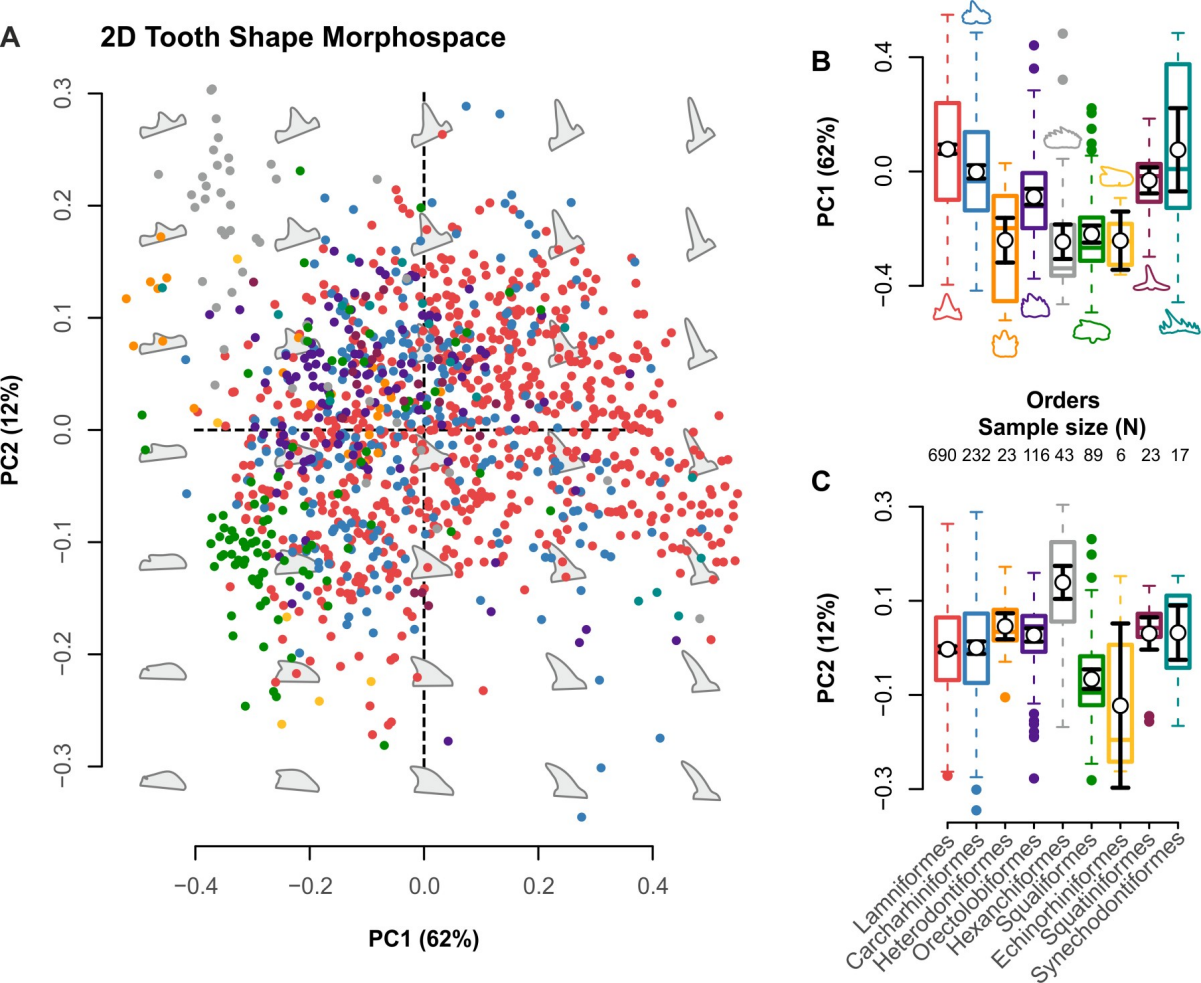
Morphospace distribution. (A) Multivariate shape space for the *N =* 1,239 global selachimorph tooth sample. Theoretical backtransform tooth shapes (gray) indicate shape variability across morphospace as defined by PC1/PC2. (B, C) Box-and-whisker plots indicating average morphospace occupation along (B) PC1 and (C) PC2. Error bars represent 95% confidence intervals. Proportion of variance and group sample sizes are listed in the axis labels. The data used in this analysis can be accessed online at https://doi.org/10.5061/dryad.c866t1g5n.

PC1 to PC4 explain 89.28% of the shape variance (Fig 1A–1C, Fig K in S1 Text), with the remaining PC axes collectively describing 10.71% (Fig K and Table J in S1 Text). PC1 (62%) captures tooth height and width variation from apicobasally tall and narrow teeth to mesiodistally broad and low crowns (Fig L in S1 Text, panel A). PC2 (12%) alternatively represents distally recurved teeth with low “heels” versus upright triangular teeth with lateral cusplets (Fig L in S1 Text, panel B). PC3 (11%) tracks tall and conical to distally wide and recurved teeth with pronounced lateral cusplets (Fig L in S1 Text, panel C). PC4 (5%) depicts a spectrum of short triangular teeth with reduced cusplets to tall crowns with prominent cusplets commensurate in height with the main cusp (Fig L in S1 Text, panel D).

While there is a substantial overlap in tooth morphologies between selachimorph clades (Fig 1A–1C), a general Procrustes one-way ANOVA found significant differences between the group means, both generally and along specific axes of variance (Table 1). Such results are supported by visible segregation between the clades in morphospace (Fig 1A–1C) and are further illustrated by measures of central tendency, distribution symmetries, and normality and multimodality tests (Table K in S1 Text).

**Table 1:** Nonparametric analysis of variance based on RRPP for *N =* 1,239. Coefficient estimation via OLS. Type I (sequential) sums of squares were used to calculate the sums of squares and cross-products matrices. Effect sizes (Z) are based on the F distribution.

	*d*.*f*.	SS	MS	R^2^	F	Z	Pr(>F)
**All PC axes**							
~ groups (clades)	8	18.744	2.34294	0.18931	35.904	10.892	0.001**
**PC1**							
~ groups (clades)	8	13.737	1.71707	0.22474	44.569	7.3642	0.001**
**PC2**							
~ groups (clades)	8	1.4895	0.186190	0.12352	21.668	5.8106	0.001**
**PC3**							
~ groups (clades)	8	2.1710	0.271372	0.20412	39.431	7.0403	0.001**
**PC4**							
~ groups (clades)	8	0.3986	0.049819	0.08708	14.665	5.1632	0.001**

*d*.*f*., degrees of freedom; F statistics, F value by permutation; MS, mean squares; OLS, ordinary least squares; RRPP, residual randomization permutation procedure; R^2^, coefficient of determination; SS, sequential sums of squares; Z, effect size.

*p*-Values are based on 999 permutations.

### Global and regional disparity

**Fig 2:**
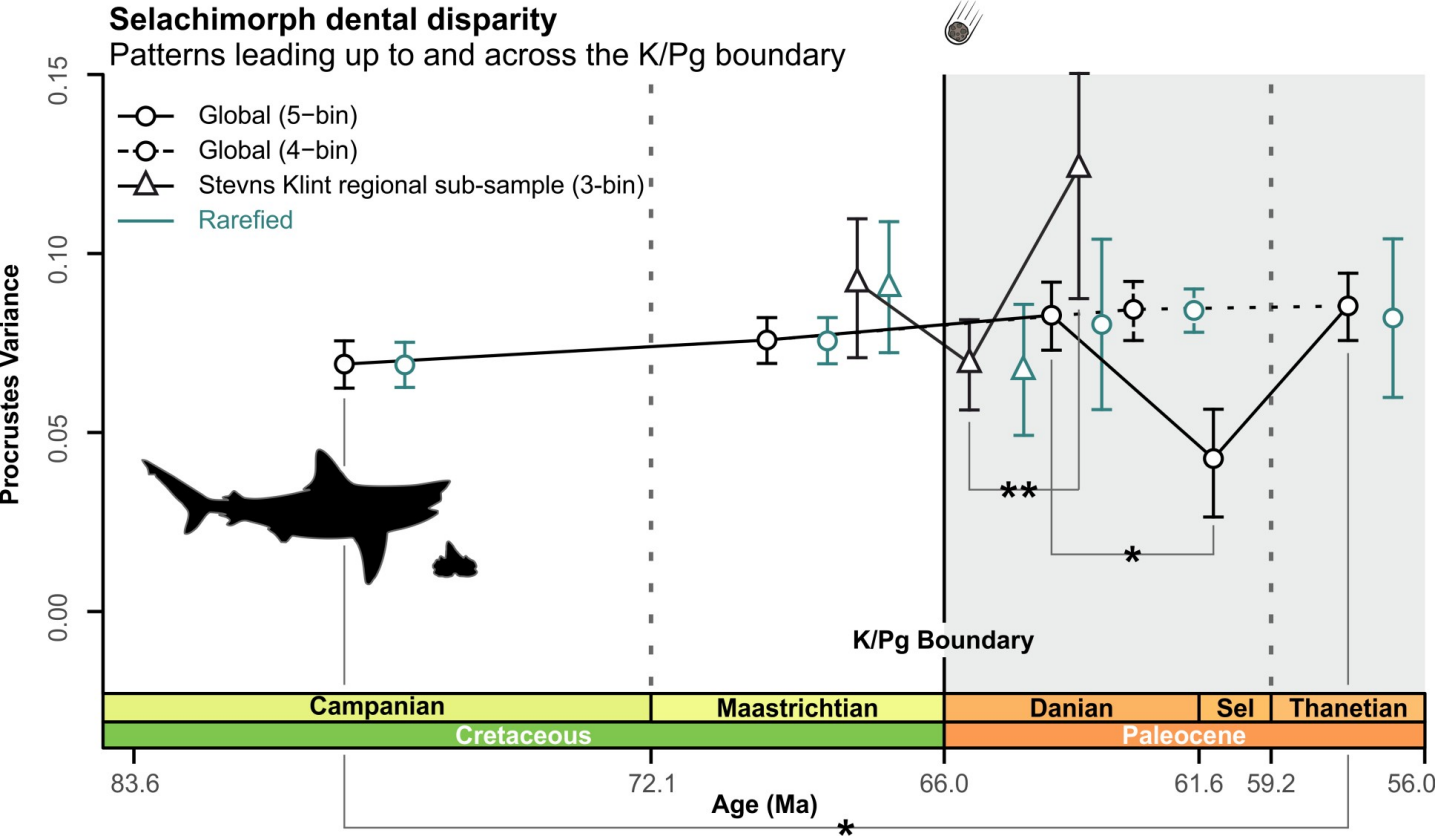
Global and regional level disparity of Selachimorpha. Dental disparity with bootstrapped prediction intervals for the 5- and 4-age time-binning schemes. The 5-age time-binning scheme (*N =* 1,156) utilized sample rarefaction on the lowest sampled Selandian time-bin (*N* = 28). The 4-age time-binning scheme (*N* = 1 198) used the Thanetian (*N* = 187). The Stevns Klint regional subsample (3-age time-bin) used the middle Danian (*N* = 25). Significant FDR-adjusted *p*-values for multiple comparisons are indicated (*p < 0.05, **p < 0.005). Silhouette graphics created by MB. The data used in this analysis can be accessed online at https://doi.org/10.5061/dryad.c866t1g5n. FDR, false discovery rate; K/Pg, Cretaceous/Paleogene; Ma, million years; Sel, Selandian.

We find overall stability in selachimorph global disparity across the Campanian–Thanetian interval (Fig 2, Table L in S1 Text). The only exception was a significant decline within the Selandian time-bin (PV_Danian_ = 0.082; PV_Selandian_ = 0.042; *p* = 0.033), which may be a product of sampling (*N =* 28) and/or uneven clade representation. Disparity during the Thanetian exceeded (Fig 2, Fig M and Table L in S1 Text) that of the preextinction Campanian (PV_Campanian_ = 0.069; PV_Thanetian_ = 0.085; *p* = 0.033) and was unaffected by pruning of †Synechodontiformes as a possible selachimorph stem group (Fig 2, Table M in S1 Text). Comparisons with the Stevns Klint regional subsample found no significant change in tooth disparity (PV_lateMaastrichtian_ = 0.091; PV_earlyDanian_ = 0.069; *p* = 0.106) across the K/Pg boundary (Fig 2, Table N in S1 Text). However, a significant disparity increase occurred after the extinction event from the early to middle Danian (PV_earlyDanian_ = 0.069; PV_middleDanian_ = 0.123; *p* = 0.005).

### Superorder-level clade disparity

**Fig 3:**
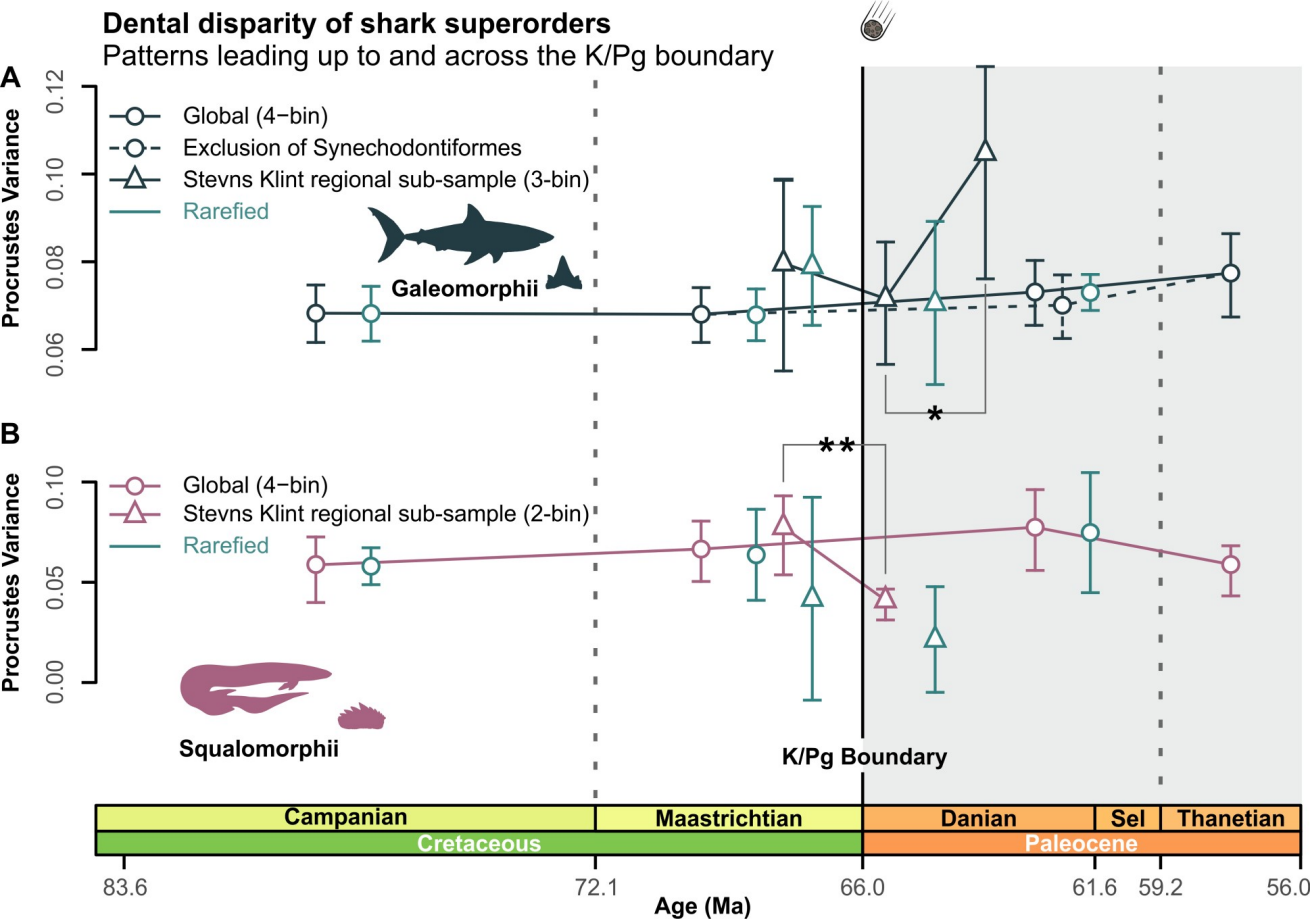
Superorder-level clade disparity profiles. (A, B) Global/regional raw and rarefied dental disparity trajectories for Galeomorphii (including and excluding †Synechodontiformes) and Squalomorphii. Sample rarefaction: Galeomorphii, *N =* 169; Squalimorphii, *N* = 18. Significant FDR-adjusted *p*-values for multiple comparisons are indicated (*p < 0.05, **p < 0.005). Silhouette graphics created by MB. The data used in this analysis can be accessed online at https://doi.org/10.5061/dryad.c866t1g5n. FDR, false discovery rate; K/Pg, Cretaceous/Paleogene; Ma, million years; Sel, Selandian.

Relative stasis characterized the disparity of both galeomorphs and squalomorphs across the Maastrichtian–Danian-Selandian (Fig 3A and 3B, Tables O, P, and Q in S1 Text). This result is consistent even after the exclusion of †synechodontiforms (Fig 3A, Table P in S1 Text). Conversely, independent testing of the Stevns Klint regional subsample produces a significant disparity increase among galeomorphs (PV_earlyDanian_ = 0.069; PV_middleDanian_ = 0.109; *p* = 0.022) in the early to middle Danian (Fig 3A, Tables R and S in S1 Text) and a corresponding reduction in squalomorph disparity (Fig 3B, Table T in S1 Text) across the late Maastrichtian to early Danian (PV_lateMaastrichtian_ = 0.077; PV_earlyDanian_ = 0.041; *p* = 0.005).

### Order-level clade disparity

**Fig 4:**
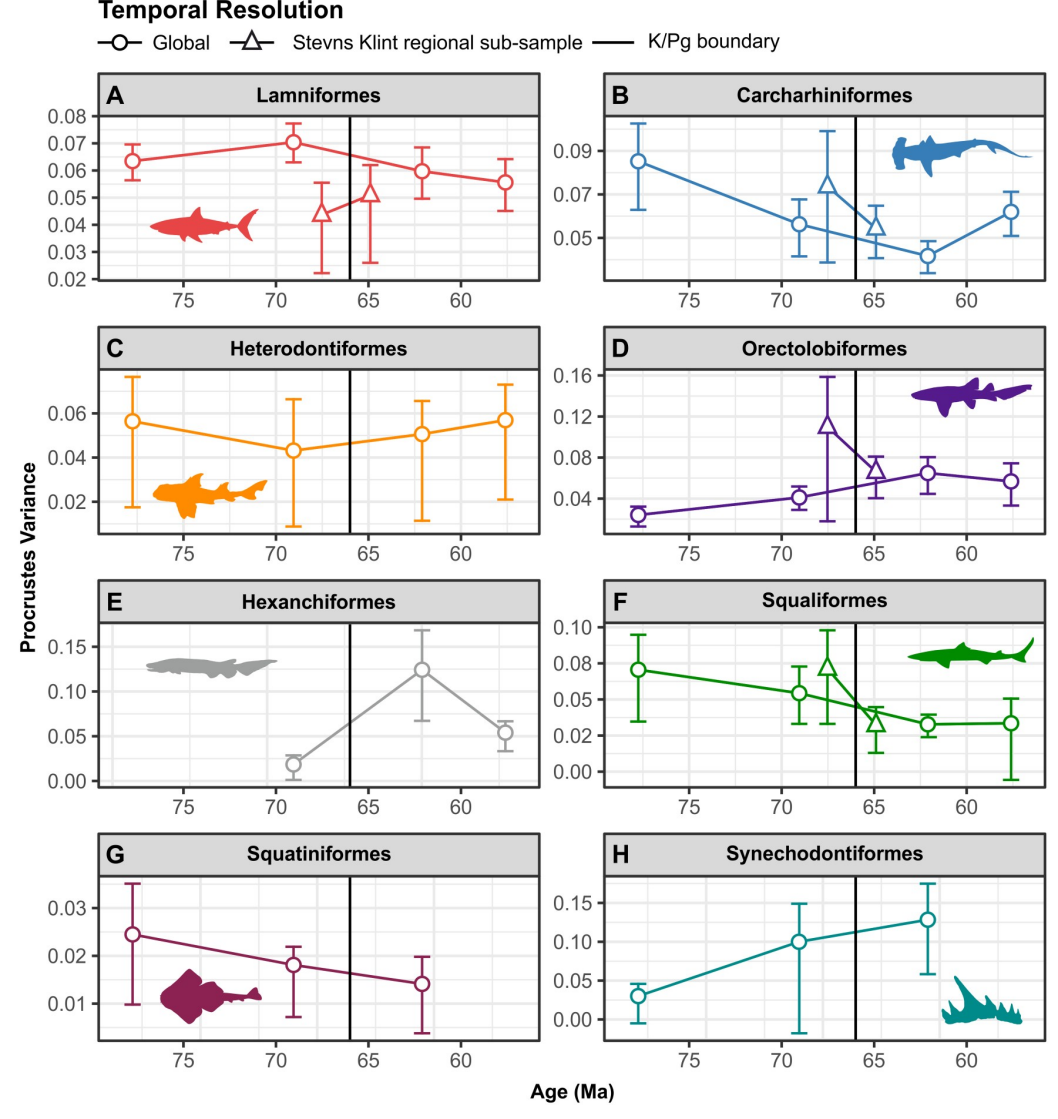
Global and regional clade-specific (order-level) disparity profiles. (A–D) Temporal resolution varies between groups but broadly tracks the time-binned K/Pg interval. Clade-level subsampling is shown in Tables C and D in S1 Text. Silhouette graphics created by MB and Julius Csotonyi (https://csotonyi.com/). The data used in this analysis can be accessed online at https://doi.org/10.5061/dryad.c866t1g5n. K/Pg, Cretaceous/Paleogene; Ma, million years.

Lamniform and carcharhiniform (Fig 4A and 4B, Tables U and V in S1 Text) disparities were highest during the Campanian and Maastrichtian interval (Fig 4A and 4B). Despite these high levels, global lamniform disparity was demonstrably stable across the Maastrichtian–Danian-Selandian time-bins (PV_Maastrichtian_ = 0.070; PV_Danian-Selandian_ = 0.059; *p* = 0.396; Fig 4A) and showed no significant change by the Thanetian (PV_Maastrichtian_ = 0.070; PV_Thanetian_ = 0.055; *p* = 0.264; Fig 4A).

Carcharhiniforms otherwise exhibited a marked disparity decline from the Campanian to Danian-Selandian when using the global sample, but not in the Stevns Klint regional subsample (Fig 4B, Table V in S1 Text). A distinct disparity drop across the Campanian–Maastrichtian (PV_Campanian_ = 0.085; PV_Maastrichtian_ = 0.056; *p* = 0.024) was followed by stasis from the Maastrichtian–Danian-Selandian (PV_Maastrichtian_ = 0.056; PV_Danian-Selandian_ = 0.041; *p* = 0.125) and a subsequent disparity increase by the Thanetian (PV_Danian-Selandian_ = 0.041; PV_Thanetian_ = 0.061; *p* = 0.020) (Fig 4B).

Heterodontiform disparity was uniform across the Campanian–Thanetian interval (Fig 4C, Table W in S1 Text). On the other hand, orectolobiforms showed a significant disparity increase (PV_Campanian_ = 0.024; PV_Danian-Selandian_ = 0.064; *p* = 0.016) between the Campanian and Danian-Selandian (Fig 4D, Table X in S1 Text). We recover no significant change in orectolobiform disparity for any time-bin comparisons, including the Maastrichtian–Danian-Selandian (PV_Maastrichtian_ = 0.041; PV_Danian-Selandian_ = 0.064; *p* = 0.096), which is consistent with the results based on the Stevns Klint regional subsample (PV_lateMaastrichtian_ = 0.109; PV_earlyDanian_ = 0.066; *p* = 0.294) (Fig 4D).

Hexanchiform disparity was not statistically differentiated across the Maastrichtian–Danian-Selandian (PV_Maastrichtian_ = 0.018; PV_Danian-Selandian_ = 0.124; *p* = 0.104) nor between the Danian-Selandian and Thanetian (PV_Danian-Selandian_ = 0.124; PV_Thanetian_ = 0.054; *p* = 0.072) (Fig 4E, Table Y in S1 Text). Squaliforms similarly maintained stable disparity from the Maastrichtian–Danian-Selandian (PV_Maastrichtian_ = 0.054; PV_Danian-Selandian_ = 0.032; *p* = 0.084), both within the global and Stevns Klint regional samples (Fig 4F, Table Z in S1 Text). Finally, squatiniform and †synechodontiform disparities were also stable, but their small sample sizes make interpretations equivocal (Fig 4G and 4H, Tables AA and BB in S1 Text).

### Global partial disparity

**Fig 5:**
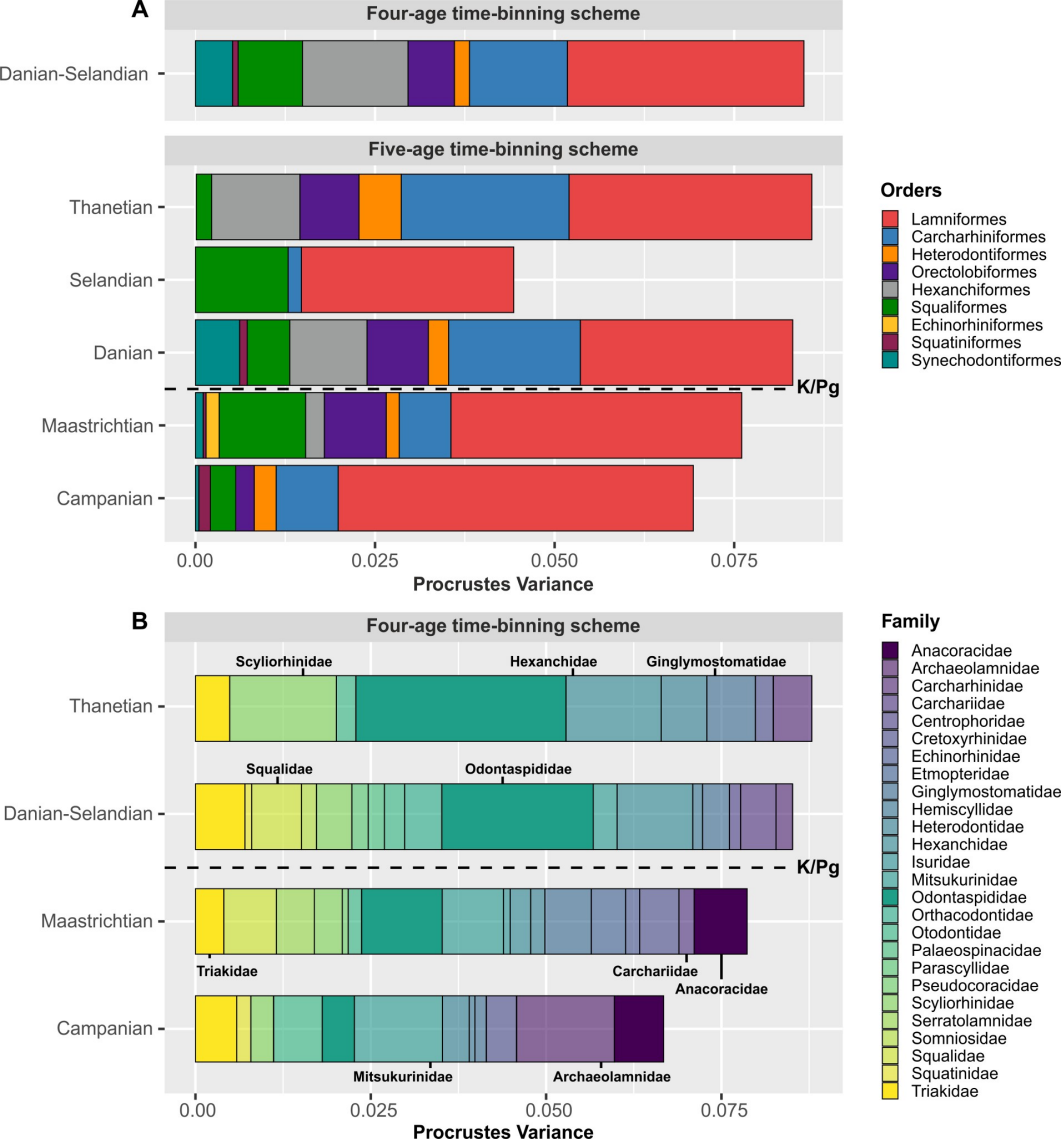
Order- and family-level partial disparity. (A, B) Grouped bar plot depicting clade-level contributions to overall disparity in each time-bin. (B) Families with sample sizes of *N* < 5 were omitted. Resulting data total = 26 families and 1,039 specimens with the global 4-age time-binning scheme. The data used in this analysis can be accessed online at https://doi.org/10.5061/dryad.c866t1g5n.

PVs indicate that selachimorph global disparity was driven by lamniforms during the Campanian and Maastrichtian (Fig 5A). Unsurprisingly, lamniforms were also the most numerically abundant taxa during this time (Fig A in S1 Text). Lamniform contribution to global disparity subsequently decreased in the Danian (Fig 5A) but increased again in the Selandian, although the sample size is small (Table C in S1 Text). Overall, lamniforms accounted for almost all of the global selachimorph variance calculated across the K/Pg time interval (Fig 5A). Carcharhiniforms and hexanchiforms otherwise contributed to a global disparity increase from the Danian to the Thanetian (Fig 5A).

Despite uneven sampling, family-level partial disparities (Fig 5B) reveal that archaeolamnids, mitsukurinids, and anacoracids provided the primary sources of selachimorph disparity during the Campanian. Odontaspidids sequentially increased their disparity from the Danian-Selandian to the Thanetian (Fig 5B). Correspondingly, triakids, hexanchids, and squalids contributed markedly to non-lamniform disparity in the Danian-Selandian, with scyliorhinids increasing disparity in the Thanetian. Other clades, such as ginglymostomatids and hexanchids, also increased disparity from the Danian-Selandian–Thanetian (Fig 5B).

### Geographic distribution of disparity

**Fig 6:**
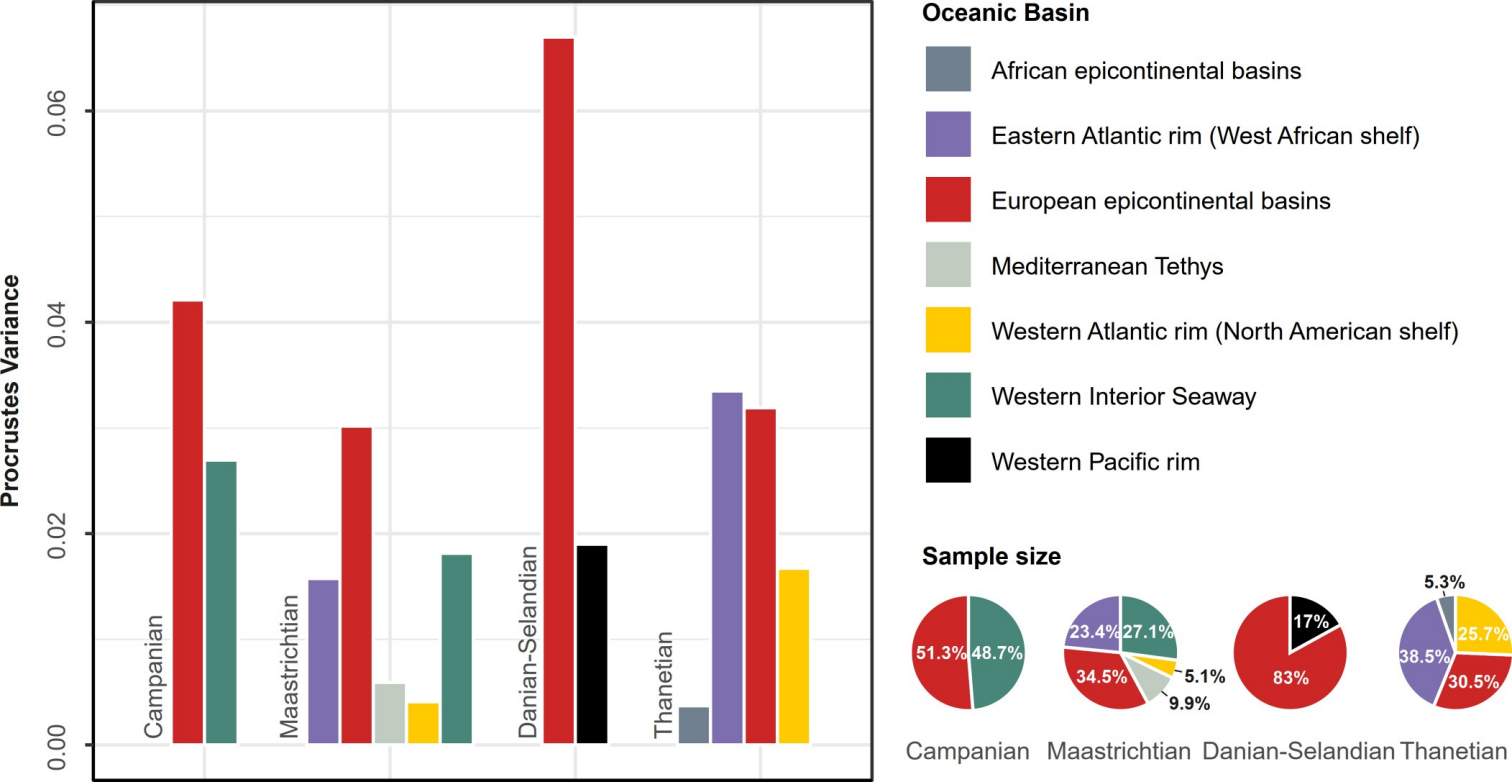
Geographic distribution of disparity. Mesozoic marine depositional basins are based on Wretman and colleagues [75]. Basins with sample sizes of *N* < 10 were omitted. Pie graphs depict sample proportions. The data used in this analysis can be accessed online at https://doi.org/10.5061/dryad.c866t1g5n.

Samples from the North American Western Interior (WIB) and European epicontinental (EEB) basins accounted for most of the disparity during the Campanian (Fig 6). Alternatively, the Maastrichtian incorporated a substantial contribution from the Ouled Abdoun and Tarfaya basins (= Eastern Atlantic rim [EAR]) of Morocco (Fig 6). In general, geographic regions with small representative subsamples yielded comparatively low disparities during the Maastrichtian, including the North African Mediterranean Tethys and North American Western Atlantic rim (WAR) (Fig 6). By contrast, intensive sampling of Stevns Klint in Denmark and the Limhamn Quarry in Sweden skewed the Danian-Selandian geographic distribution toward these regions and revealed the inordinate influence of lagerstätten deposits on our global disparity signal (Fig 6). Lastly, the Thanetian showed a return to more geographically widespread sampling across a continuous *trans*-Atlantic belt, spanning the African epicontinental basins and EAR to the EEB and WAR (Fig 6).

### Effects of heterodonty on disparity

Application of the monognathic heterodonty model (Tables CC and DD in S1 Text) produced a significant disparity increase across the Maastrichtian–Danian-Selandian (PV_Maastrichtian_ = 0.066; PV_Danian-Selandian_ = 0.082; *p* = 0.032). The combined monognathic and dignathic heterodonty model similarly indicates a disparity increase from the Campanian to Maastrichtian (*p* = 0.016). However, a dignathic heterodonty and multivariate monognathic*dignathic heterodonty interaction model yields no comparable disparity shifts (Fig N and Tables CC and DD in S1 Text), and additional model comparisons found consistent disparity across the Campanian–Thanetian interval, despite some inflation of absolute values (Fig N in S1 Text).

### Global morphospace

**Fig 7:**
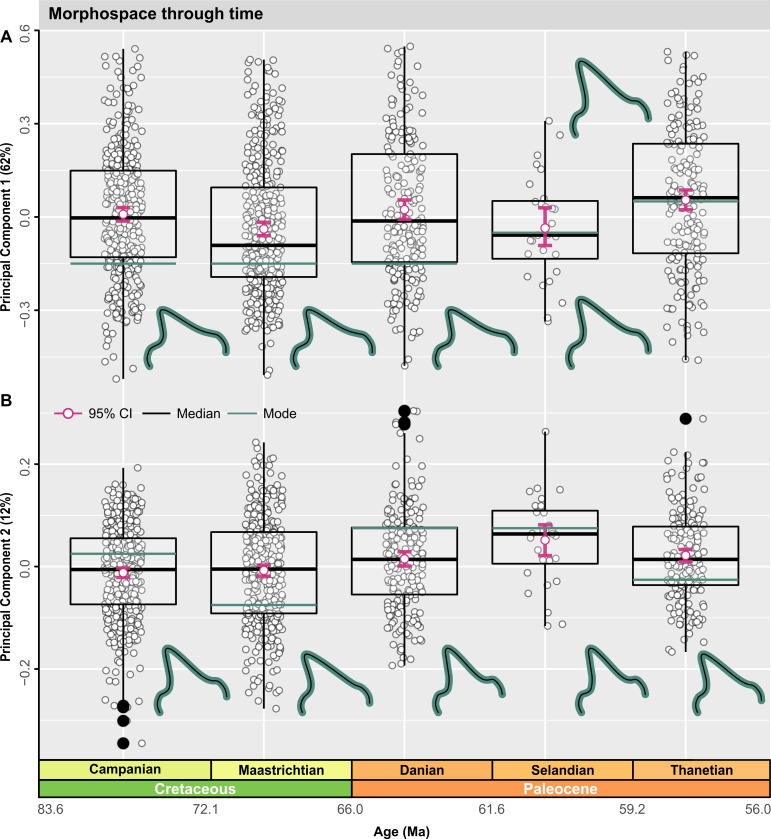
Global morphospace time series. (A, B) Jittered box plots visualizing the distribution of time-bins along PC1 and PC2. Graph depicts patterns of overall shape change using the 5-age time-binning scheme. Measures of central tendency, including the median (black line) and arithmetic mean (pink), are shown with computed 95% nonparametric bootstrap confidence intervals and potential outliers (closed black points). TPS grids indicate changes in modal value (green line) between time-bins. The data used in this analysis can be accessed online at https://doi.org/10.5061/dryad.c866t1g5n. Ma, million years; TPS, thin plate spline.

Pairwise comparisons along all PCs found significant differences (F_4, 1,155_ = 8.6269, *p* = 0.001) in morphospace distribution between time-bins (Fig 7, Table 2). The PC1 distributions are platykurtic (kurtosis < 3) and positively skewed, except during the Thanetian, which was negatively skewed (*g*1Thanetian = −0.0336). Distribution-specific interquartile ranges (IQRs) showed minimal morphospace dispersion along PC2 (Tables EE and FF in S1 Text). A Hartigan dip test for multimodality did not reject a unimodal distribution on PC1 or PC2; however, a significant positive morphospace shift did occur from the Maastrichtian–Danian on PC1 (Fig 7A, Fig O and Tables EE and FF in S1 Text). Notably, there was no corresponding change in modal shape configurations, although a reduction in positively loaded morphospace accompanied shortening of the minimum value ranges (Fig 7A, Fig O in S1 Text).

**Table 2:** Nonparametric analysis of variance based on RRPP for all PCs. Coefficient estimation via OLS. Type I (sequential) sums of squares were used to calculate sums of squares and cross-products matrices. Effect sizes (Z) based on F distribution.

	*d*.*f*.	SS	MS	R^2^	F	Z	Pr(>F)
**Selachimorpha**							
~ interaction (global 5-bin)	4	2.625	0.65621	0.02911	8.6269	5.0104	0.001**
~ interaction (global 4-bin)	3	2.219	0.73957	0.0234	9.5354	4.9451	0.001**
~ interaction (regional 3-bin)	2	0.840	0.41999	0.06043	4.8235	2.8145	0.002**
**Galeomorphii (Synechodontiformes included)**							
~ interaction (global 4-bin)	3	2.254	0.75136	0.02966	10.587	5.0617	0.001**
~ interaction (regional 3-bin)	2	0.7811	0.39053	0.07727	4.7316	2.796	0.003**
**Squalomorphii**							
~ interaction (global 4-bin)	3	0.9088	0.302947	0.07875	4.3028	3.8612	0.001**
~ interaction (regional 3-bin)	2	0.30381	0.151907	0.12846	2.5058	1.9687	0.015*

*d*.*f*., degrees of freedom; F statistics, F value by permutation; MS, mean squares; OLS, ordinary least squares; RRPP, residual randomization permutation procedure; R^2^, coefficient of determination; SS, sequential sums of squares; Z, effect size.

*p*-Values are based on 999 permutations.

The average value along PC1 shift positively across the Selandian–Thanetian (Fig 7A). Comparisons between the Campanian–Maastrichtian (*p* = 0.025) and Maastrichtian–Thanetian (*p* = 0.013) likewise yield significant morphospace shifts (Table GG in S1 Text).

The distributions for the Campanian and Danian on PC2 are leptokurtic (high kurtosis > 3), but low kurtosis prevails in the other time-bins (Fig 7B). A positive shift from the Maastrichtian (*g*1Maastrichtian = −0.0806) to Danian (*g*1_Danian_ = 0.2833) (Fig 7B, Table EE in S1 Text) coincided with a loss of negatively loaded tooth morphologies (Fig 7B). Nonetheless, the positively loaded morphologies diminished significantly from the Campanian to the Danian (*p* = 0.008) and Maastrichtian–Danian (*p* = 0.015) (Table GG in S1 Text). A comparable morphospace shift across the K/Pg interval was detected using the 4-age time-binning scheme (Fig O in S1 Text) and incorporated subtle changes in modal shape within the Campanian and Maastrichtian–Danian-Selandian time-bins (Fig O in S1 Text).

### Regional morphospace

**Fig 8:**
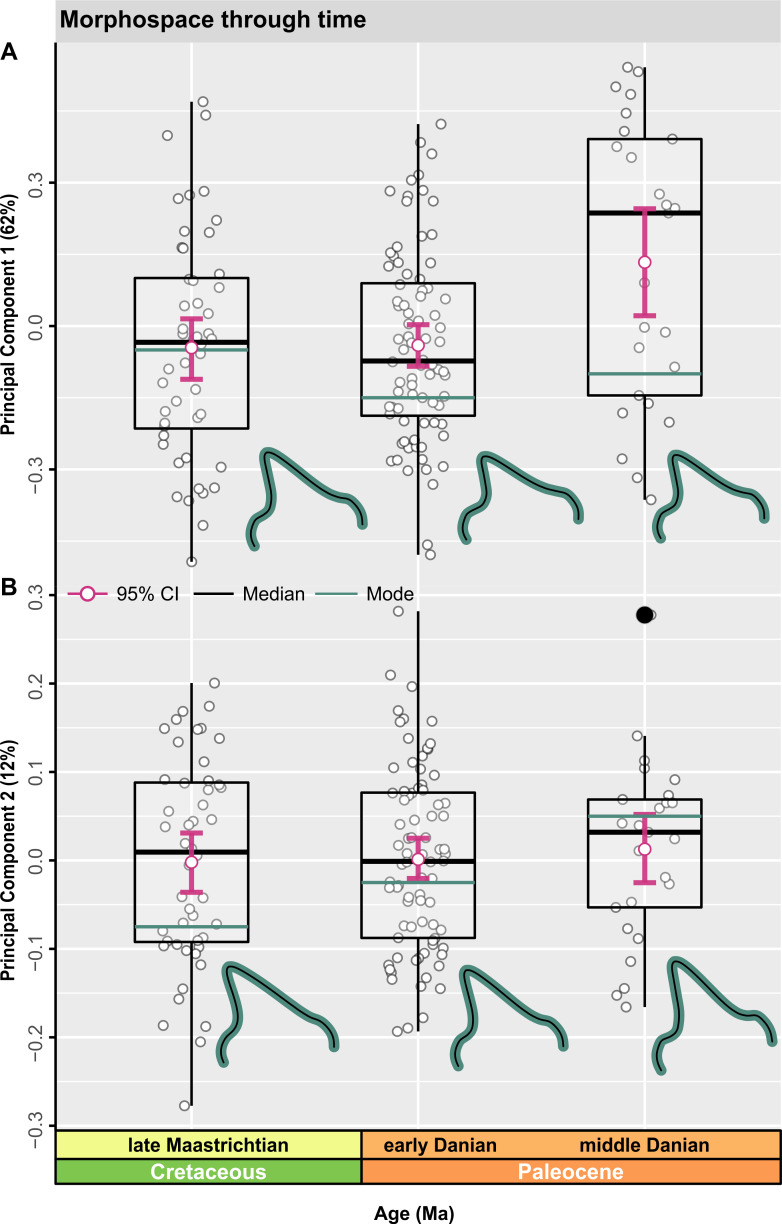
Regional morphospace time series. (A, B) Jittered box plots visualizing the distribution of time-bins along PC1 and PC2 (*n* = 153). Measures of central tendency, including the median (black line) and arithmetic mean (pink), are shown with computed 95% nonparametric bootstrap confidence intervals and potential outliers (closed black points). TPS grids indicate changes in modal value (green line) between time-bins. The data used in this analysis can be accessed online at https://doi.org/10.5061/dryad.c866t1g5n. Ma, million years; TPS, thin plate spline.

The Stevns Klint regional subsample reveals no substantial shifts along PC1 or PC2 during the late Maastrichtian to early Danian (Fig 8A and 8B, Table GG in S1 Text). However, a significant positive sub-age shift in mean morphology occurred along PC1 from the late Maastrichtian to middle Danian (*p* = 0.005), as well as between the early and middle Danian (*p* = 0.005) (Fig 8A, Table GG in S1 Text). Both the late Maastrichtian (*g*1_late Maastrichtian_ *=* 0.2640) and early Danian (*g*1_early Danian_ = 0.2957) are characterized by positively skewed distributions, but with negative skewing during the middle Danian (*g*1_middle Danian_ = −0.1614). An increase in negatively loaded morphologies is associated with the early Danian on PC1 and is further reflected in the modal shape configuration (Fig 8A).

The late Maastrichtian distribution on PC2 is negatively skewed, while the early to middle Danian exhibits positive skewing (Fig 8B, Table GG in S1 Text). Modal shape changes are more pronounced along PC2 (Fig 8B), gradually shifting from negatively to positively loaded values (Fig 8B).

### Superorder-level clade morphospace

**Fig 9:**
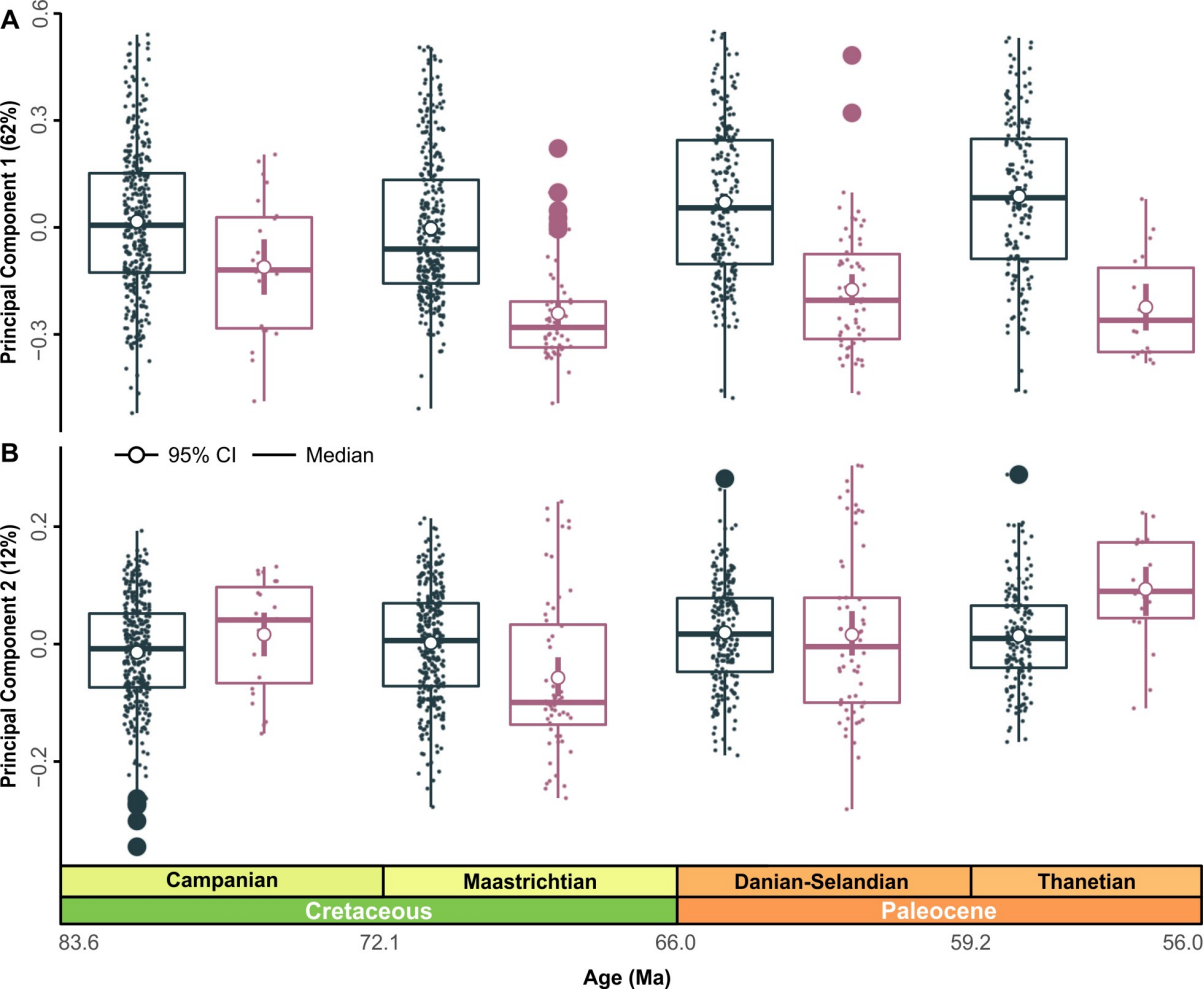
Superorder-level clade morphospace using the global 4-age time-binning scheme. **(A–D)** Jittered grouped box plots visualizing the distribution of time-bins along PC1 and PC2. Graph depicts patterns of overall shape change using the 4-age time-binning scheme. Measures of central tendency, including the median and arithmetic mean, are shown with computed 95% nonparametric bootstrap confidence intervals and potential outliers (closed black points). The data used in this analysis can be accessed online at https://doi.org/10.5061/dryad.c866t1g5n. MA, million years.

Galeomorphs and squalomorphs occupied comparable regions of morphospace on PC1 throughout the entire Maastrichtian–Thanetian interval (Fig 9A); although, some differences were evident in their mean and median values. On average, galeomorphs are characterized by tall and narrow teeth, whereas squalomorph teeth are typically low crowned. The most pronounced shift on PC1 is a reduction in negative values among galeomorphs at the K/Pg boundary (Fig 9A) and mirrored on PC3 and PC4 (Table HH in S1 Text). Squalomorphs likewise exhibited a significant positive shift on PC2 (*p* = 0.016) from the Maastrichtian–Danian-Selandian (Fig 9A and 9B, Table II in S1 Text).

### Order-level clade morphospace

**Fig 10:**
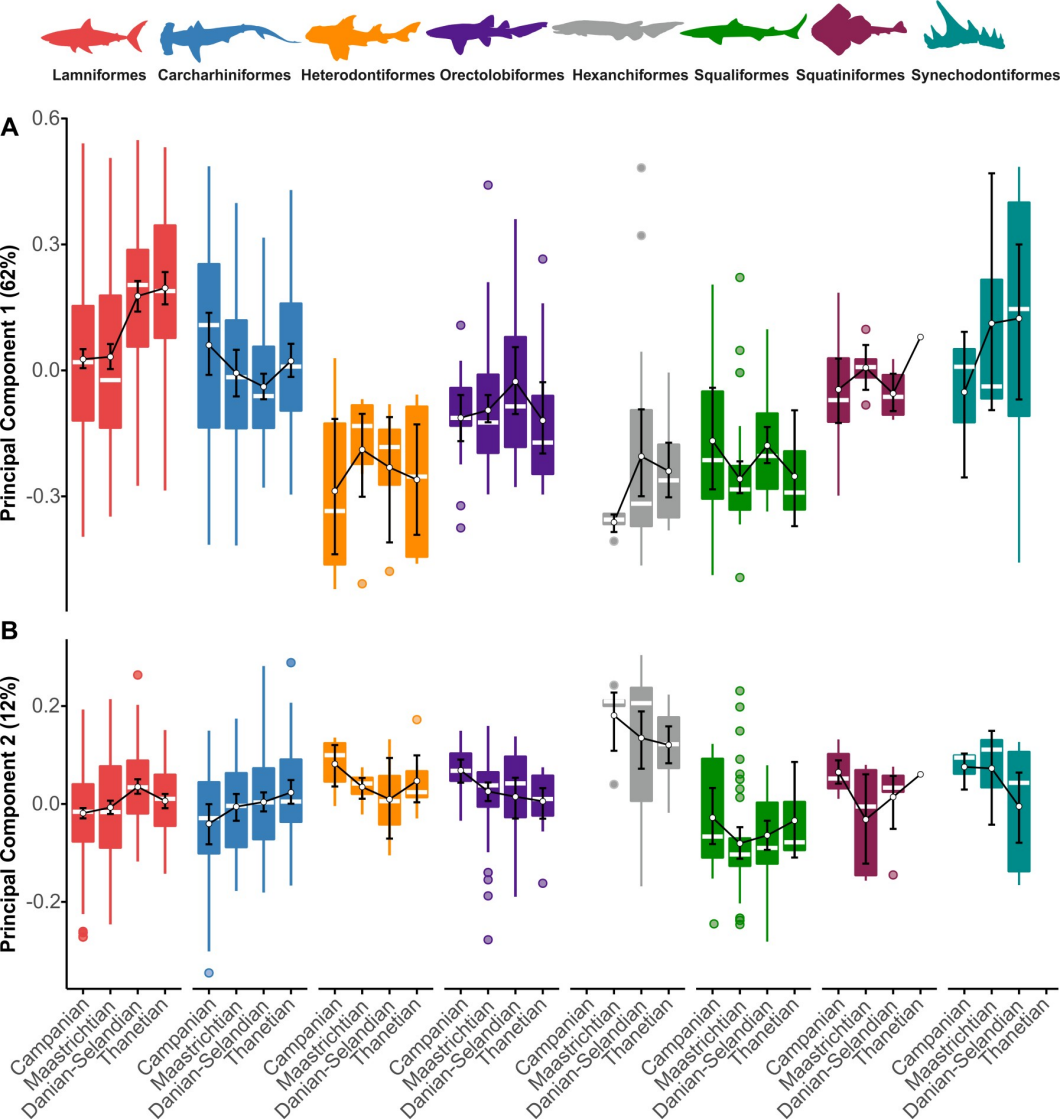
Order-level clade morphospace using the 4-age time-binning scheme. (A, B) Patterns along PC1 and PC2. Sampling was insufficient to estimate reliable disparity for Echinorhiniformes. Measures of central tendency, including the median and arithmetic mean, are shown with computed 95% nonparametric bootstrap confidence intervals and potential outliers. Silhouette graphics created by MB and Julius Csotonyi (https://csotonyi.com/). The data used in this analysis can be accessed online at https://doi.org/10.5061/dryad.c866t1g5n.

Lamniforms displayed a significant positive shift in mean morphology along PC1 from the Maastrichtian–Danian-Selandian (*p* = 0.002) (Fig 10A, Table JJ in S1 Text); this is concurrent with an overall positive to negative shift in their distribution (*g*1_Maastrichitan_ = 0.5035; *g*1_Danian-Selandian_ = −0.3193). The frequency of negatively loaded PC1 morphologies is otherwise reduced during the Thanetian and is significantly different from both the Campanian (*p* = 0.002) and Maastrichtian (*p* = 0.002).

Carcharhiniforms maintained stable morphospace occupation along PC1, but their distribution visibly contracted during the K/Pg transition. This is evidenced by a significant negative shift from the Campanian–Danian-Selandian time-bins (*p* = 0.024) and an overall gradual reduction in positive PC1 values (Fig 10A, Table KK in S1 Text).

Heterodontiforms, orectolobiforms, hexanchiforms, squaliforms, squatiniforms, and †synechodontiforms all produce no significant deviations along PC1 across the Campanian–Thanetian interval (Fig 10A, Tables LL to QQ in S1 Text).

Lamniforms exhibited a significant (*p* = 0.004) depletion of negative values from the Maastrichtian–Danian-Selandian on PC2 (Fig 10B). Pairwise comparisons also recovered a significant difference between the Campanian and the Danian–Selandian (*p* = 0.004). By contrast, carcharhiniforms showed no major changes along PC2, except for a slight increase in positive skewness from the Danian-Selandian–Thanetian; this might imply an exploration of new morphospace (Fig 10B). We additionally detected significant differences between the Campanian and Danian-Selandian (*p* = 0.031) and between the Campanian and Thanetian (*p* = 0.007), although this signal reduced after FDR adjustment of the *p*-values (Table KK in S1 Text).

No shifts were recorded for heterodontiforms or orectolobiforms on PC2 (Fig 10B). Yet, these clades did incline toward positive skewness. Squaliforms, squatiniforms, and †synechodontiforms likewise experienced no significant changes along PC2 (Tables OO to QQ in S1 Text).

## Discussion

### Disparity dynamics across the K/Pg boundary

Fundamentally, our results indicate that the global dental disparity of selachimorphs was largely stable across the end-Cretaceous mass extinction, even though some aspects of variance can be attributed to heterodonty (see Fig 2, Fig N and Table CC in S1 Text). Notably, this static disparity concurs with previous analyses of selachimorph disparity [21] and relative abundance patterns in the elasmobranch fossil record [17]. Conversely, diversity-based assessments advocate up to an approximately 50% species loss among selachimorphs over the same time interval [18,19,76].

Taxic decline [18] with no apparent ecological change is expected for a “nonselective” extinction model [77]. Yet, lamniforms as a dominant group did undergo a “selective” extinction of Cretaceous anacoracids [18,21,25], followed by the radiation of odontaspidids in the Paleocene (Fig 10A and 10B). Selective extinction among lamniforms is documented here through the loss of triangular, blade-like tooth morphologies typical of anacoracids versus the subsequent expansion of apicobasally tall, laterally cusped odontaspidid dental morphospace (Fig 10A and 10B). Such a transition would equate to a “shift” extinction model [21] and coincides with the ecological diversification of carcharhiniforms [21], which additionally invaded mesiodistally broad, low-crowned dental morphospace during the Paleocene (Fig 10A and 10B).

By comparison, squalomorphs were unaffected by the end-Cretaceous mass extinction, although a minor diversity decline is recognized among squalids [18]. We suggest that this group experienced a “nonselective” extinction [77], but with the caveat that their fossil record is poorly sampled (Table A in S1 Text) and should be interpreted cautiously.

Moreover, we emphasize that the proliferation of apicobasally tall dental morphotypes in selachimorphs during the Paleocene does not evince an ecological turnover, unlike the appearance of novel dental grades [78] among coeval actinopterygians [17,20,79–82]. Rather, we propose that while selachimorphs withstood the end-Cretaceous mass extinction, they did suffer sufficient disturbance to trigger a compositional transformation in the morphology of their constituent clades [21,78]—an effect that is best illustrated by the documented fossil histories of lamniforms and carcharhiniforms [21].

### Regional versus global extinction dynamics

Adolfssen and Ward [65] reported a 33% decline in chondrichthyan species richness across the K/Pg boundary based on fossils from Stevns Klint. Notably, this is substantially less than both the approximately 96% species loss calculated from K/Pg boundary deposits in Morocco [83] and the approximately 84% estimate derived from global sampling [18]. Stevns Klint preserves a succession of largely endemic Boreal assemblages [65], yet their static dental disparity is indistinguishable from that of the global sample (Fig 2). Nonetheless, we recognize a minor disparity increase (Fig 2) from the early to middle Danian that, although impacted by sampling as evident from rarefaction, corresponds with alterations in absolute disparity values among galeomorphs (Fig 3A). This slight increase, if real, suggests a region-specific postextinction recovery in the early Paleocene.

A more marked geographic transition occurs in regional sampling “hotspots” (Fig 6), which shift from the WIB of North America during the Campanian and Maastrichtian, to the Scandinavian epeiric basins of Denmark and Sweden by the Danian, and, finally, to the Mediterranean Tethys and Atlantic shelf margins of North Africa and Morocco by the Thanetian. While these results undoubtedly capture preservational biases [84], they correlate with the changing depositional contexts of epicontinental environments across the K/Pg time frame. For example, the late Maastrichtian Western Interior Seaway regression [85] coincides with a regional disparity decline in North America, followed by disparity peaks in the transgressive Tethyan peripheries of Europe and North Africa during the Paleocene. Therefore, we suggest that, like other post-mass extinction marine ecosystems [86–88], the recovery of selachimorphs after the K/Pg event might have been geographically localized and centered on epeiric refugia [65] that provided high-productivity habitats conducive to rediversification.

### Ecological implications

The end-Cretaceous mass extinction disproportionately affected larger-bodied lamniforms [18,19,21,25]. Body size is also implicated in the extinction of coeval marine reptiles, including mosasaurid lizards and plesiosaurians [89–91]. However, unlike these aquatic tetrapods, Cretaceous lamniform apex predators were supplanted by equally large-bodied hexanchiforms in some earliest Paleocene ecosystems [92]. This accords with our morphospace overlap of anacoracids and hexanchids (Fig P in S1 Text) and is further compatible with some modern shark communities, in which the hexanchid, *Notorynchus cepedianus*, is known to invade apex predator niches once vacated by the lamnid, *Carcharodon carcharias* [93]. Unfortunately, shark body size is difficult to estimate accurately from fossil shark teeth [94,95], and the paleoecology of Cretaceous anacoracids and hexanchids is poorly understood. Irrespectively, the persistence of larger-bodied selachimorphs across the K/Pg boundary infers size-based niche continuity, a pattern consistent with static dental disparity (this study and [21]), and, thus, the likelihood that other drivers, including diet, habitat preference, and reproductive strategy [96], were influential in selecting for selachimorph extinction susceptibility. Indeed, the Paleocene radiation of piscivorous odontaspidids, triakids, and scyliorhinids coincided with the diversification of teleosts as an emerging food resource [17]. Correspondingly, we posit that feeding “specialization” might have been key to selective lineage loss [21], as well as the differential survival of more adaptable selachimorph “generalist” predators in the end-Cretaceous mass extinction aftermath.

## Conclusions

Understanding the dynamics of shark evolution across the end-Cretaceous mass extinction event [18,19,21,22,76] has lagged analytically behind assessments of other marine vertebrate groups, such as teleost fishes [17,20,79–82] and aquatic reptiles [90,91,97,98]. Consequently, we present the first multiclade geometric morphometric evaluation of selachimorph disparity based on their extremely abundant dental fossil record. Our principal discovery of overall static disparity indicates that selachimorphs experienced no demonstrable preextinction decline or eco-morphological turnover as postulated for other vertebrate groups [89,90,99,100]. Nevertheless, the dominant Cretaceous anacoracids suffered a selective extinction, captured here by the loss of triangular, blade-like tooth morphologies traditionally associated with apex predator lifestyles (e.g., feeding on larger-bodied aquatic tetrapods [21]). Furthermore, we show that these extinctions are recognizable on both global and regional scales, although geographic shifts in disparity sampling “hotspots” could implicate changing epeiric habitat availability as another delimiting factor.

From a postextinction perspective, while anacoracids disappeared, other lamniform and carcharhiniform groups ecologically proliferated during the Paleocene. Most notably, this affected odontaspidids, triakids, and scyliorhinids, which are characterized by apicobasally tall, laterally cusped teeth. We interpret this as an extinction-mediated ecological “shift” involving significant changes in dental morphology without substantial modifications to selachimorph overall disparity [21]. Coincidentally, the Paleocene diversification of teleosts offers a potential driver, coupled with the dietary adaptability of selachimorphs as opportunistic “generalist” predators capable of exploiting emergent food resources.

Finally, our analyses underscore the utility of morphospace-disparity analyses to complement traditional taxonomy-based assessments of selachimorph diversity [101,102]. We advocate for similar approaches in future assessments of shark evolution but call attention to heterodonty and differential spatiotemporal sampling as sources of variation that likely mask much of the last 250 Ma of selachimorph eco-morphological evolution.

## Supporting information

S1 Text Supplementary Methods and Results. Supplementary Tables: Tables A–VV. Supplementary Figures: Figs A–DD.
(DOCX)

S1 Data Global occurrence database. (XLSX)

S2 Data 2D-landmark coordinate data. (TPS)

S3 Data Sliders file. (TXT)

S4 Data Measurement error landmark coordinate data. (TPS)

S5 Data Annotated R markdown file. (PDF)

S6 Data ReadMe file. (TXT)

S1 Code R markdown file containing reproducible R code for running geometric morphometric and statistical analyses of shark dental morphology. (RMD)

S2 Code R function used to compute a backtransform morphospace. (R)

S3 Code R function used to compute confidence and prediction intervals of the mean, based on a numeric vector. (R)

S4 Code R function used to compute univariate descriptive statistics. (R)

S5 Code R function used to compute and plot minimum convex hulls for a series of specified points. (R)

S6 Code R function used to compute Procrustes variance, bootstrapping, and rarefaction for GPA-aligned data. (R)

S7 Code R function used to generate evenly spaced points from point matrix. (R)

## Abbreviations

EAR: Eastern Atlantic rim
EEB: European epicontinental basin
FDR: false discovery rate
GPA: generalized Procrustes analysis
IQR: interquartile range
K/Pg: Cretaceous/Paleogene
Ma: million years
PCA: principal components analysis
PV: Procrustes variance
RRPP: residual randomization permutation procedure
TPS: thin plate spline
WAR: Western Atlantic rim
WIB: Western Interior basin
2B-PLS: 2-block partial least-squares
