# Supplementary material for: Tooth morphology elucidates shark evolution across the end-Cretaceous mass extinction
Source: PLoS Biol. 2021 Aug 10;19(8):e3001108. doi: 10.1371/journal.pbio.3001108 (PMC8354442; doi:10.1371/journal.pbio.3001108)
Supplement: S1 Text — Supplementary Tables: Tables A–VV. Supplementary Figures: Figs A–DD. (DOCX) [file pbio.3001108.s001.docx]

**S1 Text: Supporting information**

Tooth morphology elucidates shark evolution across the end-cretaceous Mass extinction

Mohamad Bazzi^1 *^, Nicolás E. Campione^2^, Per E. Ahlberg^1^, Henning Blom^1^, Benjamin P. Kear^3 *^

^1^Subdepartment of Evolution and Development, Department of Organismal Biology, Uppsala University, Uppsala, Sweden.

^2^Palaeoscience Research Centre, School of Environmental and Rural Science, University of New England, Armidale, New South Wales, Australia.

^3^Museum of Evolution, Uppsala University, Uppsala, Sweden.

^*^To whom correspondence should be addressed: [mohamad.bazzi@ebc.uu.se](mailto:mohamad.bazzi@ebc.uu.se); [mohamad_bazzi@hotmail.com](mailto:mohamad_bazzi@hotmail.com); [benjamin.kear@em.uu.se](mailto:benjamin.kear@em.uu.se) (lead contact)

**Contents**

This PDF file includes:

Supplementary Methods and Results

Supplementary Tables

Tables A–VV

Supplementary Figures

Figures A–DD

**Other Supplementary Materials for this manuscript include the following:**

S1 Data: Global Occurrence Database (.xlsx)

S2 Data: 2D-Landmark Coordinate Data (.tps)

S3 Data: Sliders File (.txt)

S4 Data: Measurement Error Landmark Coordinate Data (.tps)

S5 Data: Annotated R Markdown File (.pdf)

S6 Data: ReadMe File (.txt)

S1 Code: R Markdown File (.rmd)

S2 Code: R Function (.r)

S3 Code: R Function (.r)

S4 Code: R Function (.r)

S5 Code: R Function (.r)

S6 Code: R Function (.r)

S7 Code: R Function (.r)

**Supplementary Methods**

*Measurement error.* Digitization measurement error is often overlooked in geometric morphometric analyses, and can result in spurious interpretations [1,2]. We therefore quantified measurement error using an intra-class correlation coefficient, which equates to the ratio between repeated measures versus the total variation [2]. Computationally, this is undertaken as a one-way ANOVA. The proportion of measurement error is expressed as:

$$R=S_{A}^{2}/(S_{W}^{2}+S_{A}^{2})\times100$$

where $S_{A}^{2}$ (residual mean sum of squares) is the among-individuals variance, and $S_{W}^{2}$ (group sum of squares) is the within-individuals variance [2,3].

*Heterodonty*. The heterodont dentitions of sharks [4] can influence estimates of their disparity [5]. This is compounded by the usually isolated occurrences of fossil shark teeth, as well as positional variation along the jaw [6], and inconsistent anatomical terminology [4,6,7]. We therefore screened our literature-based image dataset to remove ambiguous descriptive terms (e.g., anterior/parasymphyseal) and inconsistent spelling (e.g., ‘symphyseal’ versus ‘symphysial’). We also treated ‘lateroanterior’ and ‘anterolateral’ as equivalents, but differentiated these from ‘lateroposterior’ and/or ‘posterolateral’. Our standardized categories thus included ‘anterior’, ‘anterolateral’, ‘commissural’, ‘intermediate’, ‘lateral’, ‘lateroposterior’, ‘parasymphyseal’, ‘posterior’, and ‘symphyseal’ (S1 Data).

Six disparity models were used for comparisons:

1. ‘Strict age model’. This provided a two-parameter model (Phenotype ~ Age) whereby the Procrustes variance was calculated for each time-bin (N=1198).
2. ‘Monognathic model’. This provided a three-parameter model (Phenotype ~ Age + Tooth Positions) whereby the Procrustes variance was calculated for each time-bin while accounting for variation in monognathic tooth positions (N=897).
3. ‘A dignathic model’. This provided a three-parameter model (Phenotype ~ Age + Jaw) whereby the Procrustes variance was calculated for each time-bin while accounting for variation in dignathic tooth positions (N=334).
4. ‘Combined model’. This provided a four-parameter model (Phenotype ~ Age + Tooth Positions + Jaw) whereby the Procrustes variance was calculated for each time-bin while accounting for both monognathic and dignathic variation (N=334).
5. ‘Validation model’. This provided a three-parameter model (Phenotype ~ Age + Tooth Positions) whereby the Procrustes variance was calculated for each time-bin while accounting for variation in monognathic tooth positions with all pre-assigned and ‘non-standardised’ tooth positions incorporated into the dataset (N=899).
6. ‘Monognathic and order model’. This provided a four-parameter model (Phenotype ~ Age + Tooth Positions + Orders) whereby the Procrustes variance was calculated for each time-bin while accounting for monognathic variation and order-level differences between time-bins (N=897).

*Collecting bias*. We observed a tendency towards larger tooth sizes being preferentially documented in our literature-based dataset. This was particularly evident among carchariids and odontaspidids, which were represented mainly by ‘anterior’ and ‘lateral’ teeth indicating comparative under-sampling of their dimensionally smaller commissural dentition [6].

**Supplementary results**

*Lagerstätten effects on disparity*. Experimental exclusion of the Stevns Klint regional sub-sample (N=153) resulted in a non-significant (Table RR in S1 Text) increase in global disparity (PV_Maastrichtian_=0.073; PV_Danian-Selandian_=0.079; *p*=0.547) across the Maastrichtian–Danian-Selandian time-bins.

*Lagerstätten effects on morphospace.* Exclusion of the Stevns Klint regional sub-sample produced a significant morphospace shift on PC1–PC3 in the Maastrichtian–Danian time-bins irrespective of time-binning scheme (Table SS in S1 Text).

*Effects of heterodonty on morphospace*. General and axes-specific differences in morphospace were recovered for all monognathic heterodont tooth positions (Fig U in S1 Text, Tables TT and UU in S1 Text). On PC1, both ‘lateroposterior’ and ‘posterior’ tooth positions were negatively loaded, as opposed to the ‘parasymphyseal’ and ‘anterior’ tooth positions, which were positively loaded (Fig U in S1 Text). However, major overlap between all monognathic tooth positions are still visible. On PC2–PC4, the monognathic tooth position distributions were aligned, but with significant differences occurring on PC3 and PC4 (Fig U in S1 Text, panel C and D, Table UU in S1 Text). The effects of dignathic heterodonty were limited to PC2 and PC3 (Fig V in S1 Text, panel B and C). A clade-wise exploration of monognathic heterodonty indicated that tooth position differences were mainly evident along PC1 (Fig W in S1 Text). Furthermore, dignathic heterodonty principally affected lamniforms, hexanchiforms, as well as squaliforms to a lesser degree on PC1–PC4 (Fig W in S1 Text).

**Supplementary TABLES**

**Table A.** Stratigraphic sub-divisions and corresponding sample sizes. Temporal age range (Ma) values are taken from the International Chronostratigraphic Chart v2020/03.

| Era | Period | Age | Temporal range (Ma) | N_selachimorpha_ | N_galeomorphii_ | N_squalmorphii_ |
| --- | --- | --- | --- | --- | --- | --- |
| Cenozoic | Paleogene | Thanetian | 59.2 – 56.0 | 187 | 169 | 18 |
| Cenozoic | Paleogene | Selandian | 61.6 – 59.2 | 28 | 25 | 3 |
| Cenozoic | Paleogene | Danian | 66.0 – 61.6 | 209 | 177 | 32 |
| Mesozoic | Cretaceous | Maastrichtian | 72.1 ± 0.2 – 66.0 | 373 | 317 | 56 |
| Mesozoic | Cretaceous | Campanian | 83.5 – 72.1 | 359 | 336 | 23 |

**Table B.** Sample sizes and dataset proportions for each order-level clade, including †Synechodontiformes.

| Taxon | Common name | Sample size (N) | Proportion (%) |
| --- | --- | --- | --- |
| Lamniformes | Mackerel Sharks | 690 | 55.69 |
| Carcharhiniformes | Ground Sharks | 232 | 18.72 |
| Orectolobiformes | Carpet Sharks | 116 | 9.36 |
| Heterodontiformes | Horn or Bullhead Sharks | 23 | 1.86 |
| Hexanchiformes | Cow and Frilled Sharks | 43 | 3.47 |
| Squaliformes | Dogfish Sharks | 89 | 7.18 |
| Echinorhiniformes | Bramble Sharks | 6 | 0.48 |
| Squatiniformes | Angel Sharks | 23 | 1.86 |
| †Synechodontiformes | - | 17 | 1.37 |
| Total | - | 1239 | - |

**Table C**. Time-binning schemes and sample sizes for galeomorph order-level clades.

| Binning scheme | Time-bins | N_lamniformes_ | N_carcharhiniformes_ | N_orectolobiformes_ | N_heterodontiformes_ |
| --- | --- | --- | --- | --- | --- |
| Five-bin analysis | Campanian | 276 | 34 | 17 | 6 |
|  | Maastrichtian | 198 | 46 | 63 | 7 |
|  | Danian | 64 | 80 | 21 | 4 |
|  | Selandian | 23 | 2 | 0 | 0 |
|  | Thanetian | 82 | 66 | 15 | 6 |
|  | Total | 643 | 228 | 116 | 23 |
| Four-bin analysis | Campanian | 276 | 34 | 17 | 6 |
|  | Maastrichtian | 198 | 46 | 63 | 7 |
|  | Danian-Selandian | 104 | 82 | 21 | 4 |
|  | Thanetian | 82 | 66 | 15 | 6 |
|  | Total | 660 | 228 | 116 | 23 |

**Table D.** Time-binning schemes and sample sizes for squalomorph order-level clades, including †Synechodontiformes. Echinorhiniformes was excluded because of insufficient sample size. D-S = Danian-Selandian.

| Disparity through time | Time-bins | N_Hexanchiformes_ | N_squaliformes_ | N_squatiniformes_ | N_synechodontiformes_ |
| --- | --- | --- | --- | --- | --- |
| Five-bin analysis | Campanian | 0 | 12 | 11 | 3 |
|  | Maastrichtian | 5 | 40 | 5 | 3 |
|  | Danian | 13 | 13 | 6 | 8 |
|  | Selandian | 0 | 3 | 0 | 0 |
|  | Thanetian | 14 | 3 | 1 | 0 |
|  | Total | 32 | 71 | 22 | 14 |
| Four-bin analysis | Campanian | 0 | 12 | 11 | 3 |
|  | Maastrichtian | 5 | 40 | 5 | 3 |
|  | D-S | 21 | 31 | 6 | 10 |
|  | Thanetian | 14 | 3 | 1 | 0 |
|  | Total | 40 | 86 | 23 | 16 |

**Table E.** Time-binning schemes and sample sizes for superorder-level clades.

| Disparity through time | Time-bins | N_selachimorpha_ | N_galeomorphii_ | N_squalomorphii_ |
| --- | --- | --- | --- | --- |
| Sub-stage level analysis | early Campanian | 149 | 134 | 15 |
|  | late Campanian | 23 | 17 | - |
|  | early Maastrichtian | 98 | 86 | 12 |
|  | late Maastrichtian | 218 | 194 | 24 |
|  | early Danian | 80 | 61 | 19 |
|  | middle Danian | 80 | 76 | - |
|  | late Danian | 11 | - | - |
|  | Total | 659 | 568 | 70 |

**Table F.** Number, type and description of geometrically homologous fixed landmarks and sliding semi-landmarks. ‘Type 1’ and ‘Type 2’ designations refer to Bookstein [8].

| Landmark | Description | Bookstein’s Typology |
| --- | --- | --- |
| LM1 | Junction of left cusp edge | Type 1 |
| LM2 | Junction of right cusp edge | Type 1 |
| LM3 | Cusp apex | Type 2 |
| Curve | Description | Sliding semi-landmark |
| C1 | Distal margin | N = 79 |
| C2 | Mesial margin | N = 81 |

**Table G.** Selected selachimorph tooth specimens used to assess digitization measurement error.

| Order | Family | Genus | Species | Dental Unit | Specimen No. | Relative Position | Source |
| --- | --- | --- | --- | --- | --- | --- | --- |
| Lamniformes | Archaeolamnidae | *Archaeolamna* | *kopingensis judithensis* | upper | UM HBR 64 | anterolateral | Cappetta 2012 |
| Lamniformes | Otodontidae | *Cretolamna* | *maroccana* | upper | UM-BEG-C2.21 | lateral | Cappetta *et al*. 2014b |
| Lamniformes | Odontaspididae | *Glueckmanotodus* | *heinzelini* | - | UM DOR 1 | Posterior | Cappetta 2012 |
| Lamniformes |  | *Palaeocarcharodon* | *orientalis* | - | - | anterior | Cappetta 2012 |
| Lamniformes | Anacoracidae | *Squalicorax* | *africanus* | - | UM-CHA1-1 | anterior | Bardet *et al*. 2000 |
| Carcharhiniformes | Carcharhinidae | *Danogaleus* | *gueriri* | - | UM BOD 8 | lateral | Cappetta 2012 |
| Carcharhiniformes | Carcharhinidae | *Danogaleus* | *gueriri* | - | UM BOD 10 | lateral | Cappetta 2012 |
| Carcharhiniformes | Triakidae | *Palaeogaleus* | *navarroensis* nov sp. | - | AMNH 13710 | lateral | Case & Cappetta 1997 |
| Carcharhiniformes | Scyliorhinidae | *Scyliorhinus* | *monsaugustus* sp nov | - | MAO2-25 | anterior | Guinot *et al*. 2013 |
| Carcharhiniformes | Scyliorhinidae | *Scyliorhinus* | *monsaugustus* sp nov | - | MAO2-27 | posterior | Guinot *et al*. 2013 |
| Orectolobiformes | Hemiscylliidae | *Chiloscyllium* | *frequens* sp. nov. | - | P. 67019 | anterior | Guinot *et al*. 2013 |
| Orectolobiformes | Hemiscylliidae | *Chiloscyllium* | *frequens* sp. nov. | - | P. 67022 | lateral | Guinot *et al*. 2013 |
| Orectolobiformes | Hemiscylliidae | *Chiloscyllium* | *frequens* sp. nov. | - | P. 67020 | lateroposterior | Guinot *et al*. 2013 |
| Orectolobiformes | Ginglymostomatidae | *Plicatoscyllium* | *lehneri* | - | - | anterolateral | Cappetta 2012 |
| Orectolobiformes | Ginglymostomatidae | *Plicatoscyllium* | *lehneri* | - | - | lateral | Cappetta 2012 |
| Heterodontiformes | Heterodontidae | *Heterodontus* | *boussioni* sp nov | - | HAL2-7 | lateral | Guinot *et al*. 2013 |
| Heterodontiformes | Heterodontidae | *Heterodontus* | *creamridgensis* | - | WBR 95 | anterior | Case & Cappetta 2004 |
| Heterodontiformes | Heterodontidae | *Heterodontus* | *rugosus* | - | MGUH 29939 | anterior | Adolfssen & Ward 2014 |
| Heterodontiformes | Heterodontidae | *Heterodontus* | *rugosus* | - | MGUH 29941 | lateral | Adolfssen & Ward 2014 |
| Heterodontiformes | Heterodontidae | *Heterodontus* | sp. 2 | - | MAO2-12 | lateral | Guinot *et al*. 2013 |
| Squaliformes | Somniosidae | *Centroscymnus* | *praecursor* | lower | MGUH 29910 | - | Adolfssen & Ward 2014 |
| Squaliformes | Squalidae | *Megasqualus* | *orpiensis* | - | UM MAR 6 | anterior | Cappetta 2012 |
| Squaliformes | Squalidae | *Megasqualus* | *orpiensis* | - | UM MAR 8 | lateral | Cappetta 2012 |
| Squaliformes | Squalidae | *Protosqualus* | *argentinensis* nov sp | - | MPM 10023 | anterolateral | Bogan *et al*. 2016 |
| Squaliformes | Squalidae | *Squalus* | *gabrielsoni* | - | MGUH 29901 | lateral | Adolfssen & Ward 2014 |
| Hexanchiformes | Hexanchidae | *Heptranchias* | sp | lower | LO 849 t | anterolateral | Siverson 1995 |
| Hexanchiformes | Hexanchidae | *Notidanodon* | *loozi* | lower | IRSNB P.1512 | symphyseal | Cappetta 2012 |
| Hexanchiformes | Hexanchidae | *Notidanodon* | sp. | upper | UM DAO 1-C | anterolateral | Cappetta 2012 |
| Hexanchiformes | Hexanchidae | *Notidanodon* | sp. | upper | UM DAO 1-D | anterolateral | Cappetta 2012 |
| Hexanchiformes | Hexanchidae | *Notorynchus* | sp. | upper | LO 848 t | anterolateral | Siverson 1995 |

**Table H.** Generalized Procrustes Analysis (GPA) with partial Procrustes superimposition. GPA was performed using different iterations to evaluate algorithm convergence. Q-values between 4.35–4.09 (iteration-level = 6 to 9) produced comparable alignment.

| Fixed landmarks | Number of semi-landmarks | P | M | Q-value | GPA iterations | Sliding method |
| --- | --- | --- | --- | --- | --- | --- |
| - | - | - | - | 6.183326 | 3 | BE |
| - | - | - | - | 4.448685 | 4 | BE |
| - | - | - | - | 4.423181 | 5 | BE |
| 3 | 157 (sliders) | 160 | 2D | 4.357470 | 6 | BE |
| - | - | - | - | 4.297608 | 7 | BE |
| - | - | - | - | 4.207907 | 8 | BE |
| - | - | - | - | 4.098833 | 9 | BE |
| - | - | - | - | 3.959336 | 10 | BE |
| - | - | - | - | 3.785426 | 11 | BE |
| - | - | - | - | 3.584394 | 12 | BE |
| - | - | - | - | 3.350921 | 13 | BE |
| - | - | - | - | 3.098619 | 14 | BE |
| - | - | - | - | 2.827423 | 15 | BE |
| - | - | - | - | 2.543247 | 16 | BE |
| - | - | - | - | 2.261878 | 17 | BE |
| - | - | - | - | 1.989550 | 18 | BE |
| - | - | - | - | 1.964127 | 19 | BE |
| - | - | - | - | 1.335368 | 20 | BE |
| - | - | - | - | 1.658891 | 21 | BE |

BE = Minimized Bending Energy.

**Table I.** Repeatability of digitized landmarks. Shape variation = 99%; digitization and measurement error = ~1%.

|  | *d.f.* | SS | MS | R^2^ | F | Z | Pr(>F) |
| --- | --- | --- | --- | --- | --- | --- | --- |
| Individuals | 29 | 3.9346 | 0.135674 | 0.98903 | 105.2969 | 14.5352 | 0.001 |
| Replicates | 1 | 0.0063 | 0.006258 | 0.00157 | 4.8565 | 3.0475 | 0.002 |
| Residuals | 29 | 0.0374 | 0.001288 | 0.00939 |  |  |  |
| Total | 59 | 3.9782 |  |  |  |  |  |

**Table J.** Principal Components Analysis (PCA) of the digitized landmark data. Computed variance (eigenvalues, λ_k_) explained by the first 10 PC-axes (eigenvectors, u_k_). PCA eigenvectors = 320.

| Principal Component (u_k)_ | Standard Deviation (σ) | Proportion of Variance (%) | Cumulative Proportion |
| --- | --- | --- | --- |
| PC1 | 0.2222 | 61.736 | 0.6174 |
| PC2 | 0.09869 | 12.180 | 0.73915 |
| PC3 | 0.09269 | 10.743 | 0.84658 |
| PC4 | 0.06080 | 4.623 | 0.89281 |
| PC5 | 0.04459 | 2.487 | 0.91767 |
| PC6 | 0.03464 | 1.500 | 0.93267 |
| PC7 | 0.03248 | 1.319 | 0.94586 |
| PC8 | 0.02645 | 0.875 | 0.95461 |
| PC9 | 0.02395 | 0.717 | 0.96179 |
| PC10 | 0.02238 | 0.626 | 0.96805 |

**Table K.** Descriptive and test statistics of order-level clades in morphospace.

| PC-axis | Clade | N | Mean ($\bar{x}$) | Median ($\tilde{x}$) | Shapiro.W | Shapiro.p | Dip.Test.D | Dip.Test.p | Skewness |
| --- | --- | --- | --- | --- | --- | --- | --- | --- | --- |
|  | Lamniformes |  |  |  |  |  |  |  |  |
| 1 |  | 690 | 0.07804863 | 0.070565597 | 0.9831824 | 4.002893e-07 | 0.01448571 | 0.4457993 | 0.14653437 |
| 2 |  | 690 | -0.00306696 | -0.001908225 | 0.9950210 | 2.456395e-02 | 0.01401653 | 0.5053166 | -0.03982309 |
|  | Carcharhiniformes |  |  |  |  |  |  |  |  |
| 1 |  | 232 | -0.0013767133 | -0.034126110 | 0.9738609 | 0.0002750076 | 0.01769094 | 0.9306216 | 0.41108852 |
| 2 |  | 232 | 0.0004572805 | -0.004317489 | 0.9926368 | 0.3021120732 | 0.03006935 | 0.1522842 | -0.05334797 |
|  | Heterodontiformes |  |  |  |  |  |  |  |  |
| 1 |  | 23 | -0.24043833 | -0.19821966 | 0.8645787 | 0.005058779 | 0.10729258 | 0.02080989 | -0.36321374 |
| 2 |  | 23 | 0.04587881 | 0.03401269 | 0.9748870 | 0.803614529 | 0.05664513 | 0.83150375 | -0.05576618 |
|  | Orectolobiformes |  |  |  |  |  |  |  |  |
| 1 |  | 116 | -0.08829770 | -0.12129393 | 0.9289468 | 1.142309e-05 | 0.02086442 | 0.9906737 | 1.054916 |
| 2 |  | 116 | 0.02734313 | 0.04561915 | 0.9097573 | 9.223353e-07 | 0.02530561 | 0.9068080 | -1.269113 |
|  | Hexanchiformes |  |  |  |  |  |  |  |  |
| 1 |  | 43 | -0.2457358 | -0.3389045 | 0.7663057 | 7.27881e-07 | 0.03307539 | 0.9905036 | 1.9166473 |
| 2 |  | 43 | 0.1390947 | 0.1739687 | 0.9295460 | 1.12184e-02 | 0.03845325 | 0.9360404 | -0.7702537 |
|  | Squaliformes |  |  |  |  |  |  |  |  |
| 1 |  | 89 | -0.21898115 | -0.26686095 | 0.8973898 | 3.457211e-06 | 0.02559129 | 0.9733294 | 1.1367165 |
| 2 |  | 89 | -0.06642451 | -0.09510701 | 0.9370546 | 3.140320e-04 | 0.02268368 | 0.9920183 | 0.7381424 |
|  | Echinorhiniformes |  |  |  |  |  |  |  |  |
| 1 |  | 6 | -0.2420685 | -0.2445328 | 0.9680474 | 0.8790709 | 0.11510577 | 0.5867129 | 0.3005130 |
| 2 |  | 6 | -0.1227031 | -0.1954872 | 0.8429049 | 0.1377664 | 0.09042546 | 0.8364397 | 0.8304803 |
|  | Squatiniformes |  |  |  |  |  |  |  |  |
| 1 |  | 23 | -0.03117527 | -0.01648834 | 0.9691875 | 0.66955901 | 0.07647978 | 0.3212052 | -0.2157085 |
| 2 |  | 23 | 0.03056737 | 0.04240456 | 0.8140890 | 0.00064963 | 0.06131238 | 0.7174112 | -1.3377591 |
|  | †Synechodontiformes |  |  |  |  |  |  |  |  |
| 1 |  | 17 | 0.07599271 | 0.008601875 | 0.9353278 | 0.266867293 | 0.09791731 | 0.1649063 | 0.05505126 |
| 2 |  | 17 | 0.03229257 | 0.091273980 | 0.8385699 | 0.007213756 | 0.07812923 | 0.5200557 | -0.76225723 |

**Table L.** Procrustes Variance (PV) and associated interval estimations (95% prediction and confidence limits) for Selachimorpha (including †Synechodontiformes). Interval estimates were generated using bootstrapping with 999 iterations.

|  | Raw Disparity | Lower.PI | Upper.PI | Lower.CI | Upper.CI |
| --- | --- | --- | --- | --- | --- |
| **Five-stage binning scheme** |  |  |  |  |  |
| Campanian | 0.06911023 | 0.0627 | 0.0752 | 0.0687 | 0.0691 |
| Maastrichtian | 0.07584028 | 0.0693 | 0.0819 | 0.0754 | 0.0758 |
| Danian | 0.08275046 | 0.0727 | 0.0922 | 0.0821 | 0.0827 |
| Selandian | 0.04272476 | 0.0254 | 0.0566 | 0.0405 | 0.0415 |
| Thanetian | 0.08535443 | 0.0757 | 0.0943 | 0.0847 | 0.0853 |
| **Four-stage binning scheme** |  |  |  |  |  |
| Danian-Selandian | 0.08439832 | 0.0754 | 0.0922 | 0.0835 | 0.0841 |

PI = Prediction, CI = Confidence.

**Table M.** PV and associated interval estimations (95% prediction and confidence limits) for Selachimorpha (excluding †Synechodontiformes) using the four-age time-binning scheme. Interval estimates were generated using bootstrapping with 999 iterations.

|  | Raw Disparity | Lower.PI | Upper.PI | Lower.CI | Upper.CI |
| --- | --- | --- | --- | --- | --- |
| **Four-age time-binning scheme** |  |  |  |  |  |
| Campanian | 0.06921892 | 0.0620 | 0.0757 | 0.0686 | 0.0691 |
| Maastrichtian | 0.07537421 | 0.0689 | 0.0818 | 0.0752 | 0.0756 |
| Danian-Selandian | 0.08217259 | 0.0739 | 0.0901 | 0.0818 | 0.0823 |
| Thanetian | 0.08535443 | 0.0752 | 0.0943 | 0.0844 | 0.0851 |

PI = Prediction, CI = Confidence.

**Table N.** PV and associated interval estimations (95% prediction and confidence limits) for Selachimorpha using the Stevns Klint regional sub-sample and a three-age time-binning scheme. Interval estimates were generated using bootstrapping with 999 iterations.

|  | Raw Disparity | Lower.PI | Upper.PI | Lower.CI | Upper.CI |
| --- | --- | --- | --- | --- | --- |
| **Regional three-age time-binning scheme** |  |  |  |  |  |
| late Maastrichtian | 0.09190902 | 0.0713 | 0.1097 | 0.0899 | 0.0911 |
| early Danian | 0.06939083 | 0.0566 | 0.0810 | 0.0684 | 0.0692 |
| middle Danian | 0.12391158 | 0.0871 | 0.1502 | 0.1176 | 0.1196 |

PI = Prediction, CI = Confidence.

**Table O.** PV and associated interval estimations (95% prediction and confidence limits) for Galeomorphii (including †Synechodontiformes) using the four-age time-binning scheme. Interval estimates were generated using bootstrapping with 999 iterations.

|  | Raw Disparity | Lower.PI | Upper.PI | Lower.CI | Upper.CI |
| --- | --- | --- | --- | --- | --- |
| **Four-age time-binning scheme** |  |  |  |  |  |
| Campanian | 0.06826546 | 0.0616 | 0.0747 | 0.0680 | 0.0684 |
| Maastrichtian | 0.06802529 | 0.0616 | 0.0741 | 0.0677 | 0.0681 |
| Danian-Selandian | 0.07309997 | 0.0655 | 0.0803 | 0.0727 | 0.0731 |
| Thanetian | 0.07741032 | 0.0674 | 0.0864 | 0.0766 | 0.0772 |

PI = Prediction, CI = Confidence.

**Table P.** PV and associated interval estimations (95% prediction and confidence limits) for Galeomorphii (excluding †Synechodontiformes) using the four-age time-binning scheme. Interval estimates were generated using bootstrapping with 999 iterations.

|  | Raw Disparity | Lower.PI | Upper.PI | Lower.CI | Upper.CI |
| --- | --- | --- | --- | --- | --- |
| **Four-age time-binning scheme** |  |  |  |  |  |
| Campanian | 0.06835569 | 0.0613 | 0.0748 | 0.0678 | 0.0683 |
| Maastrichtian | 0.06750400 | 0.0608 | 0.0739 | 0.0671 | 0.0676 |
| Danian-Selandian | 0.07009500 | 0.0625 | 0.0770 | 0.0695 | 0.0700 |
| Thanetian | 0.07741032 | 0.0682 | 0.0861 | 0.0769 | 0.0774 |

**Table Q.** PV and associated interval estimations (95% prediction and confidence limits) for Squalomorphii using the four-age time-binning scheme. Interval estimates were generated using bootstrapping with 999 iterations.

|  | Raw Disparity | Lower.PI | Upper.PI | Lower.CI | Upper.CI |
| --- | --- | --- | --- | --- | --- |
| **Four-age time-binning scheme** |  |  |  |  |  |
| Campanian | 0.05885412 | 0.0399 | 0.0726 | 0.0558 | 0.0568 |
| Maastrichtian | 0.06656151 | 0.0504 | 0.0805 | 0.0650 | 0.0659 |
| Danian-Selandian | 0.07742955 | 0.0559 | 0.0962 | 0.0754 | 0.0767 |
| Thanetian | 0.05885900 | 0.0432 | 0.0682 | 0.0553 | 0.0561 |

PI = Prediction, CI = Confidence.

**Table R.** PV and associated interval estimations (95% prediction and confidence limits) for Galeomorphii (including †Synechodontiformes) using the Stevns Klint regional sub-sample and a three-age time-binning scheme. Interval estimates were generated using bootstrapping with 999 iterations.

|  | Raw Disparity | Lower.PI | Upper.PI | Lower.CI | Upper.CI |
| --- | --- | --- | --- | --- | --- |
| **Regional three-age time-binning scheme** |  |  |  |  |  |
| late Maastrichtian | 0.07965242 | 0.0551 | 0.0987 | 0.0762 | 0.0776 |
| early Danian | 0.07158083 | 0.0566 | 0.0845 | 0.0701 | 0.0710 |
| middle Danian | 0.10483342 | 0.0761 | 0.1245 | 0.0995 | 0.1011 |

PI = Prediction, CI = Confidence.

**Table S.** PV and associated interval estimations (95% prediction and confidence limits) for Galeomorphii (excluding †Synechodontiformes) using the Stevns Klint regional sub-sample and a three-age time-binning scheme. Interval estimates were generated using bootstrapping with 999 iterations.

|  | Raw Disparity | Lower.PI | Upper.PI | Lower.CI | Upper.CI |
| --- | --- | --- | --- | --- | --- |
| **Regional three-age time-binning scheme** |  |  |  |  |  |
| late Maastrichtian | 0.07433491 | 0.0526 | 0.0917 | 0.0715 | 0.0728 |
| early Danian | 0.06924778 | 0.0541 | 0.0815 | 0.0673 | 0.0682 |
| middle Danian | 0.10923748 | 0.0736 | 0.1303 | 0.1010 | 0.1028 |

PI = Prediction, CI = Confidence.

**Table T.** PV and associated interval estimations (95% prediction and confidence limits) for Squalomorphii using the Stevns Klint regional sub-sample and a three-age time-binning scheme. Interval estimates were generated using bootstrapping with 999 iterations.

|  | Raw Disparity | Lower.PI | Upper.PI | Lower.CI | Upper.CI |
| --- | --- | --- | --- | --- | --- |
| **Regional three-age time-binning scheme** |  |  |  |  |  |
| late Maastrichtian | 0.07756513 | 0.0537 | 0.0931 | 0.0728 | 0.0740 |
| early Danian | 0.04105018 | 0.0312 | 0.0466 | 0.0387 | 0.0392 |
| middle Danian | 0.02008752 | -0.0101 | 0.0293 | 0.0090 | 0.0102 |

PI = Prediction, CI = Confidence.

**Table U.** PV and associated interval estimations (95% prediction and confidence limits) for Lamniformes (n=690) using the four-age time-binning scheme. Interval estimates were generated using bootstrapping with 999 iterations.

|  | Raw Disparity | Lower.PI | Upper.PI | Lower.CI | Upper.CI |
| --- | --- | --- | --- | --- | --- |
| **Four-age time-binning scheme** |  |  |  |  |  |
| Campanian | 0.06345675 | 0.0564 | 0.0696 | 0.0628 | 0.0632 |
| Maastrichtian | 0.07041109 | 0.0630 | 0.0773 | 0.0699 | 0.0704 |
| Danian-Selandian | 0.05972992 | 0.0496 | 0.0685 | 0.0587 | 0.0593 |
| Thanetian | 0.05562265 | 0.0451 | 0.0642 | 0.0544 | 0.0550 |

PI = Prediction, CI = Confidence.

**Table V.** PV and associated interval estimations (95% prediction and confidence limits) for Carcharhiniformes (n=232) using the four-age time-binning scheme. Interval estimates were generated using bootstrapping with 999 iterations.

|  | Raw Disparity | Lower.PI | Upper.PI | Lower.CI | Upper.CI |
| --- | --- | --- | --- | --- | --- |
| **Four-age time-binning scheme** |  |  |  |  |  |
| Campanian | 0.08523550 | 0.0629 | 0.1026 | 0.0821 | 0.0834 |
| Maastrichtian | 0.05627971 | 0.0415 | 0.0677 | 0.0542 | 0.0550 |
| Danian-Selandian | 0.04167265 | 0.0338 | 0.0485 | 0.0409 | 0.0413 |
| Thanetian | 0.06197006 | 0.0509 | 0.0712 | 0.0607 | 0.0614 |

PI = Prediction, CI = Confidence.

**Table W.** PV and associated interval estimations (95% prediction and confidence limits) for Heterodontiformes (n=23) using the four-age time-binning scheme. Interval estimates were generated using bootstrapping with 999 iterations.

|  | Raw Disparity | Lower.PI | Upper.PI | Lower.CI | Upper.CI |
| --- | --- | --- | --- | --- | --- |
| **Four-age time-binning scheme** |  |  |  |  |  |
| Campanian | 0.05642643 | 0.0175 | 0.0765 | 0.0461 | 0.0480 |
| Maastrichtian | 0.04315409 | 0.0088 | 0.0664 | 0.0367 | 0.0385 |
| Danian-Selandian | 0.05059766 | 0.0114 | 0.0656 | 0.0376 | 0.0394 |
| Thanetian | 0.05693581 | 0.0210 | 0.0730 | 0.0462 | 0.0478 |

PI = Prediction, CI = Confidence.

**Table X.** PV and associated interval estimations (95% prediction and confidence limits) for Orectolobiformes (n=116) using the four-age time-binning scheme. Interval estimates were generated using bootstrapping with 999 iterations.

|  | Raw Disparity | Lower.PI | Upper.PI | Lower.CI | Upper.CI |
| --- | --- | --- | --- | --- | --- |
| **Four-age time-binning scheme** |  |  |  |  |  |
| Campanian | 0.02406313 | 0.0128 | 0.0322 | 0.0222 | 0.0228 |
| Maastrichtian | 0.04110695 | 0.0290 | 0.0518 | 0.0400 | 0.0408 |
| Danian-Selandian | 0.06491247 | 0.0446 | 0.0804 | 0.0619 | 0.0631 |
| Thanetian | 0.05674483 | 0.0332 | 0.0744 | 0.0531 | 0.0544 |

PI = Prediction, CI = Confidence.

**Table Y.** PV and associated interval estimations (95% prediction and confidence limits) for Hexanchiformes (n=43) using the three-age time-binning scheme. Interval estimates were generated using bootstrapping with 999 iterations.

|  | Raw Disparity | Lower.PI | Upper.PI | Lower.CI | Upper.CI |
| --- | --- | --- | --- | --- | --- |
| **Ages** |  |  |  |  |  |
| Maastrichtian | 0.01846043 | 0.0012 | 0.0286 | 0.0145 | 0.0154 |
| Danian-Selandian | 0.12424372 | 0.0672 | 0.1684 | 0.1162 | 0.1194 |
| Thanetian | 0.05400864 | 0.0333 | 0.0667 | 0.0495 | 0.0505 |

PI = Prediction, CI = Confidence.

**Table Z.** PV and associated interval estimations (95% prediction and confidence limits) for Squaliformes (n=86) using the four-age time-binning scheme. Interval estimates were generated using bootstrapping with 999 iterations.

|  | Raw Disparity | Lower.PI | Upper.PI | Lower.CI | Upper.CI |
| --- | --- | --- | --- | --- | --- |
| **Four-age time-binning scheme** |  |  |  |  |  |
| Campanian | 0.07059085 | 0.0347 | 0.0948 | 0.0638 | 0.0657 |
| Maastrichtian | 0.05435441 | 0.0331 | 0.0728 | 0.0523 | 0.0536 |
| Danian-Selandian | 0.03277852 | 0.0239 | 0.0395 | 0.0315 | 0.0320 |
| Thanetian | 0.03350868 | -0.0057 | 0.0506 | 0.0216 | 0.0234 |

PI = Prediction; CI = Confidence.

**Table AA.** PV and associated interval estimations (95% prediction and confidence limits) for Squatiniformes (n=23) using the three-age time-binning scheme. Interval estimates were generated using bootstrapping with 999 iterations.

|  | Raw Disparity | Lower.PI | Upper.PI | Lower.CI | Upper.CI |
| --- | --- | --- | --- | --- | --- |
| **Ages** |  |  |  |  |  |
| Campanian | 0.02448442 | 0.0098 | 0.0351 | 0.0220 | 0.0228 |
| Maastrichtian | 0.01809533 | 0.0072 | 0.0219 | 0.0143 | 0.0148 |
| Danian-Selandian | 0.01412439 | 0.0038 | 0.0198 | 0.0116 | 0.0121 |

PI = Prediction, CI = Confidence.

**Table BB.** PV and associated interval estimations (95% prediction and confidence limits) for †Synechodontiformes (n=17) using the three-age time-binning scheme. Interval estimates were generated using bootstrapping with 999 iterations.

|  | Raw Disparity | Lower.PI | Upper.PI | Lower.CI | Upper.CI |
| --- | --- | --- | --- | --- | --- |
| **Ages** |  |  |  |  |  |
| Campanian | 0.0300658 | -0.0050 | 0.0458 | 0.0196 | 0.0212 |
| Maastrichtian | 0.1000783 | -0.0178 | 0.1490 | 0.0630 | 0.0682 |
| Danian-Selandian | 0.1283956 | 0.0583 | 0.1748 | 0.1147 | 0.1184 |

PI = Prediction, CI = Confidence.

**Table CC.** Effect of monognathic and dignathic heterodonty on temporal disparity using the four-age time-binning scheme. Pairwise differences between groups (i.e. time-bins) are based on RRPP. Upper off-diagonal cells depict FDR-adjusted P-values; lower off-diagonal cells depict raw P-values. Bold values indicate significant difference between treatment means at α=0.05.

|  | Disparity Models | | | | | | | | | | | |
| --- | --- | --- | --- | --- | --- | --- | --- | --- | --- | --- | --- | --- |
|  | Shape ~ Position + Time  (N=897) | | | | Shape ~ Unit + Time  (N=334) | | | | Shape ~ Position + Unit + Time  (N=334) | | | |
|  | C | M | D–S | T | C | M | D–S | T | C | M | D–S | T |
| C | - | 0.371 | **0.008** | 0.133 | - | 0.184 | **0.016** | 0.184 | - | **0.016** | **0.008** | **0.027** |
| M | 0.232 | - | **0.032** | 0.417 | 0.081 | - | 0.258 | 1.000 | **0.004** | - | 0.793 | 0.793 |
| D-S | **0.001** | **0.008** | - | 0.218 | **0.002** | 0.161 | - | 0.184 | **0.001** | 0.548 | - | 0.468 |
| T | 0.050 | 0.313 | 0.109 | - | 0.092 | 0.901 | 0.091 | - | **0.010** | 0.595 | 0.234 | - |

C = Campanian, M = Maastrichtian, D-S = Danian–Selandian, T = Thanetian.

N = Total sample size.

**Table DD.** PV obtained from models incorporating monognathic and dignathic heterodonty in the four-age time-binning scheme.

|  | Raw Disparity | | |
| --- | --- | --- | --- |
|  | Monognathic Model | Dignathic Model | Monognathic + Dignathic Model |
| **Four-age time-binning scheme** |  | | |
| Campanian | 0.06056318 | 0.05835967 | 0.04414708 |
| Maastrichtian | 0.06692364 | 0.07537542 | 0.06967781 |
| Danian-Selandian | 0.08260271 | 0.09095291 | 0.07513766 |
| Thanetian | 0.07279262 | 0.07416767 | 0.06513087 |

**Table EE.** Descriptive statistics of temporal distributions using the five-age time-binning scheme. IQR = Interquartile range.

|  | Skewness | | | | Kurtosis | | | | IQR | | | |
| --- | --- | --- | --- | --- | --- | --- | --- | --- | --- | --- | --- | --- |
|  | PC1 | PC2 | PC3 | PC4 | PC1 | PC2 | PC3 | PC4 | PC1 | PC2 | PC3 | PC4 |
| Campanian | 0.200 | -0.408 | 0.246 | -0.654 | 2.704 | 3.097 | 2.419 | 3.875 | 0.278 | 0.129 | 0.124 | 0.071 |
| Maastrichtian | 0.621 | -0.080 | 0.068 | -1.610 | 2.721 | 2.479 | 2.571 | 8.246 | 0.287 | 0.158 | 0.133 | 0.054 |
| Danian | 0.261 | 0.283 | 0.440 | -0.707 | 2.351 | 3.078 | 3.162 | 4.949 | 0.347 | 0.130 | 0.115 | 0.072 |
| Selandian | 0.133 | -0.052 | 1.070 | -0.030 | 2.448 | 2.999 | 2.857 | 2.876 | 0.185 | 0.103 | 0.049 | 0.056 |
| Thanetian | -0.033 | 0.318 | -0.056 | -0.731 | 2.245 | 2.815 | 3.330 | 3.499 | 0.352 | 0.114 | 0.133 | 0.075 |

**Table FF.** Descriptive statistics of temporal distributions using the four-age time-binning scheme. IQR = Interquartile range. IQR = Interquartile range; D-S = Danian-Selandian.

|  | Skewness | | | | Kurtosis | | | | IQR | | | |
| --- | --- | --- | --- | --- | --- | --- | --- | --- | --- | --- | --- | --- |
|  | PC1 | PC2 | PC3 | PC4 | PC1 | PC2 | PC3 | PC4 | PC1 | PC2 | PC3 | PC4 |
| Campanian | 0.200 | -0.408 | 0.246 | -0.654 | 2.704 | 3.097 | 2.419 | 3.875 | 0.278 | 0.129 | 0.124 | 0.071 |
| Maastrichtian | 0.621 | -0.080 | 0.068 | -1.610 | 2.721 | 2.479 | 2.571 | 8.246 | 0.287 | 0.158 | 0.133 | 0.054 |
| D-S | 0.245 | 0.224 | 0.350 | -0.724 | 2.303 | 2.989 | 2.880 | 4.982 | 0.361 | 0.133 | 0.124 | 0.074 |
| Thanetian | -0.033 | 0.318 | -0.056 | -0.731 | 2.245 | 2.815 | 3.330 | 3.499 | 0.352 | 0.114 | 0.133 | 0.075 |

**Table GG.** False discovery rate adjusted p-values obtained from multiple pairwise comparisons between time-bins. Reported statistics correspond to the morphospace analysis of Selachimorpha. Bold values are significant.

|  | Pairwise *P*-values between means (Pr > d) | | | | |
| --- | --- | --- | --- | --- | --- |
|  | PC’s (all) | PC1 | PC2 | PC3 | PC4 |
| **Five-age time-binning scheme** | _P_FDR | _P_FDR | _P_FDR | _P_FDR | _P_FDR |
| Campanian: Maastrichtian | **0.003** | **0.025** | 0.656 | **0.002** | 0.246 |
| Campanian: Danian | **0.008** | 0.576 | **0.008** | **0.002** | 0.578 |
| Campanian: Selandian | **0.010** | 0.434 | **0.009** | **0.002** | **0.042** |
| Campanian: Thanetian | **0.003** | 0.063 | **0.008** | **0.002** | 1.000 |
| Maastrichtian: Danian | **0.004** | **0.013** | **0.015** | **0.008** | 0.098 |
| Maastrichtian: Selandian | **0.003** | 1.000 | **0.010** | **0.002** | **0.042** |
| Maastrichtian: Thanetian | **0.003** | **0.013** | **0.008** | 0.074 | 0.504 |
| Danian: Selandian | **0.007** | 0.298 | 0.123 | **0.002** | 0.098 |
| Danian: Thanetian | 0.210 | 0.298 | 0.656 | 0.634 | 0.578 |
| Selandian: Thanetian | **0.003** | 0.100 | 0.209 | **0.002** | **0.042** |
| **Four-age time-binning scheme** |  | | | | |
| Campanian: Maastrichtian | **0.002** | **0.011** | 0.878 | **0.002** | 0.364 |
| Campanian: Danian-Selandian | **0.006** | 0.807 | **0.002** | **0.002** | 0.364 |
| Campanian: Thanetian | **0.002** | **0.040** | **0.002** | **0.002** | 1.000 |
| Maastrichtian: Danian-Selandian | **0.002** | **0.008** | **0.002** | **0.002** | **0.032** |
| Maastrichtian: Thanetian | **0.002** | **0.008** | **0.002** | 0.080 | 0.485 |
| Danian-Selandian: Thanetian | 0.069 | 0.123 | 0.999 | 0.139 | 0.364 |
| **Regional subsample** |  | | | | |
| late Maastrichtian: early Danian | 1.000 | 1.000 | 1 | 1.000 | 0.354 |
| late Maastrichtian: middle Danian | **0.002** | **0.005** | 1 | **0.007** | 0.167 |
| early Danian: middle Danian | **0.002** | **0.005** | 1 | **0.007** | 0.354 |

**Table HH.** False discovery rate adjusted p-values obtained from multiple pairwise comparisons between time-bins. Reported statistics correspond to the temporal morphospace analysis of Galeomorphii (including †Synechodontiformes). Bold values are significant.

|  | Pairwise *P*-values between means (Pr > d) | | | | |
| --- | --- | --- | --- | --- | --- |
| **Four-age time-binning scheme** | PC’s (all) | PC1 | PC2 | PC3 | PC4 |
| Campanian: Maastrichtian | **0.001** | 0.204 | **0.034** | **0.001** | **0.010** |
| Campanian: Danian-Selandian | **0.001** | **0.001** | **0.001** | 0.066 | **0.033** |
| Campanian: Thanetian | **0.002** | **0.005** | **0.002** | **0.002** | 0.951 |
| Maastrichtian: Danian-Selandian | **0.001** | **0.001** | **0.026** | **0.001** | **0.001** |
| Maastrichtian: Thanetian | **0.001** | **0.001** | 0.114 | **0.004** | **0.037** |
| Danian-Selandian: Thanetian | 0.269 | 0.441 | 0.595 | 0.084 | 0.066 |
| **Stevns Klint regional sub-sample** |  | | | | |
| late Maastrichtian: early Danian | 0.475 | 0.520 | 0.691 | 0.120 | 0.342 |
| late Maastrichtian: middle Danian | **0.007** | **0.019** | 0.636 | **0.001** | 0.060 |
| early Danian: middle Danian | **0.002** | **0.002** | 0.376 | **0.008** | 0.232 |

**Table II.** False discovery rate adjusted p-values obtained from multiple pairwise comparisons between time-bins. Reported statistics correspond to the morphospace analysis of Squalomorphii. Bold values are significant.

|  | Pairwise P-values between means (Pr > d) | | | | |
| --- | --- | --- | --- | --- | --- |
| **Four-age time-binning scheme** | PC’s (all) | PC1 | PC2 | PC3 | PC4 |
| Campanian: Maastrichtian | **0.004** | **0.048** | 0.083 | 1.000 | 0.168 |
| Campanian: Danian-Selandian | 0.211 | 0.240 | 1.000 | 0.339 | 0.376 |
| Campanian: Thanetian | **0.014** | 0.093 | 0.136 | 0.088 | 1.000 |
| Maastrichtian: Danian-Selandian | **0.005** | 0.093 | **0.016** | 0.184 | 0.389 |
| Maastrichtian: Thanetian | **0.004** | 0.931 | **0.008** | **0.016** | 0.172 |
| Danian-Selandian: Thanetian | 0.082 | 0.462 | 0.084 | 0.212 | 0.389 |

**Supplementary Table JJ.** PC1–PC4 pairwise comparisons between time-bins for Lamniformes (N=660) using the four-age time-binning scheme. Raw and FDR-adjusted P-values are provided. Bold values indicate significant difference between treatment means at α=0.05.

|  | Pairwise *P*-values between means (Pr > d) | | | | | | | | | |
| --- | --- | --- | --- | --- | --- | --- | --- | --- | --- | --- |
| **Treatments** | PC’s (all) | | PC1 | | PC2 | | PC3 | | PC4 | |
|  | *P* | FDR | *P* | FDR | *P* | FDR | *P* | FDR | *P* | FDR |
| C:M | **0.008** | **0.013** | 0.751 | 1.000 | 0.157 | 0.251 | **0.001** | **0.002** | **0.001** | **0.008** |
| C: D-S | **0.001** | **0.002** | **0.001** | **0.002** | **0.001** | **0.004** | 0.249 | 0.398 | 0.331 | 0.530 |
| C: T | **0.001** | **0.002** | **0.001** | **0.002** | **0.031** | 0.062 | **0.001** | **0.002** | **0.003** | **0.012** |
| M: D-S | **0.001** | **0.002** | **0.001** | **0.002** | **0.001** | **0.004** | **0.001** | **0.002** | **0.009** | **0.024** |
| M: T | **0.001** | **0.002** | **0.001** | **0.002** | 0.280 | 0.373 | 0.335 | 0.447 | 0.693 | 0.924 |
| D-S: T | 0.054 | 0.072 | 0.558 | 0.893 | **0.029** | 0.062 | **0.001** | **0.002** | 0.078 | 0.156 |

C = Campanian, M = Maastrichtian, D-S = Danian-Selandian, T = Thanetian.

**Table KK.** PC1–PC4 pairwise comparisons between time-bins for Carcharhiniformes (N=228) using the four-age time-binning scheme. Raw and FDR-adjusted P-values are provided. Bold values indicate significant difference between treatment means at α=0.05.

|  | Pairwise *P*-values between means (Pr > d) | | | | | | | | | |
| --- | --- | --- | --- | --- | --- | --- | --- | --- | --- | --- |
| **Treatments** | PC’s (all) | | PC1 | | PC2 | | PC3 | | PC4 | |
|  | *P* | FDR | *P* | FDR | *P* | FDR | *P* | FDR | *P* | FDR |
| C:M | 0.067 | 0.134 | 0.095 | 0.253 | 0.154 | 0.308 | 0.091 | 0.243 | 0.243 | 0.616 |
| C: D-S | **0.002** | **0.016** | **0.003** | **0.024** | **0.031** | 0.124 | 0.810 | 1.000 | 0.308 | 0.616 |
| C: T | 0.054 | 0.134 | 0.284 | 0.509 | **0.007** | 0.056 | 0.391 | 0.243 | 0.524 | 0.838 |
| M: D-S | 0.140 | 0.187 | 0.318 | 0.509 | 0.607 | 0.809 | 0.083 | 0.243 | **0.012** | 0.096 |
| M: T | 0.119 | 0.187 | 0.407 | 0.543 | 0.122 | 0.308 | **0.009** | 0.072 | **0.047** | 0.188 |
| D-S: T | **0.019** | 0.076 | **0.041** | 0.164 | 0.216 | 0.346 | 0.198 | 0.396 | 0.716 | 0.955 |

C = Campanian, M = Maastrichtian, D-S = Danian-Selandian, T = Thanetian.

**Table LL.** PC1–PC4 pairwise comparisons between time-bins for Heterodontiformes (N=23) using the four-age time-binning scheme. Raw and FDR-adjusted P-values are provided. Bold values indicate significant difference between treatment means at α=0.05.

|  | Pairwise *P*-values between means (Pr > d) | | | | | | | | | |
| --- | --- | --- | --- | --- | --- | --- | --- | --- | --- | --- |
| **Treatments** | PC’s (all) | | PC1 | | PC2 | | PC3 | | PC4 | |
|  | *P* | FDR | *P* | FDR | *P* | FDR | *P* | FDR | *P* | FDR |
| C:M | 0.315 | 1 | 0.335 | 1 | 0.186 | 0.744 | 0.334 | 1 | 0.299 | 1 |
| C: D-S | 0.545 | 1 | 0.640 | 1 | 0.073 | 0.584 | 0.756 | 1 | 0.501 | 1 |
| C: T | 0.838 | 1 | 0.794 | 1 | 0.375 | 0.774 | 0.824 | 1 | 0.758 | 1 |
| M: D-S | 0.856 | 1 | 0.722 | 1 | 0.559 | 0.894 | 0.612 | 1 | 0.788 | 1 |
| M: T | 0.581 | 1 | 0.474 | 1 | 0.735 | 0.980 | 0.434 | 1 | 0.475 | 1 |
| D-S: T | 0.906 | 1 | 0.824 | 1 | 0.387 | 0.774 | 0.876 | 1 | 0.730 | 1 |

C = Campanian, M = Maastrichtian, D-S = Danian-Selandian, T = Thanetian.

**Table MM.** PC1–PC4 pairwise comparisons between time-bins for Orectolobiformes (N=116) using the four-age time-binning scheme. Raw and FDR-adjusted P-values are provided. Bold values indicate significant difference between treatment means at α=0.05.

|  | Pairwise *P*-values between means (Pr > d) | | | | | | | | | |
| --- | --- | --- | --- | --- | --- | --- | --- | --- | --- | --- |
| **Treatments** | PC’s (all) | | PC1 | | PC2 | | PC3 | | PC4 | |
|  | *P* | FDR | *P* | FDR | *P* | FDR | *P* | FDR | *P* | FDR |
| C:M | 0.156 | 0.250 | 0.670 | 1.000 | **0.043** | 0.115 | 0.081 | 0.216 | **0.036** | 0.072 |
| C: D-S | **0.010** | 0.080 | 0.083 | 0.221 | **0.028** | 0.112 | **0.011** | 0.088 | **0.001** | **0.008** |
| C: T | 0.065 | 0.173 | 0.908 | 1.000 | **0.013** | 0.104 | **0.031** | 0.124 | **0.006** | **0.024** |
| M: D-S | **0.048** | 0.173 | 0.077 | 0.221 | 0.559 | 0.894 | 0.154 | 0.308 | **0.018** | **0.048** |
| M: T | 0.500 | 0.667 | 0.605 | 1.000 | 0.338 | 0.676 | 0.261 | 0.418 | 0.173 | 0.277 |
| D-S: T | 0.105 | 0.210 | 0.080 | 0.221 | 0.710 | 0.947 | 0.945 | 1.000 | 0.547 | 0.729 |

C = Campanian, M = Maastrichtian, D-S = Danian-Selandian, T = Thanetian.

**Table NN.** PC1–PC4 pairwise comparisons between time-bins for Hexanchiformes (N=40) using the four-age time-binning scheme. Raw and FDR-adjusted P-values are provided. Bold values indicate significant difference between treatment means at α=0.05.

|  | Pairwise *P*-values between means (Pr > d) | | | | | | | | | |
| --- | --- | --- | --- | --- | --- | --- | --- | --- | --- | --- |
| **Treatments** | PC’s (all) | | PC1 | | PC2 | | PC3 | | PC4 | |
|  | *P* | FDR | *P* | FDR | *P* | FDR | *P* | FDR | *P* | FDR |
| M: D-S | **0.047** | 0.212 | 0.090 | 0.405 | 0.436 | 0.981 | **0.011** | 0.050 | 0.056 | 0.252 |
| M: T | 0.201 | 0.452 | 0.219 | 0.493 | 0.329 | 0.981 | 0.141 | 0.291 | 0.203 | 0.457 |
| D-S: T | 0.559 | 0.838 | 0.621 | 0.932 | 0.729 | 1.000 | 0.194 | 0.291 | 0.473 | 0.710 |

M = Maastrichtian, D-S = Danian-Selandian, T = Thanetian.

**Table OO.** PC1–PC4 pairwise comparisons between time-bins for Squaliformes (N=86) using the four-age time-binning scheme. Raw and FDR-adjusted P-values are provided. Bold values indicate significant difference between treatment means at α=0.05.

|  | Pairwise *P*-values between means (Pr > d) | | | | | | | | | |
| --- | --- | --- | --- | --- | --- | --- | --- | --- | --- | --- |
| **Treatments** | PC’s (all) | | PC1 | | PC2 | | PC3 | | PC4 | |
|  | *P* | FDR | *P* | FDR | *P* | FDR | *P* | FDR | *P* | FDR |
| C:M | **0.030** | 0.120 | 0.055 | 0.220 | 0.102 | 0.816 | 0.758 | 1.000 | 0.054 | 0.216 |
| C: D-S | 0.321 | 0.856 | 0.824 | 1.000 | 0.300 | 0.982 | 0.097 | 0.388 | 0.863 | 1.000 |
| C: T | 0.681 | 1.000 | 0.347 | 0.778 | 0.925 | 1.000 | 0.618 | 1.000 | 0.643 | 1.000 |
| M: D-S | **0.013** | 0.104 | **0.019** | 0.152 | 0.491 | 0.982 | **0.046** | 0.368 | **0.016** | 0.128 |
| M: T | 0.771 | 1.000 | 0.953 | 1.000 | 0.412 | 0.982 | 0.747 | 1.000 | 0.127 | 0.339 |
| D-S: T | 0.764 | 1.000 | 0.389 | 0.778 | 0.629 | 1.000 | 0.630 | 1.000 | 0.526 | 1.000 |

C = Campanian, M = Maastrichtian, D-S = Danian-Selandian, T = Thanetian.

**Table PP.** PC1–PC4 pairwise comparisons between time-bins for Squatiniformes (N=86) using the four-age time-binning scheme. Raw and FDR-adjusted P-values are provided. Bold values indicate significant difference between treatment means at α=0.05.

|  | Pairwise *P*-values between means (Pr > d) | | | | | | | | | |
| --- | --- | --- | --- | --- | --- | --- | --- | --- | --- | --- |
|  | PC’s (all) | | PC1 | | PC2 | | PC3 | | PC4 | |
|  | *P* | FDR | *P* | FDR | *P* | FDR | *P* | FDR | *P* | FDR |
| C: M | 0.115 | 0.518 | 0.394 | 0.887 | **0.022** | 0.099 | 0.726 | 1.000 | 0.185 | 0.711 |
| C: D-S | 0.395 | 0.592 | 0.859 | 1.000 | 0.241 | 0.542 | **0.030** | 0.135 | 0.878 | 1.000 |
| M: D-S | 0.388 | 0.592 | 0.363 | 0.887 | 0.369 | 0.554 | 0.153 | 0.344 | 0.316 | 0.711 |

C = Campanian, M = Maastrichtian, D-S = Danian-Selandian.

**Table QQ.** PC1–PC4 pairwise comparisons between time-bins for †Synechodontiformes (N=16) using the four-age time-binning scheme. Raw and FDR-adjusted P-values are provided. Bold values indicate significant difference between treatment means at α=0.05.

|  | Pairwise *P*-values between means (Pr > d) | | | | | | | | | |
| --- | --- | --- | --- | --- | --- | --- | --- | --- | --- | --- |
|  | PC’s (all) | | PC1 | | PC2 | | PC3 | | PC4 | |
|  | *P* | FDR | *P* | FDR | *P* | FDR | *P* | FDR | *P* | FDR |
| C: M | 0.602 | 1 | 0.506 | 1 | 0.966 | 1.000 | 0.857 | 1 | 0.857 | 1 |
| C: D-S | 0.373 | 1 | 0.346 | 1 | 0.295 | 0.767 | 0.664 | 1 | 0.986 | 1 |
| M: D-S | 0.898 | 1 | 0.952 | 1 | 0.341 | 0.767 | 0.788 | 1 | 0.794 | 1 |

C = Campanian, M = Maastrichtian, D-S = Danian-Selandian.

**Table RR.** Lagerstätten effects on disparity in Selachimorpha (N=1045) using the four-age time-binning scheme. PV and pairwise statistics are provided. Upper off-diagonal cells depict FDR-adjusted P-values; lower off-diagonal cells depict raw P-values. Bold values indicate significant difference between treatment means at α=0.05.

|  | Raw Disparity | Pairwise comparisons among time-bins | | | |
| --- | --- | --- | --- | --- | --- |
| **Four-age time-binning scheme** |  | Campanian | Maastrichtian | D-S | Thanetian |
| Campanian | 0.06911023 | - | 0.547 | 0.181 | **0.040** |
| Maastrichtian | 0.07331670 | 0.397 | - | 0.547 | 0.181 |
| D-S | 0.07980210 | 0.068 | 0.284 | - | 0.547 |
| Thanetian | 0.08535443 | **0.005** | **0.047** | 0.410 | - |

D-S = Danian-Selandian.

**Table SS.** False discovery rate adjusted p-values obtained from multiple pairwise comparisons between time-bins. Sensitivity analysis excluding the Stevns Klint regional sub-sample (N=1045) and a four-age time-binning scheme.

|  | Pairwise *P*-values between means (Pr > d) | | | | |
| --- | --- | --- | --- | --- | --- |
|  | PC’s (all) | PC1 | PC2 | PC3 | PC4 |
| **Four-age time-binning scheme** | _P_FDR | _P_FDR | _P_FDR | _P_FDR | _P_FDR |
| Maastrichtian: Danian-Selandian | **0.002** | **0.004** | **0.004** | **0.002** | 0.632 |

**Table TT.** Non-parametric multivariate analysis of variance with RRPP testing of monognathic heterodonty on morphospace(N=932). Coefficient estimation via ordinary least squares (OLS). Type I (sequential) sums of squares were used to calculate sums of squares and cross-products matrices. Effect sizes (Z) are based on F distribution.

|  | *d.f.* | SS | MS | R^2^ | F | Z | Pr(>F) |
| --- | --- | --- | --- | --- | --- | --- | --- |
| **All PC-axes** |  |  |  |  |  |  |  |
| ~ tooth positions | 3 | 11.066 | 3.6886 | 0.14037 | 50.511 | 7.4612 | 0.001** |
| **PC1** |  |  |  |  |  |  |  |
| ~ tooth positions | 3 | 10.078 | 3.3592 | 0.20297 | 78.772 | 4.7538 | 0.001** |
| **PC2** |  |  |  |  |  |  |  |
| ~ tooth positions | 3 | 0.0640 | 0.021337 | 0.00666 | 2.0734 | 1.1592 | 0.097 |
| **PC3** |  |  |  |  |  |  |  |
| ~ tooth positions | 3 | 0.7135 | 0.23783 | 0.08788 | 29.804 | 3.7889 | 0.001** |
| **PC4** |  |  |  |  |  |  |  |
| ~ tooth positions | 3 | 0.1619 | 0.053969 | 0.04492 | 14.548 | 3.2897 | 0.001** |

Abbreviations: *d*.*f*. = degrees of freedom; SS = sequential sums of squares; MS = Mean squares; F statistics = F value by permutation; R^2^ = coefficient of determination; Z = effect size. *P*-values are based on 999 permutations.

**Table UU.** PC1–PC4 pairwise comparisons sensitivity analysis of monognathic heterodonty. Raw and FDR-adjusted P-values are provided. Bold values indicate significant difference between treatment means at α=0.05.

|  | Pairwise *P*-values between means (Pr > d) | | | | | | | |
| --- | --- | --- | --- | --- | --- | --- | --- | --- |
| **Tooth positions** | PC1 | | PC2 | | PC3 | | PC4 | |
|  | *P* | FDR | *P* | FDR | *P* | FDR | *P* | FDR |
| parasymphyseal: anterior | 0.838 | 1.000 | 0.906 | 1.000 | 0.823 | 1.000 | **0.005** | **0.010** |
| parasymphyseal: lateroposterior | **0.002** | **0.003** | 0.718 | 1.000 | **0.002** | **0.005** | **0.001** | **0.004** |
| parasymphyseal: posterior | **0.001** | **0.002** | 0.107 | 0.285 | **0.036** | 0.072 | **0.005** | **0.010** |
| anterior: lateroposterior | **0.001** | **0.002** | 0.524 | 1.000 | **0.001** | **0.005** | **0.001** | **0.004** |
| anterior: posterior | **0.001** | **0.002** | **0.011** | 0.088 | **0.002** | **0.005** | 0.298 | 0.397 |
| lateroposterior: posterior | **0.001** | **0.002** | **0.030** | 0.12 | 0.390 | 0.624 | 0.134 | 0.214 |

**Table VV.** PC1–PC4 pairwise comparisons sensitivity analysis of dignathic heterodonty. Raw and FDR-adjusted P-values are provided. Bold values indicate significant difference between treatment means at α=0.05.

|  | Pairwise *P*-values between means (Pr > d) | | | | | | | |
| --- | --- | --- | --- | --- | --- | --- | --- | --- |
| **Dental units** | PC1 | | PC2 | | PC3 | | PC4 | |
|  | *P* | FDR | *P* | FDR | *P* | FDR | *P* | FDR |
| lower: upper | 0.329 | 0.658 | 0.001 | 0.002 | 0.001 | 0.002 | 0.28 | 0.56 |

**Supplementary Figures**


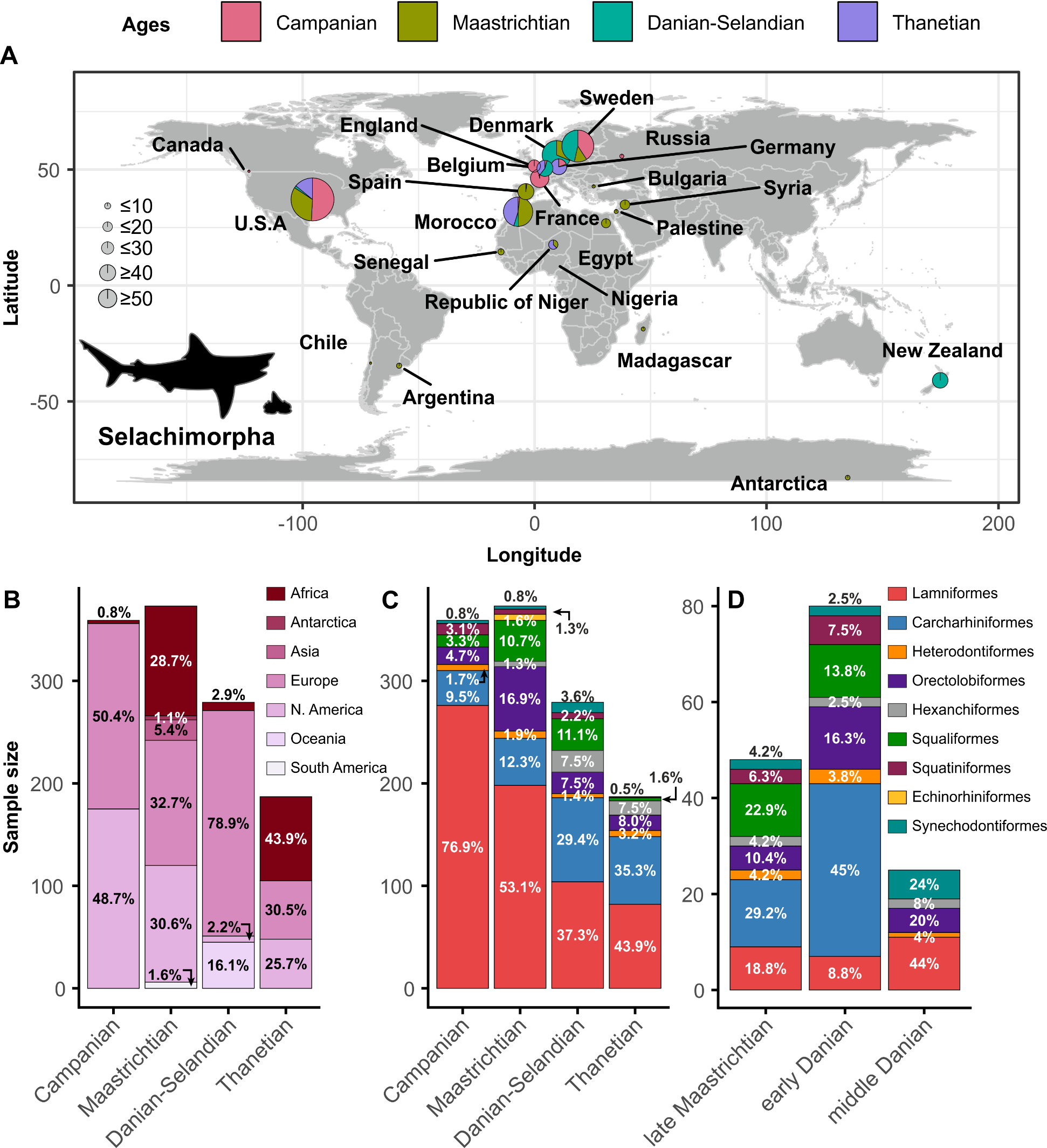


**Figure A.** **Geographic distribution, sample percentages and proportions in our global Selachimorpha dataset. (A)** Pie charts depict sample provenance and chronostratigraphic age. **(B)** Sample counts and percentages for each geographic region. **(C–D)** Order-level clade counts and percentages for each global four-age time-bin and region. Map was generated using the *maps* [9] and *ggplot2* [10] packages. Silhouettes generated by M.B. <https://cran.r-project.org/web/packages/maps/maps.pdf>


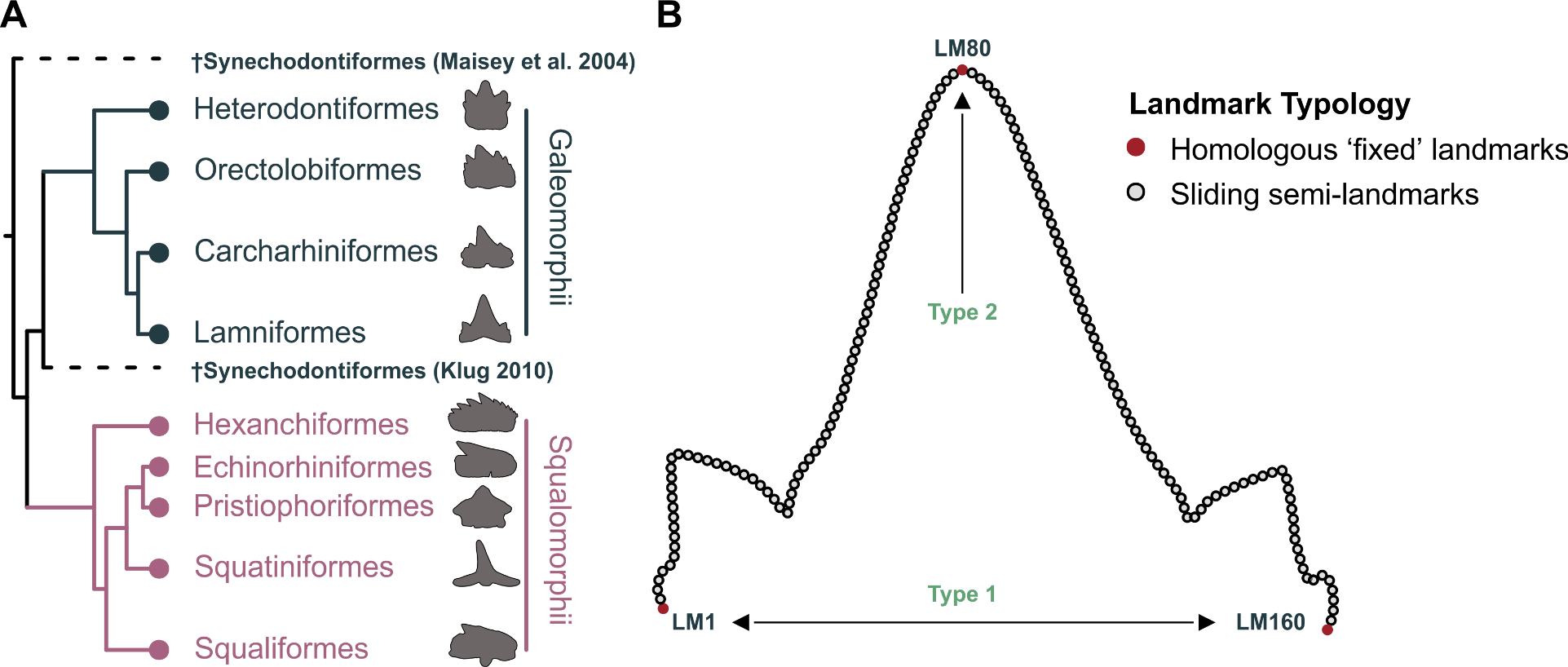


**Figure B.** **Phylogenetic framework and landmark scheme.** **(A)** Phylogenetic topology illustrating our order-level clade taxonomy. **(B)** Geometric morphometric landmark-scheme showing fixed type 1 and type 2 landmarks (red) versus sliding semi-landmarks (open circles). LM=landmarks. Silhouettes generated by M.B.


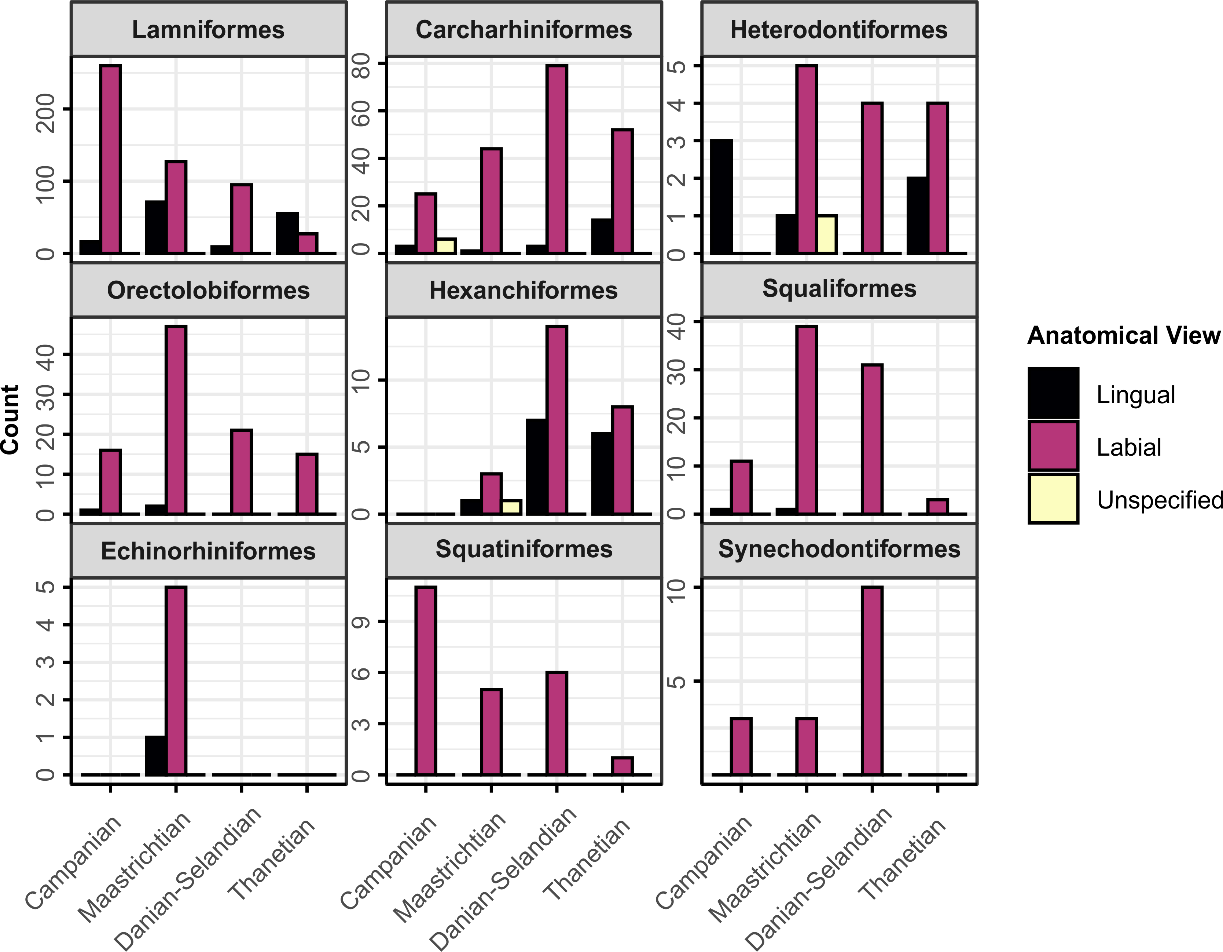


**Figure C. Bar plots of labial and lingual tooth views.** Based on the four-age time-binning scheme.


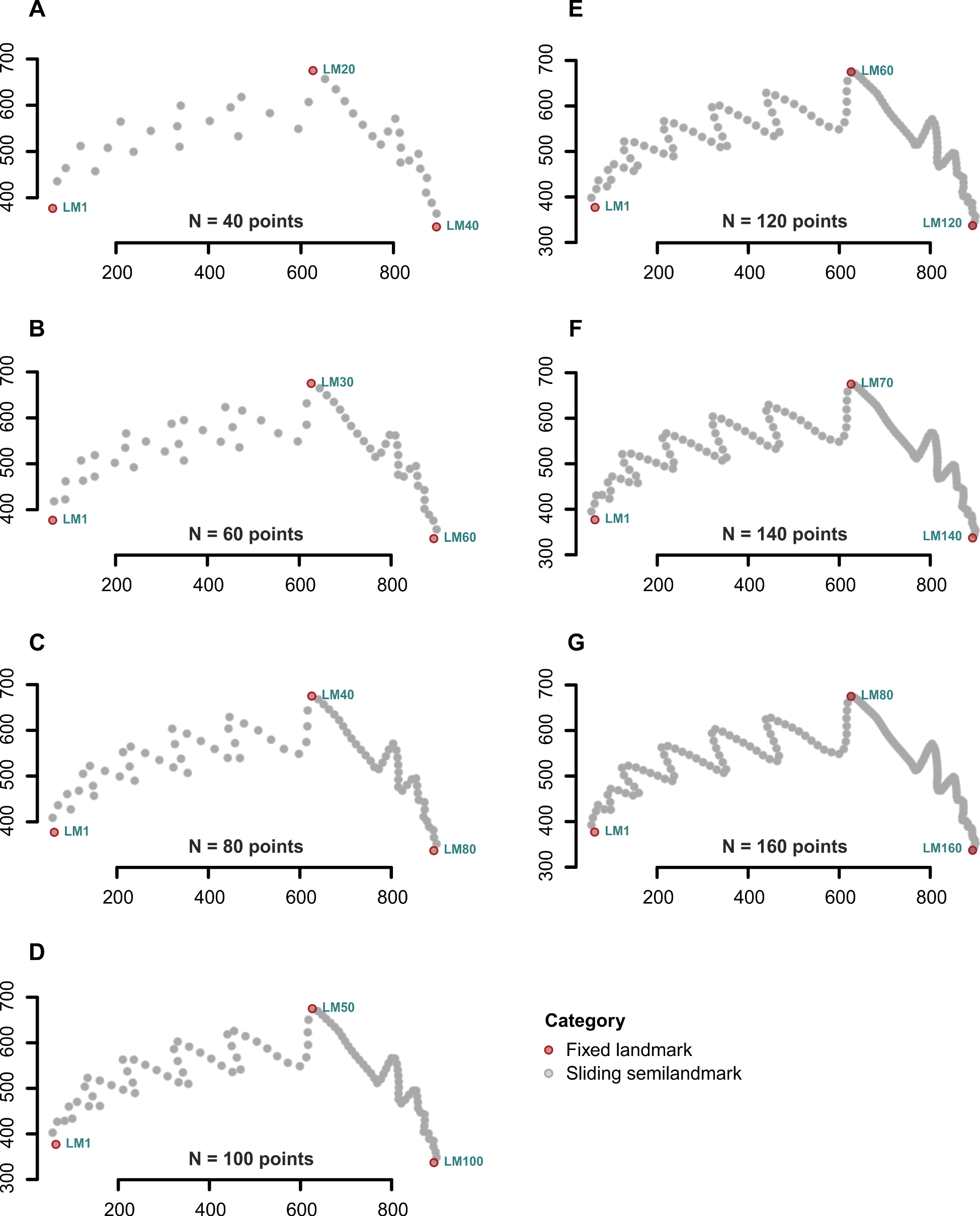


**Figure D. Resampled and scaled points.** Outlines show increasing point numbers up to the optimal 160 points (curve_1_=79, curve_2_=81). Curve end-points (LM1, LM80, and LM160) were treated as geometrically homologous landmarks.


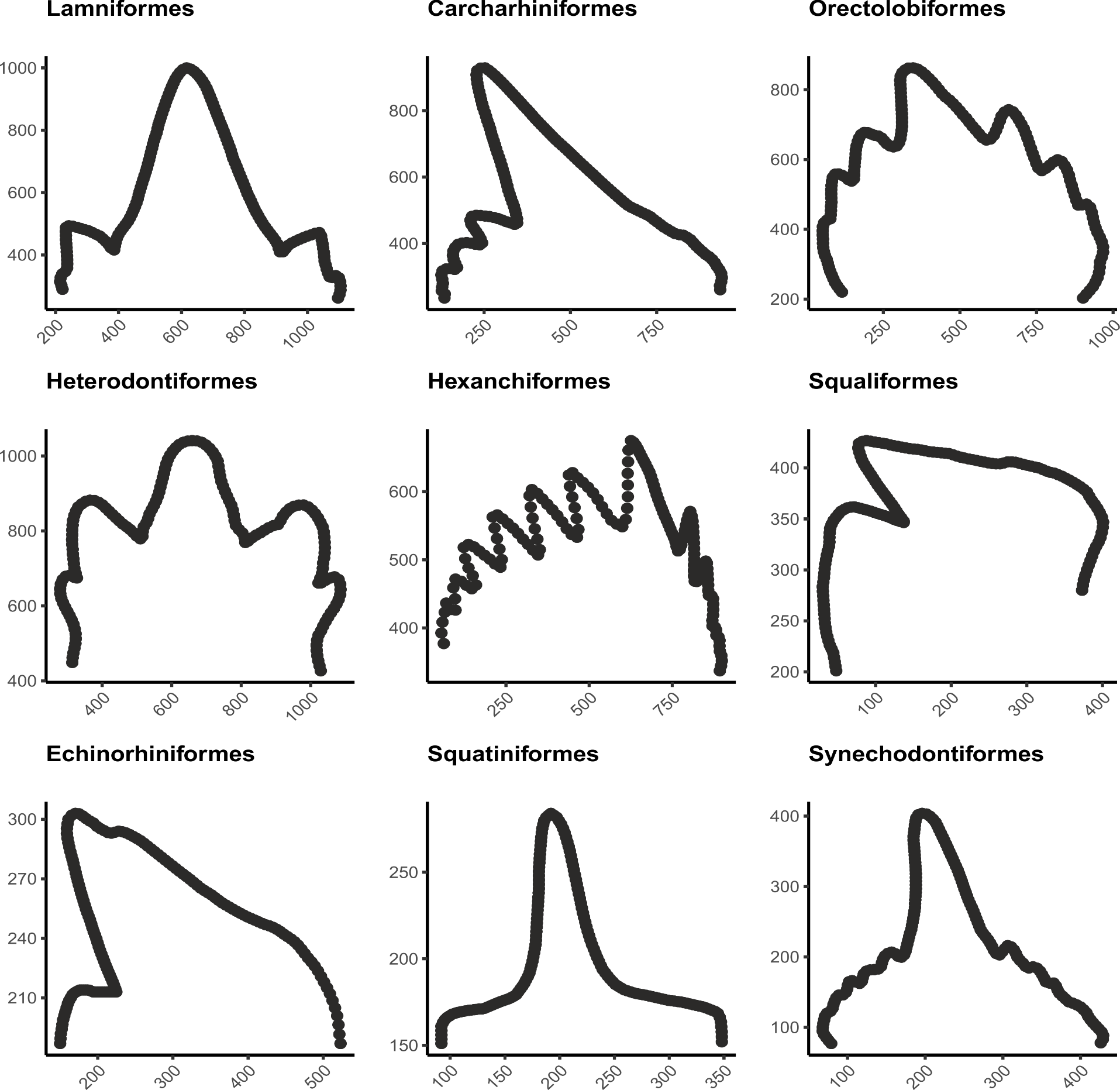


**Figure E.** **Landmark visualizations**. Lamniformes (Cretalamna maroccana); Carcharhiniformes (Danogaleus gueriri); Orectolobiformes (Plicatoscyllium lehneri); Heterodontiformes (Heterodontus granti); Hexanchiformes (Notidanodon dentatus); Squaliformes (Proetmopterus hemmooriensis); Echinorhiniformes (Echinorhinus maremagnum); Squatiniformes (Squatina sp); †Synechodontiformes (Synechodus filipi). Aspect ratio=1.


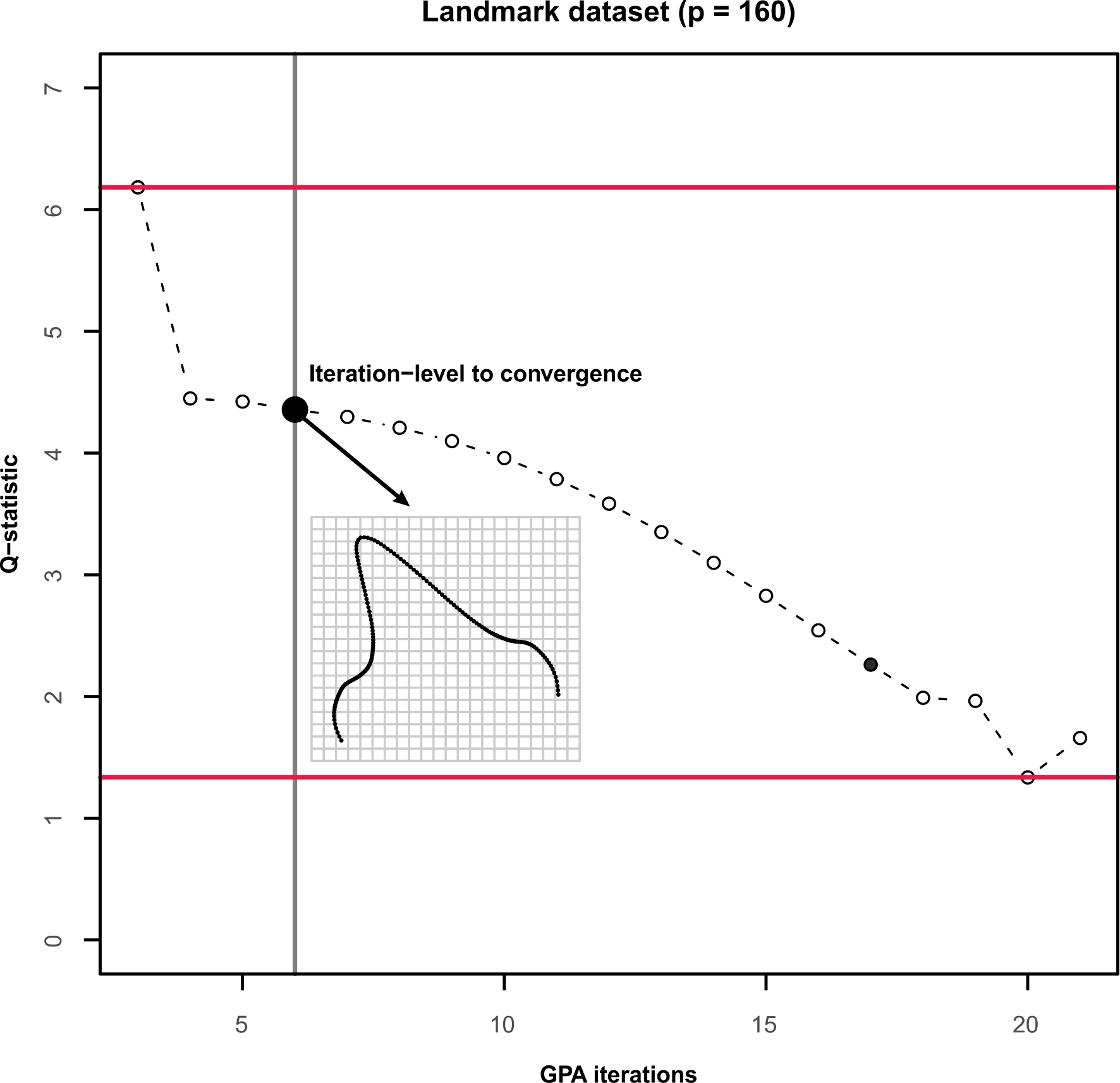


**Figure F.** **Sliding performance of semi-landmarks under different iterations**. Convergence required only 6 iterations. Consensus configurations from iterations >6 suffered from drift indicating no improvement in alignment solution. Red lines indicate maximum and minimum Q-values.


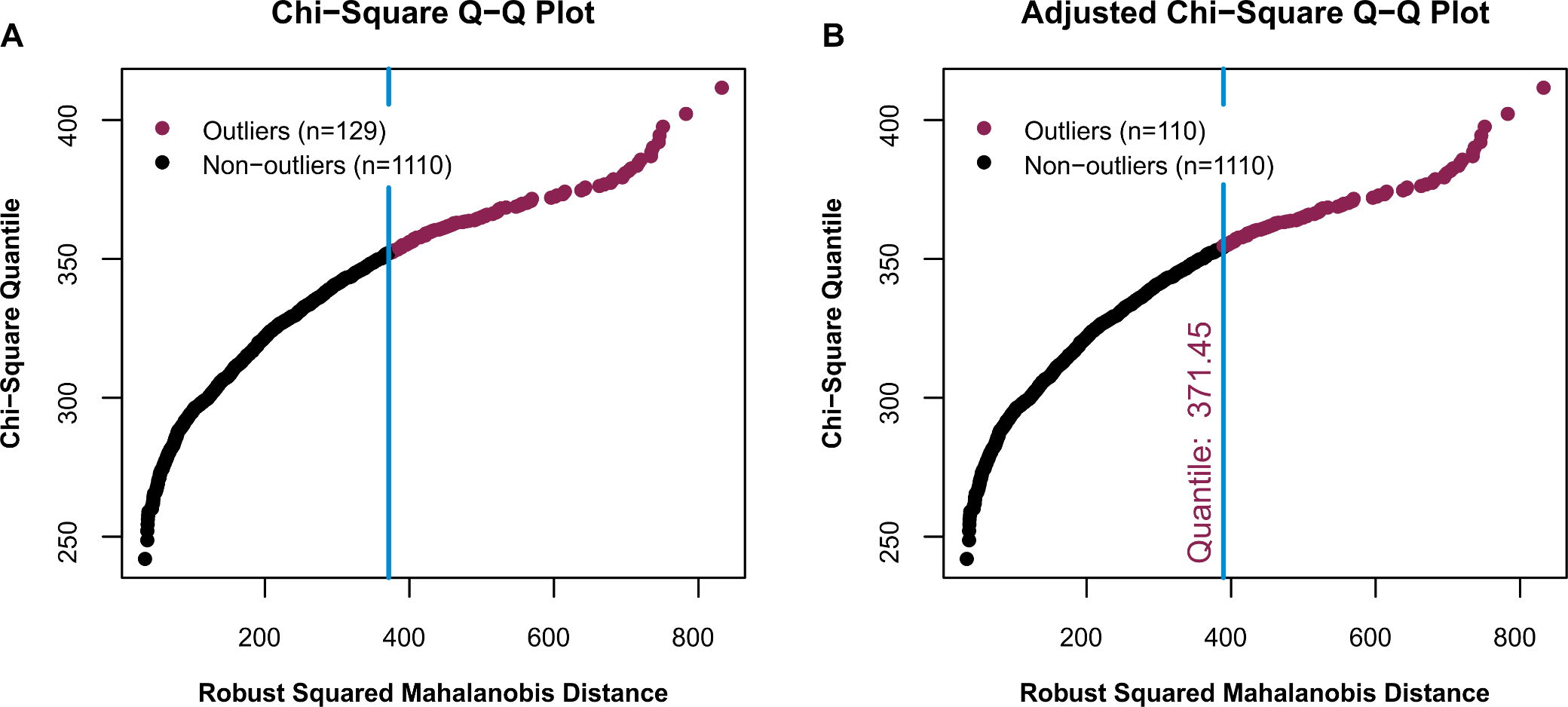


**Figure G. Henze-Zirkler's multivariate normality test of Procrustes-aligned coordinates**. **(A)** Chi-square and **(B)** adjusted chi-square Q-Q plot for multivariate outliers based on Mahalanobis distance.


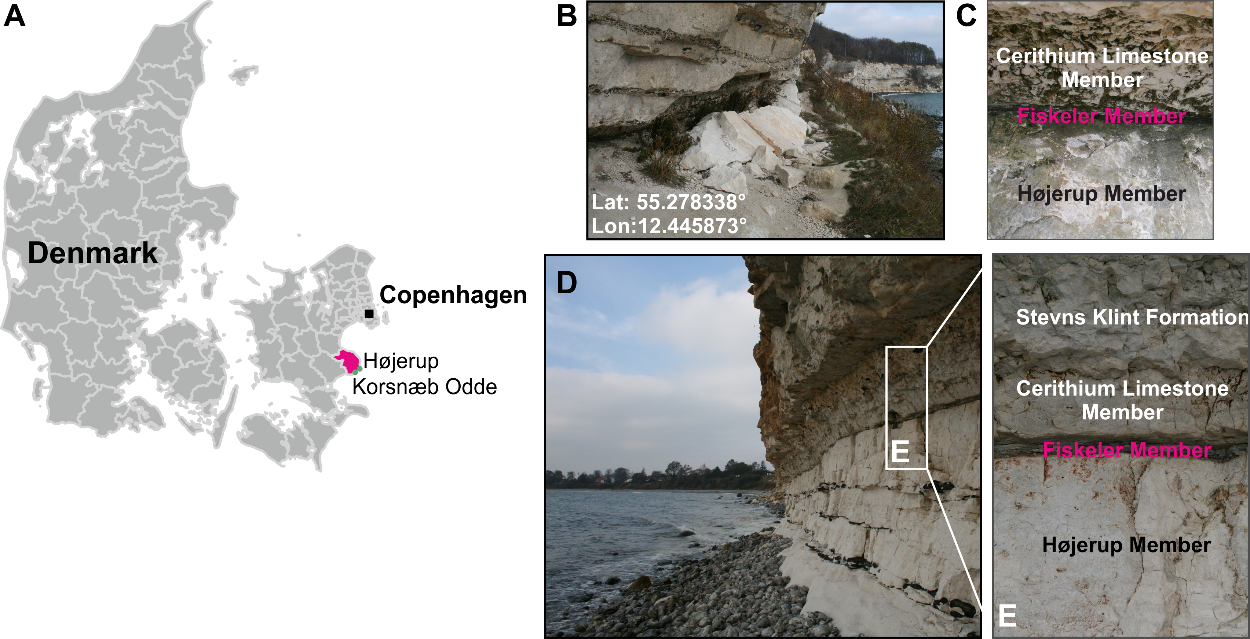


**Figure H. Locality map and lithostratigraphical units spanning the Maastrichtian–Danian boundary at Stevns Klint in Denmark. (A)** Map of Denmark and the municipality of Stevns in Sjælland. **(B–C)** Højerup. **(D–E)** Korsnæb Odde, near Rødvig. Map was generated using the *mapDK* package [11]. Images **(B–E)** courtesy Jesper Milan (Geomuseum Faxe). https://github.com/sebastianbarfort/mapDK


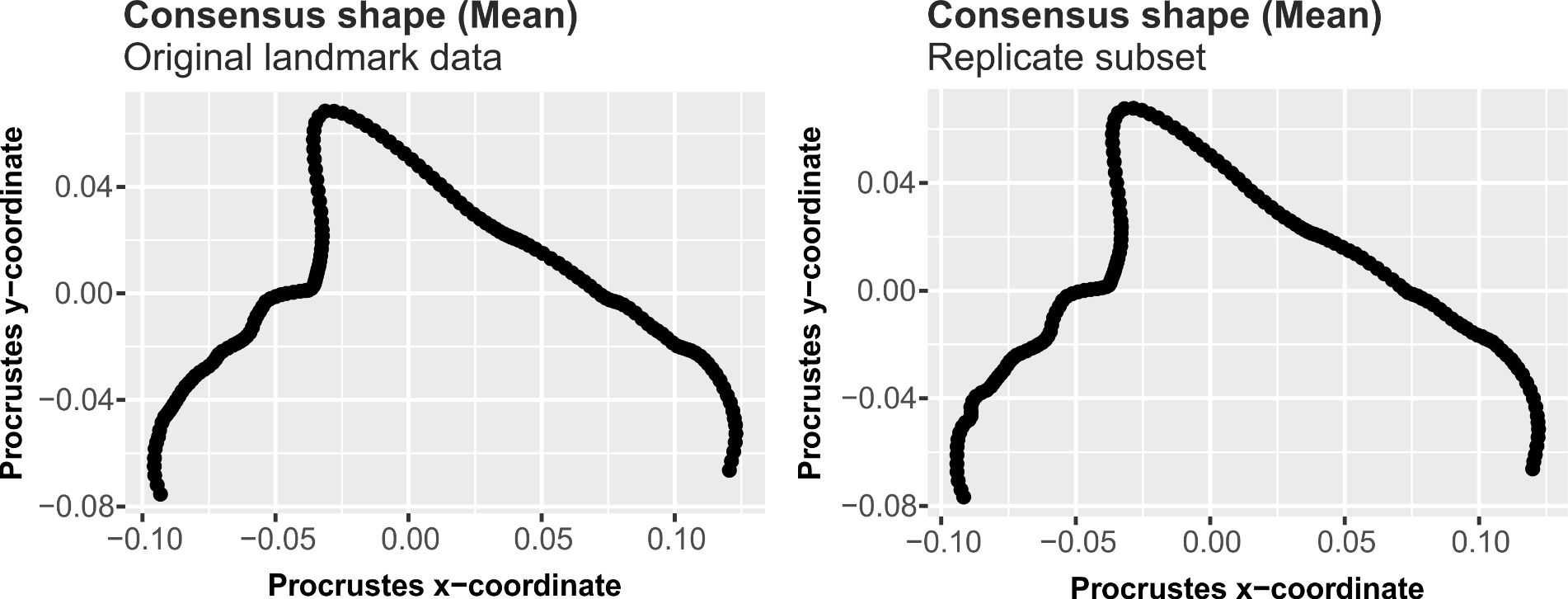


**Figure I.** **Mean shape comparisons from the first and second digitization (N=30).** Specimen information is given in Table G.


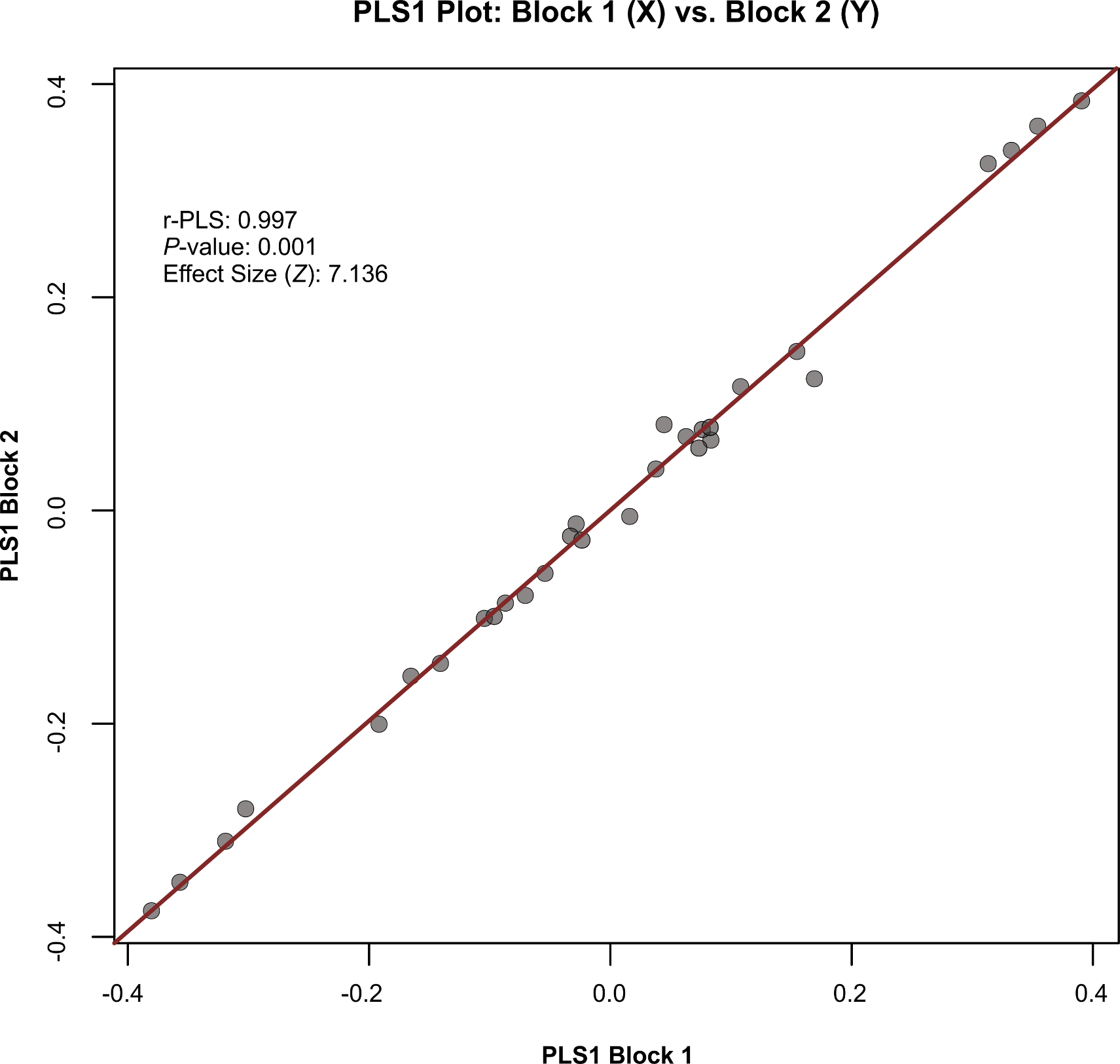


**Figure J. Two-block partial least squares (2B-PLS) analysis.** Results indicate strong association between digitization’s, and thus, minimal measurement error.


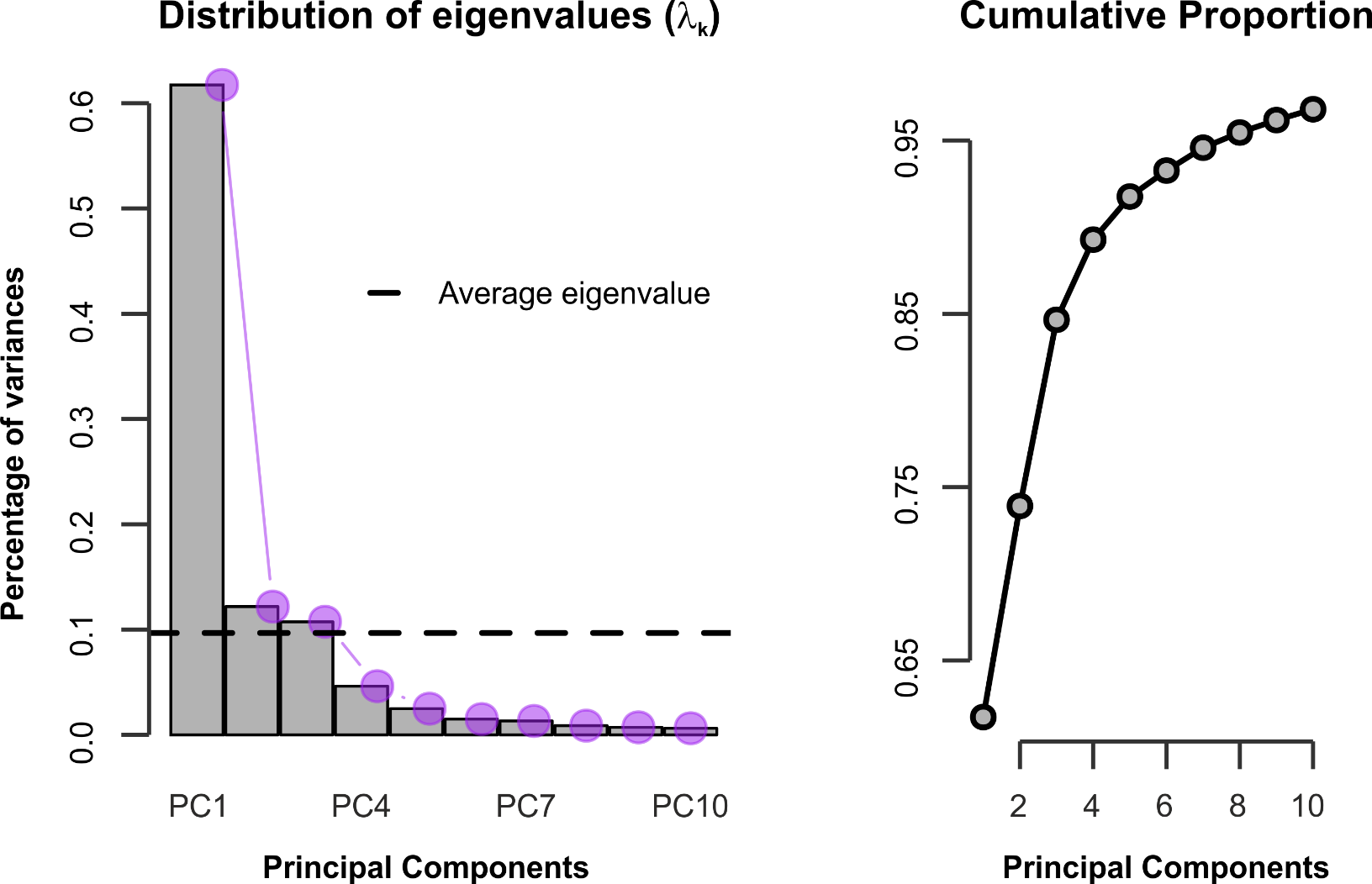


**Figure K.** **Summary statistics from principal component analysis.** Proportion of variance by component (PC1–PC10) and cumulative proportion of variance.

**
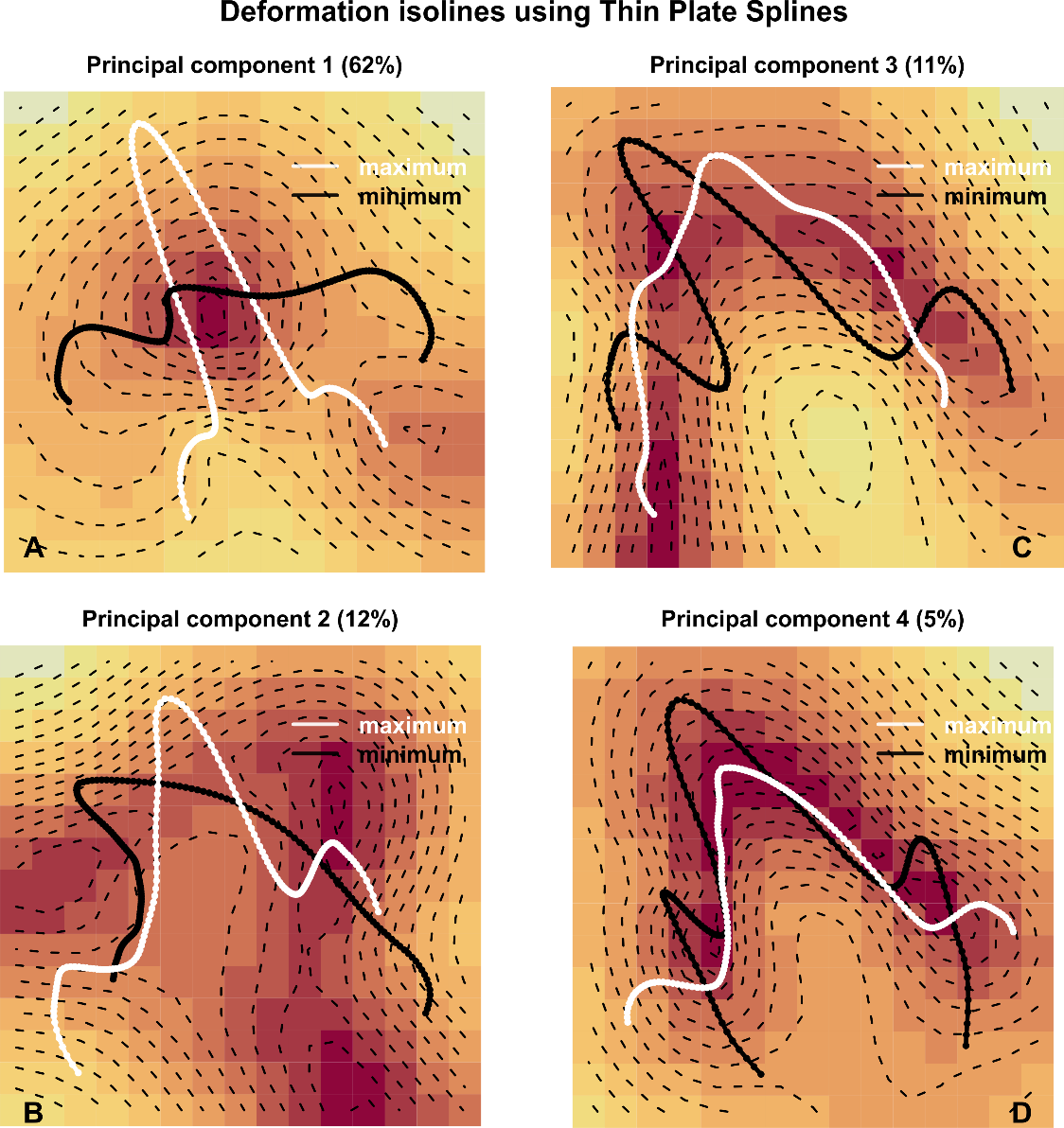
**

**Figure L. Hypothetical morphologies corresponding to the minimum and maximum values of PC1–PC4. (A–D)** Isolines and counter lines based on the TPS-method. Areas of concentrated strain are indicated by increasing red intensity and represents the amount of change at any given location [3].


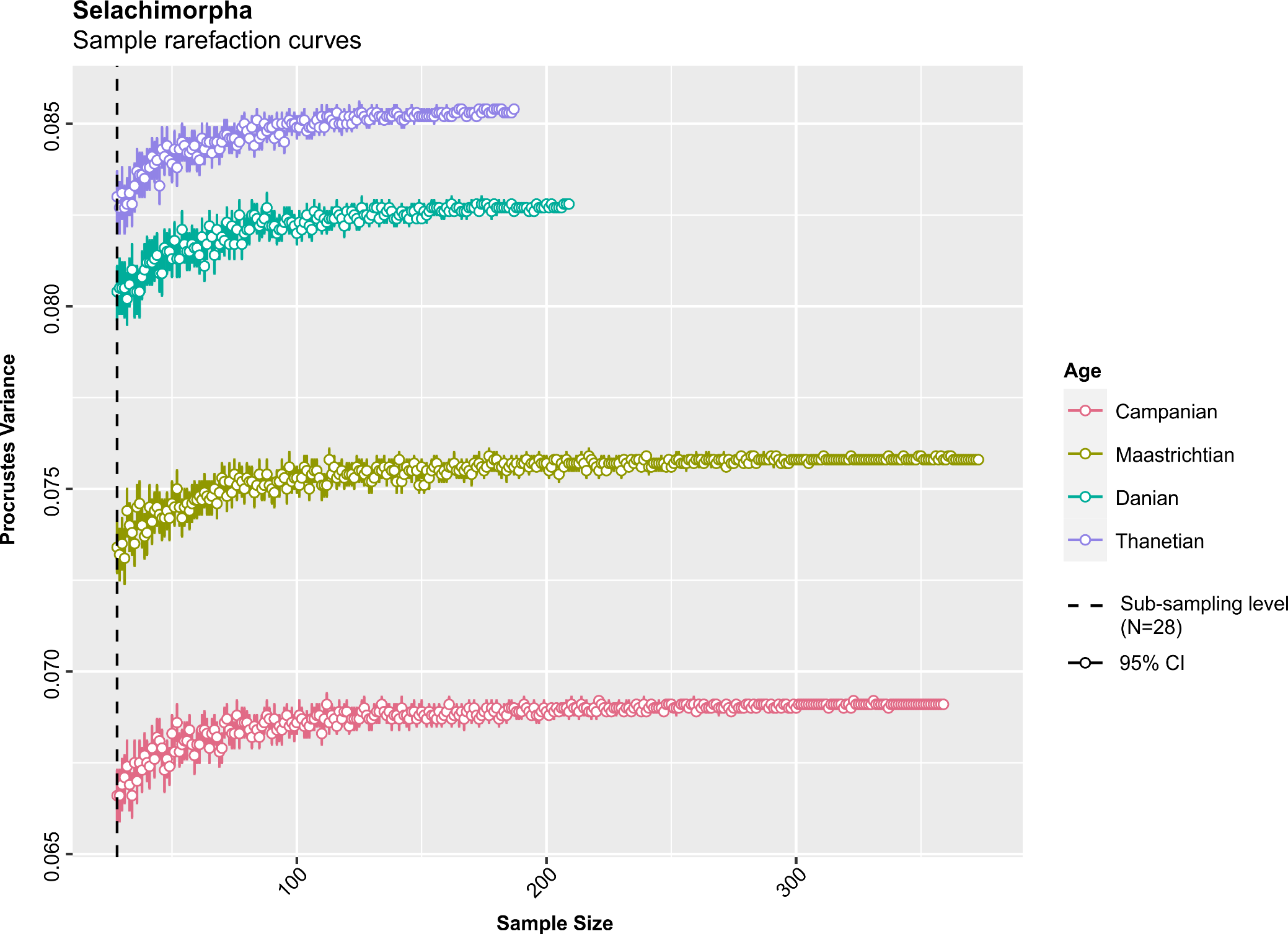


**Figure M. Global shark rarefaction profiles**. Based on the five-age time-binning scheme. CI = Confidence Intervals.


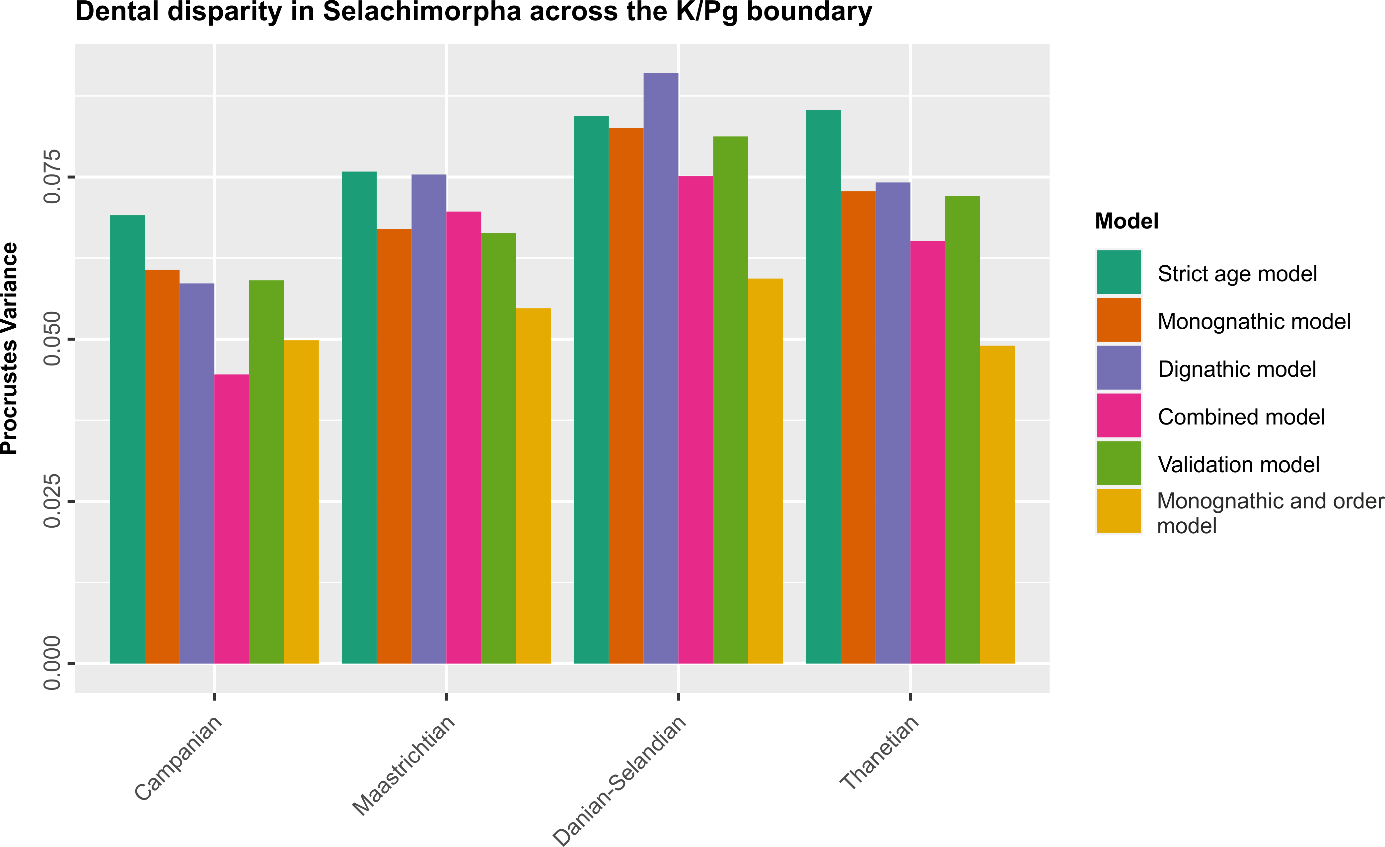


**Figure N. Estimates of disparity considering categorical predictor variables.** See supplementary text for model descriptions.


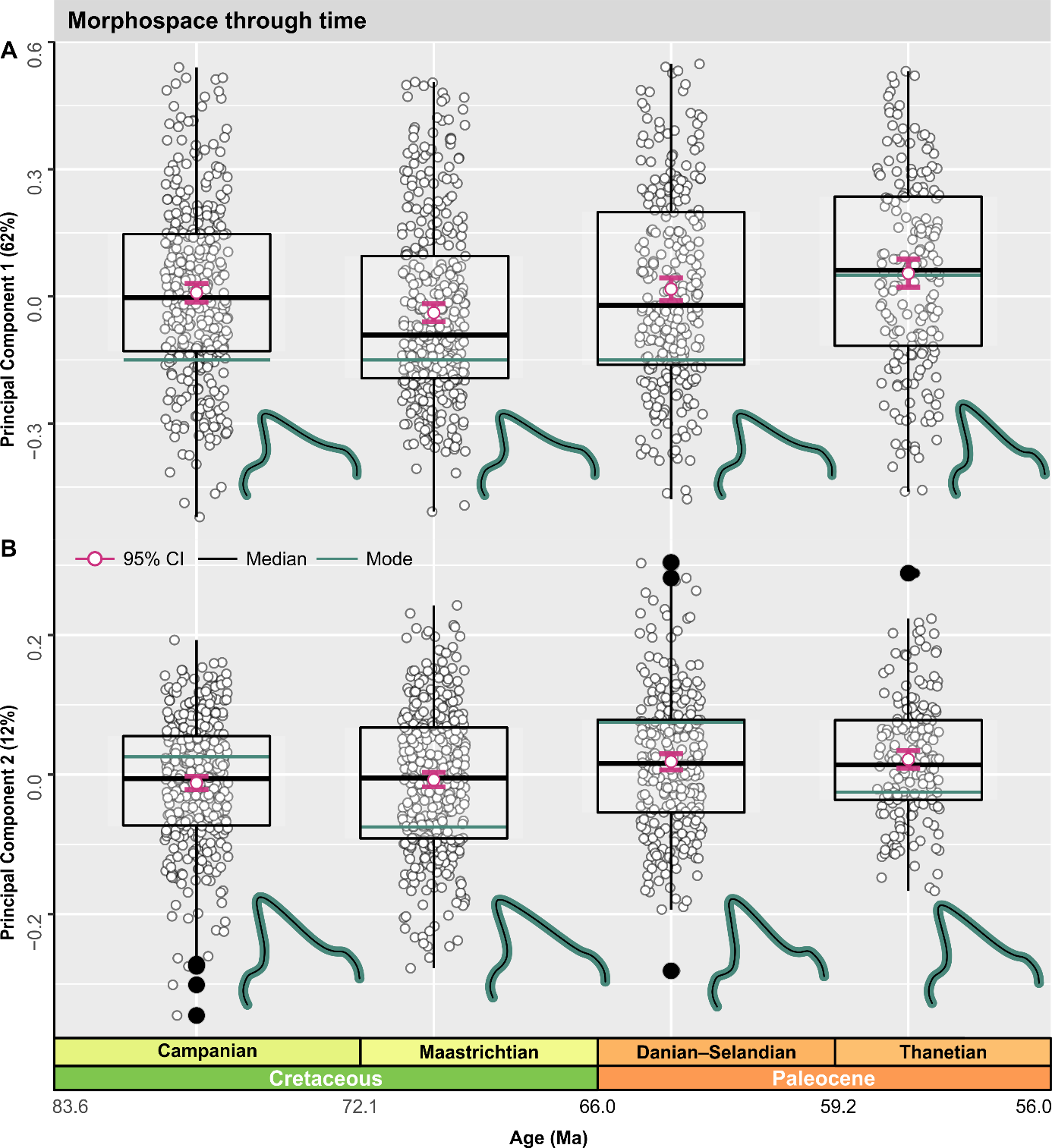


**Figure O. Global morphospace time-series. (A-B)** Jittered box-plots visualizing distribution on PC1/PC2 using the four-age time-binning scheme. Arithmetic mean with 95% confidence limits was derived from non-parametric bootstrapping; modal value and medians are shown for each level. TPS-grids indicate changes in the modal PC-value between ages time-bins.


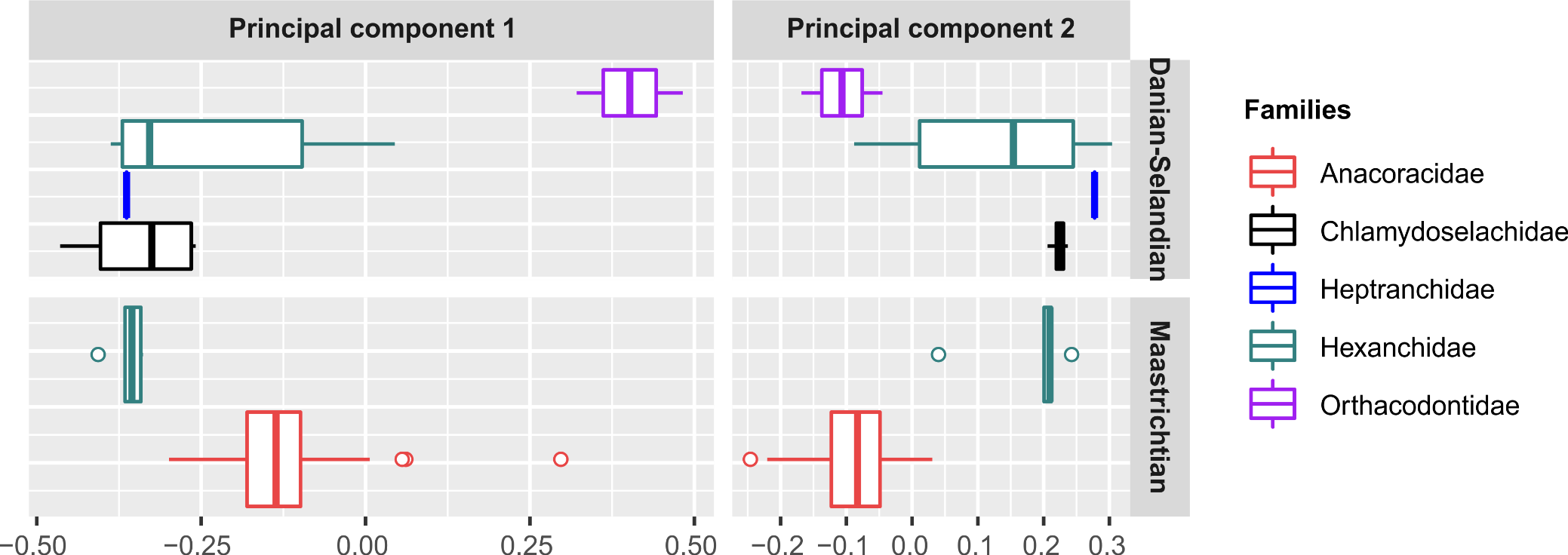


**Figure P. Morphospace dynamics**. Box-plots illustrate lamniform (Anacoracidae) versus hexanchiform (Chlamydoselachidae, Heptranchidae, Hexanchidae, Orthacodontidae) distribution in the Maastrichtian and Danian-Selandian time-bins.


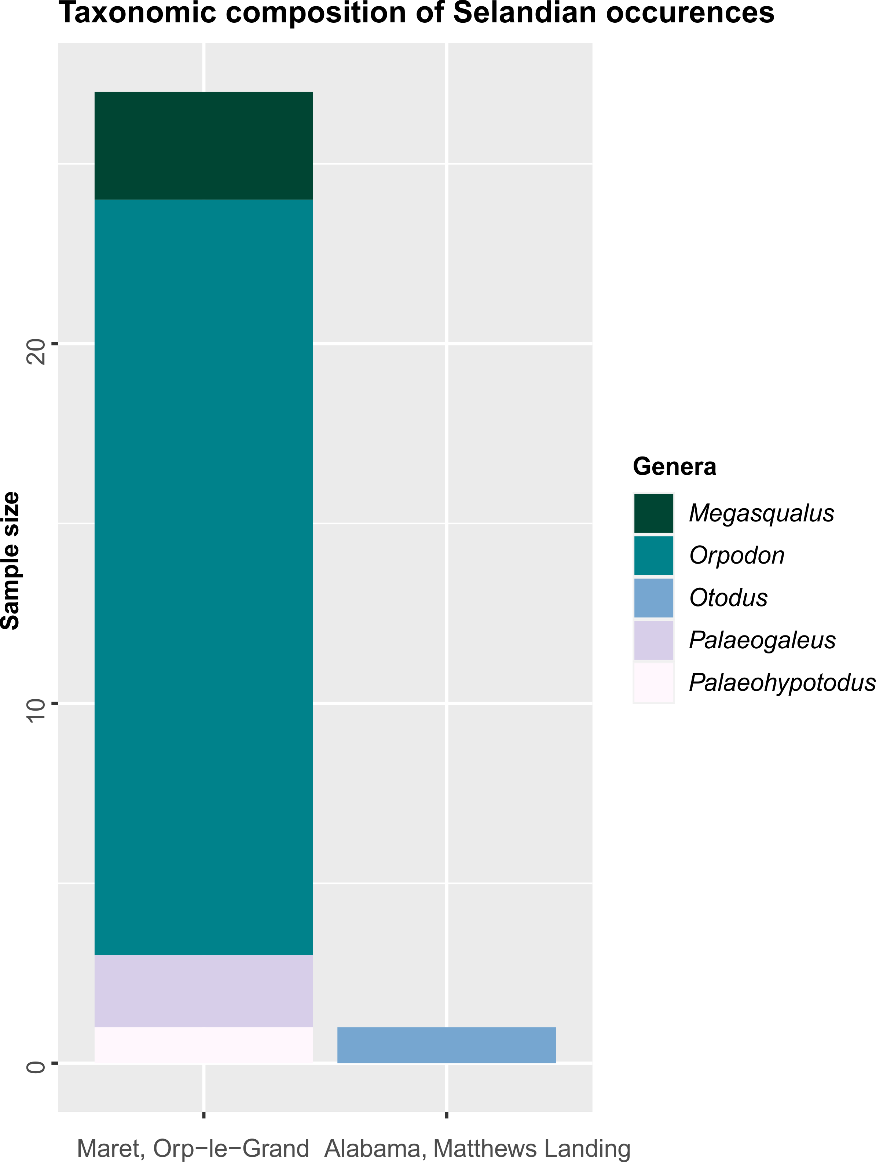


**Figure Q. Taxonomic composition and geographic distribution of Selandian genus-level occurrences.** Most samples represent lamniforms from the Orp-le-Grand locality in Belgium.

**
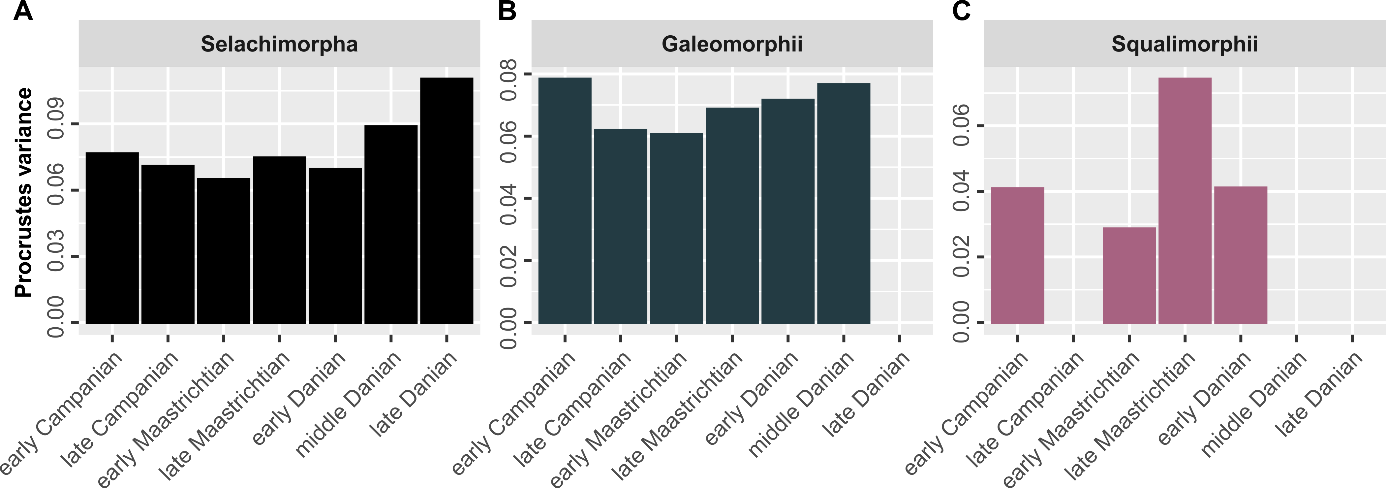
**

**Figure R.** **Sub-age disparity. (A–C)** Global disparity profiles for the superorder-level clades Selachimorpha, Galeomorphii, and Squalomorphii.


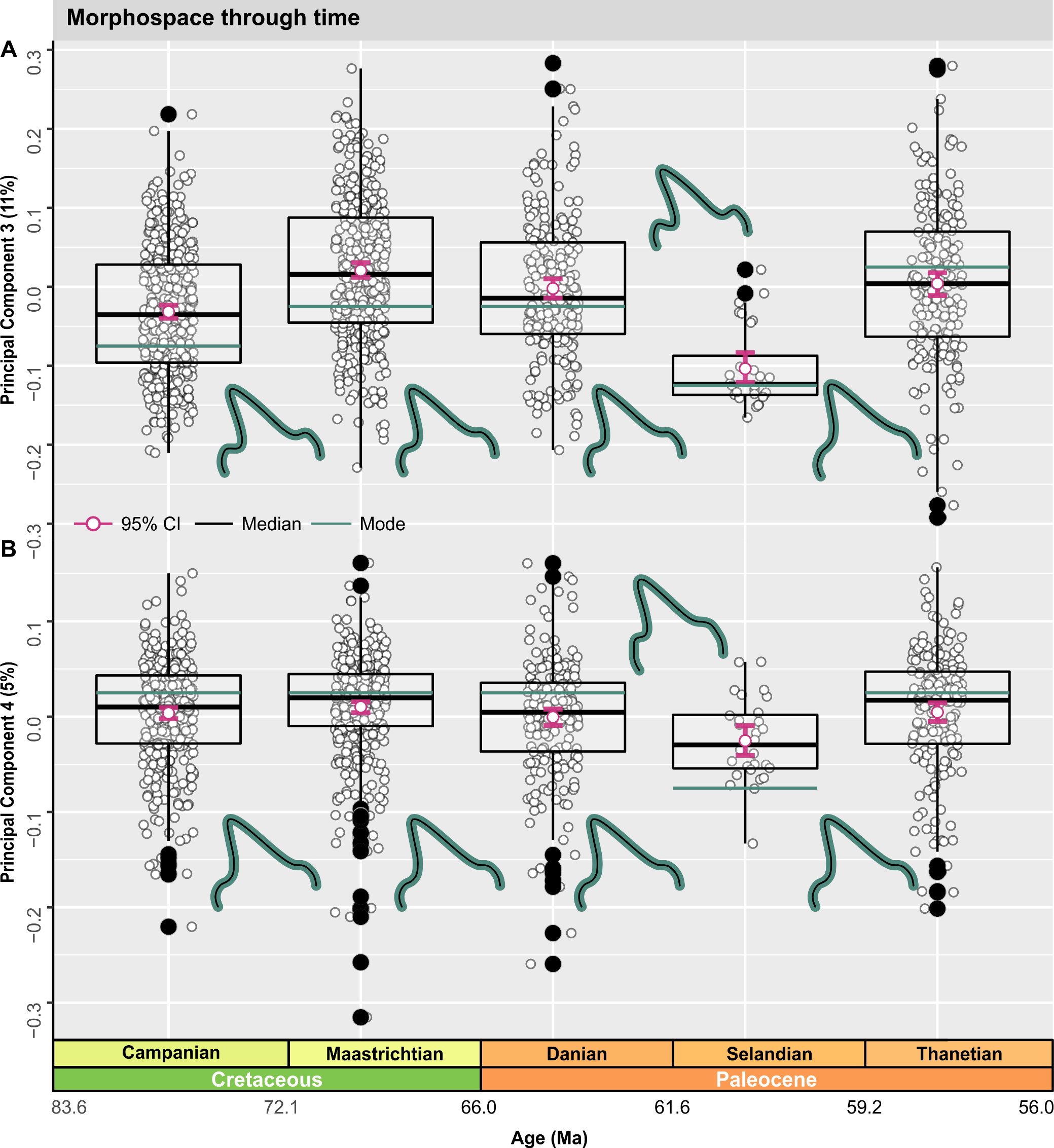


**Figure S.** **Global morphospace time-series analysis**. **(A–B)** Distribution of morphology along PC3/PC4 using the five-age time-binning scheme. Arithmetic mean with 95% confidence limits was derived from non-parametric bootstrapping; modal value and medians are shown for each level. TPS-grids indicate changes in the modal PC-value between ages.


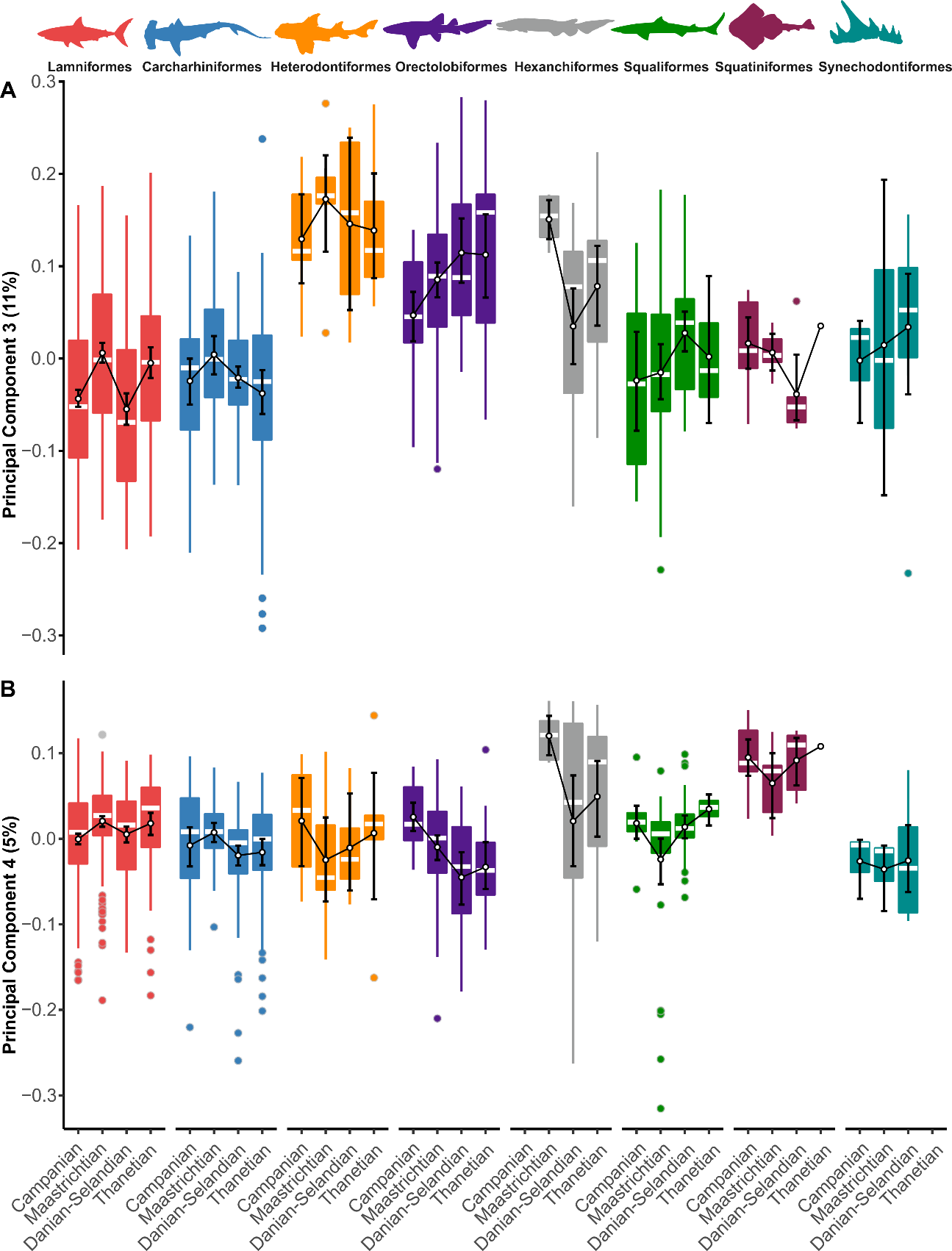


**Figure T.** **Morphospace for order-level clades using the four-age time-binning scheme.** **(A–B)** Patterns along PC3/PC4. Order-level clade colours and silhouettes: Lamniformes (red); Carcharhiniformes (blue); Heterodontiformes (orange); Orectolobiformes (purple); Hexanchiformes (grey); Squaliformes (light green); Squatiniformes (burgundy); †Synechodontiformes (dark green). Silhouettes generated by MB and Julius Csotonyi.


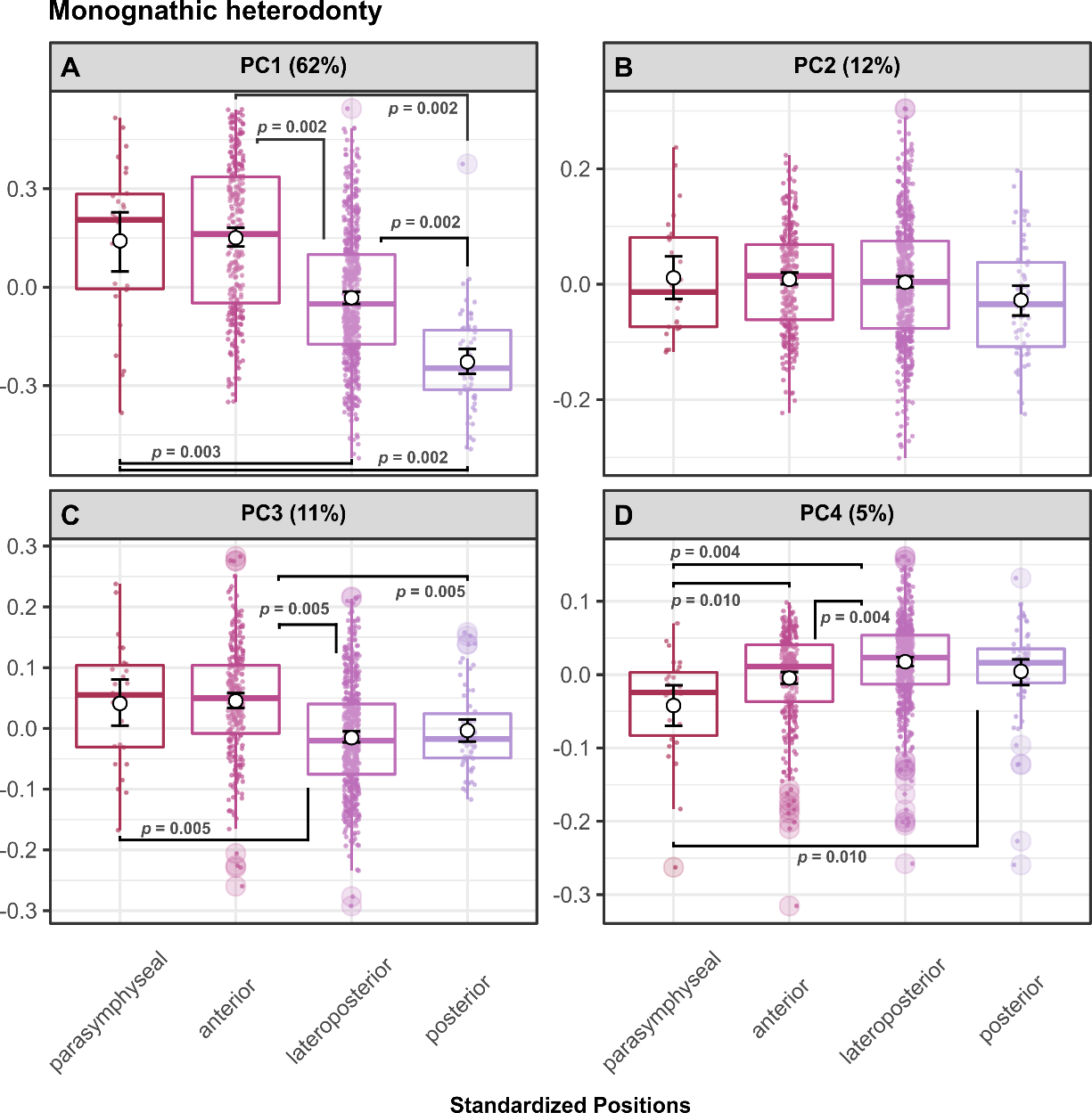


**Figure U. Box-and-whisker plot morphospace visualization of monognathic heterodonty. (A–D)** Distribution and computed 95% confidence intervals for standardized tooth positions (parasymphyseal=26, anterior=270, lateroposterior=578, posterior=58) along PC1–PC4. Significant FDR-adjusted p-values for multiple comparisons are indicated.


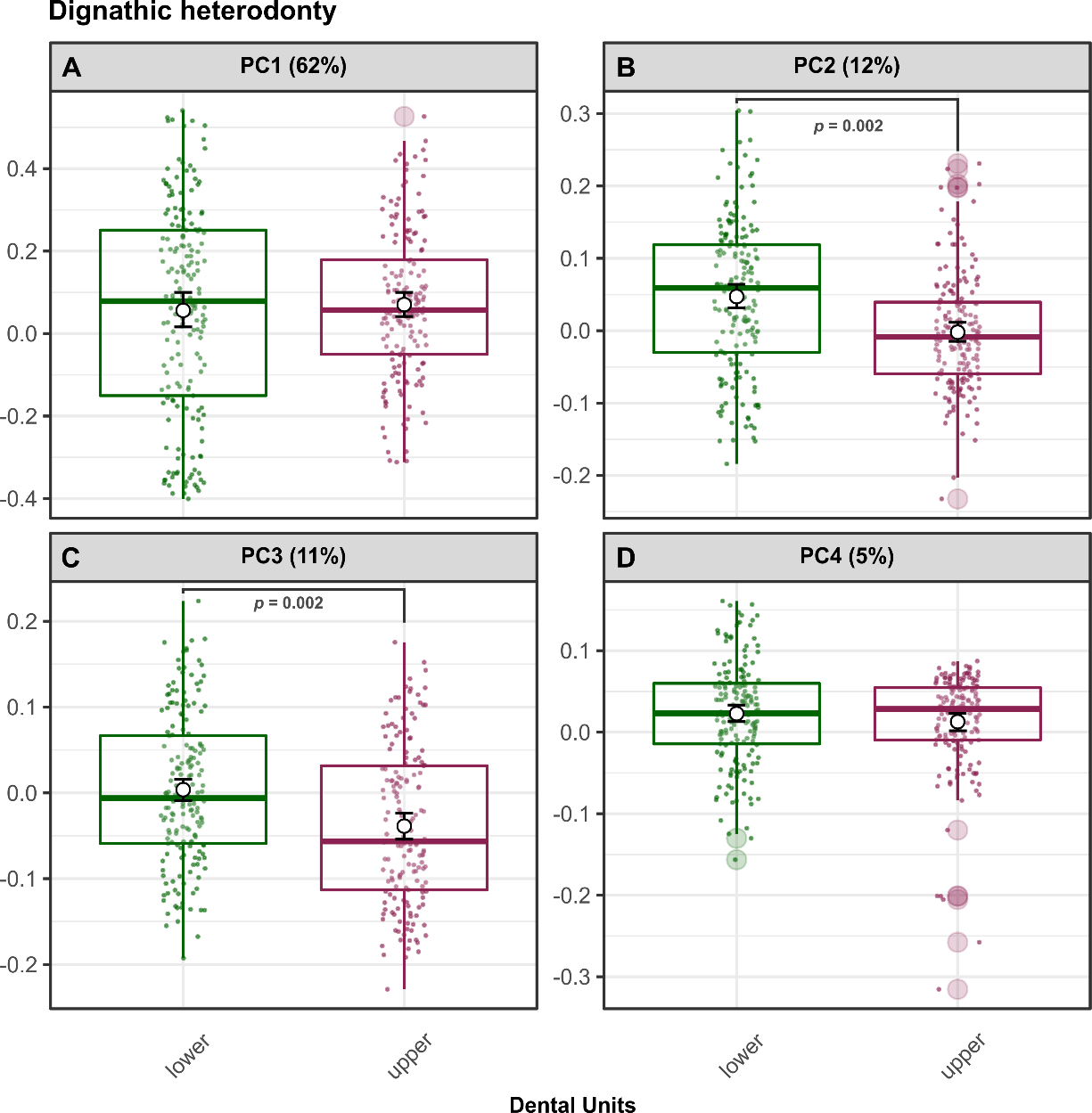


**Figure V. Box-and-whisker plot morphospace visualization of dignathic heterodonty. (A–D)** Distribution and computed 95% confidence intervals for dental units (lower=189, upper=176) along PC1–PC4. Significant FDR-adjusted p-values for multiple comparisons are indicated.


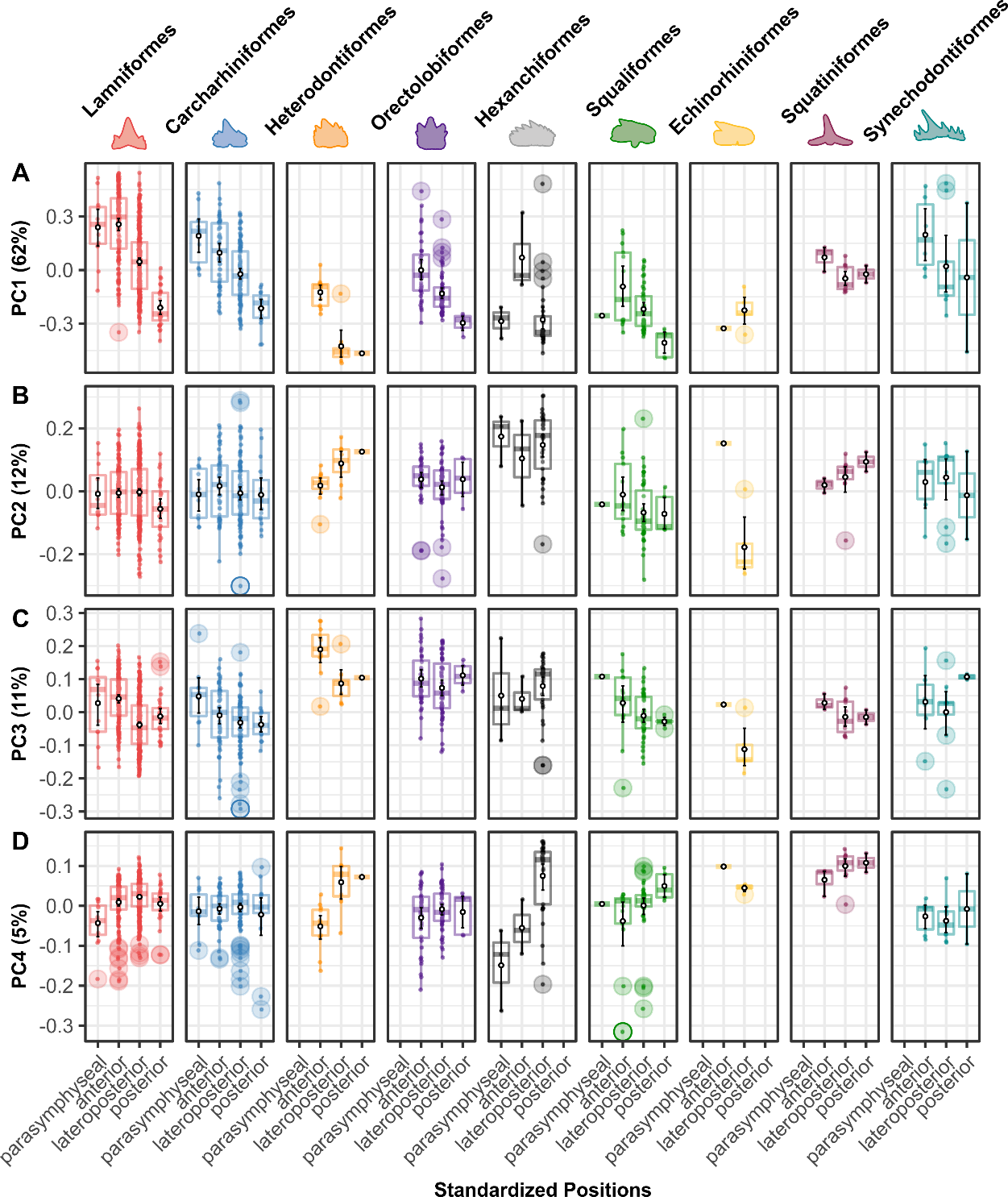


**Figure W. Standardized monognathic heterodont tooth positions for order-level clades along PC1–PC4.** Silhouettes generated by MB.


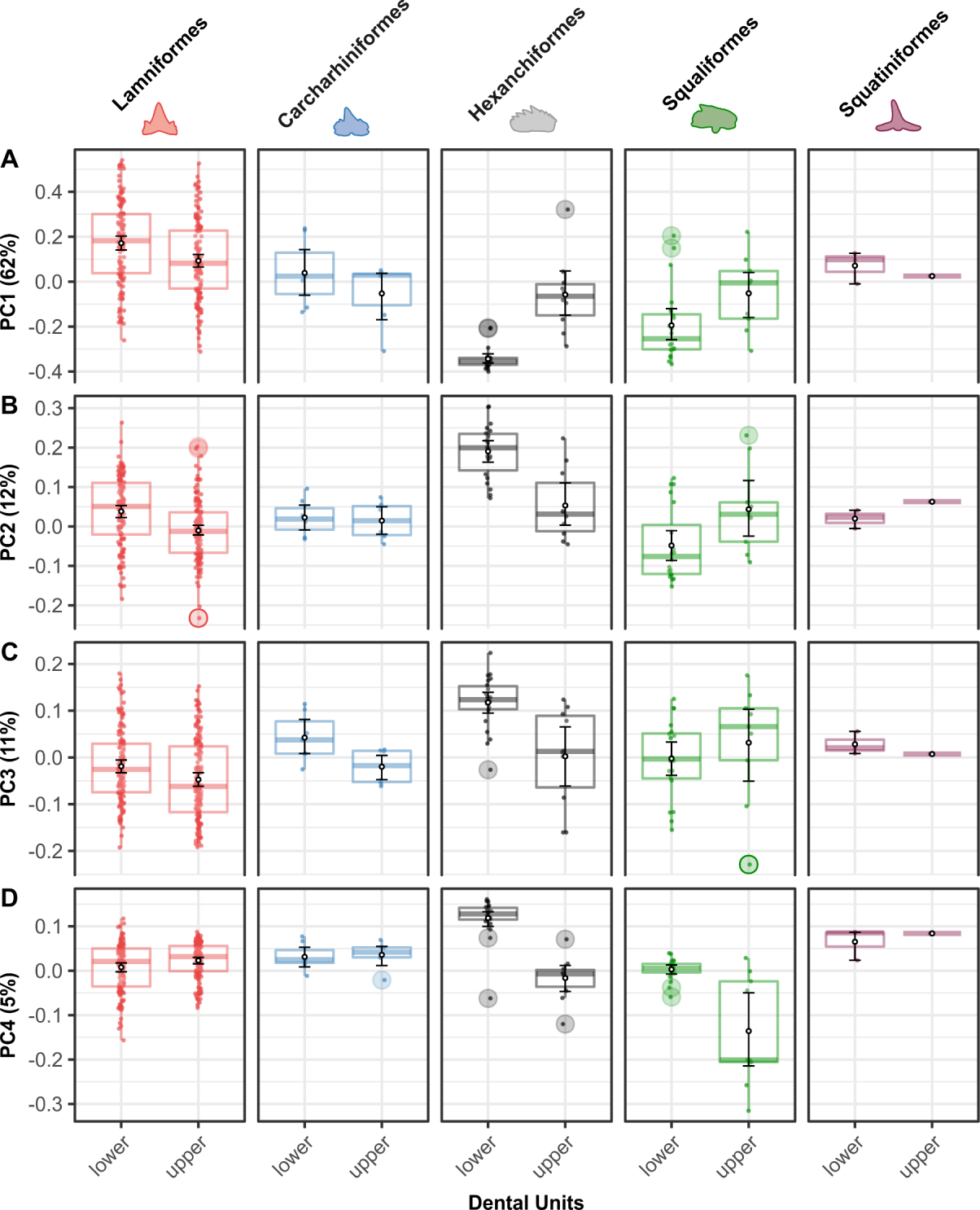


**Figure X. Standardized dignathic heterodont tooth positions for order-level clades along PC1–PC4.** Silhouettes generated by MB.


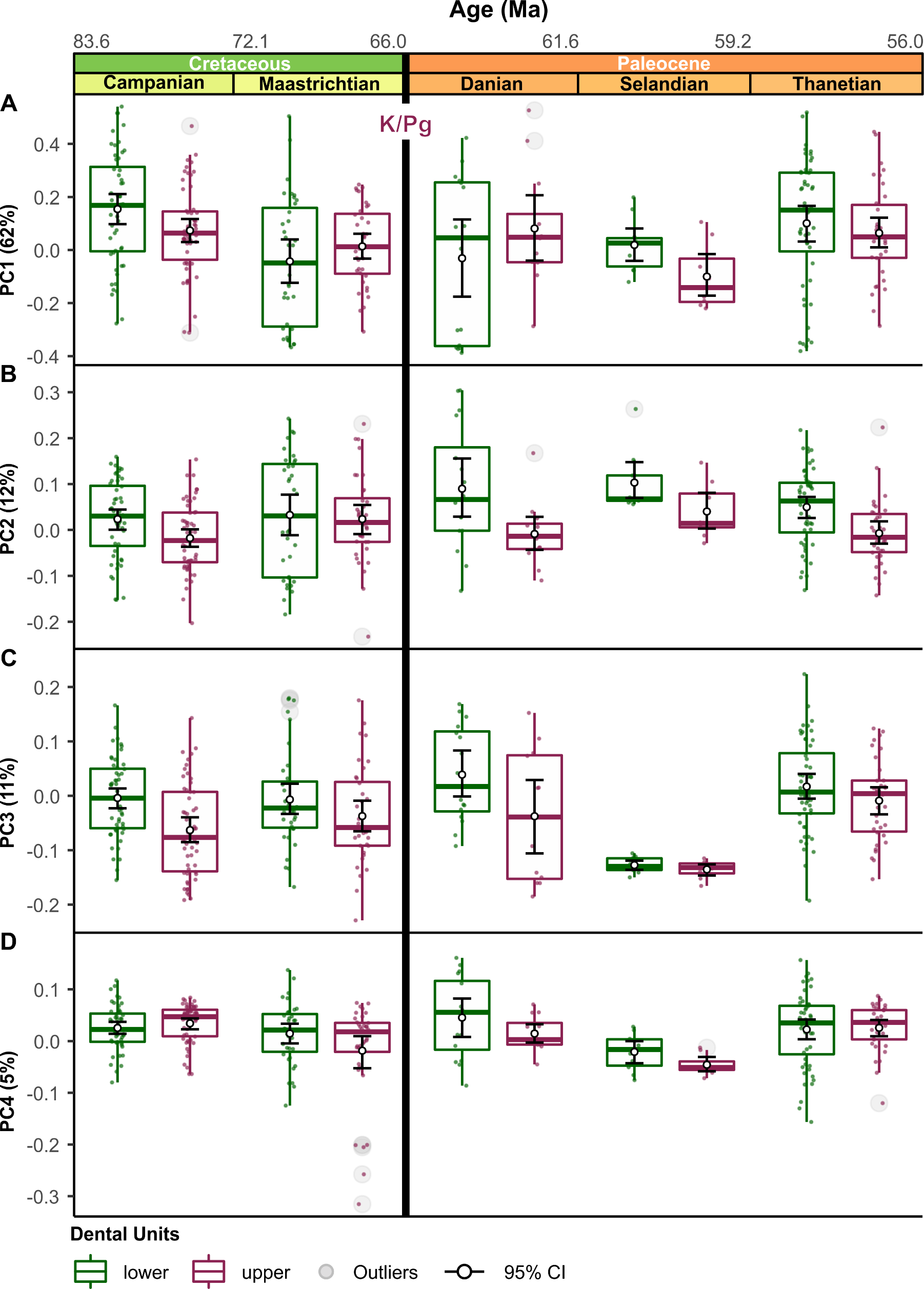


**Figure Y.** **Effects of dignathic heterodonty.** Morphospace analysis based on dental units (N=310) using the five-age time-binning scheme.


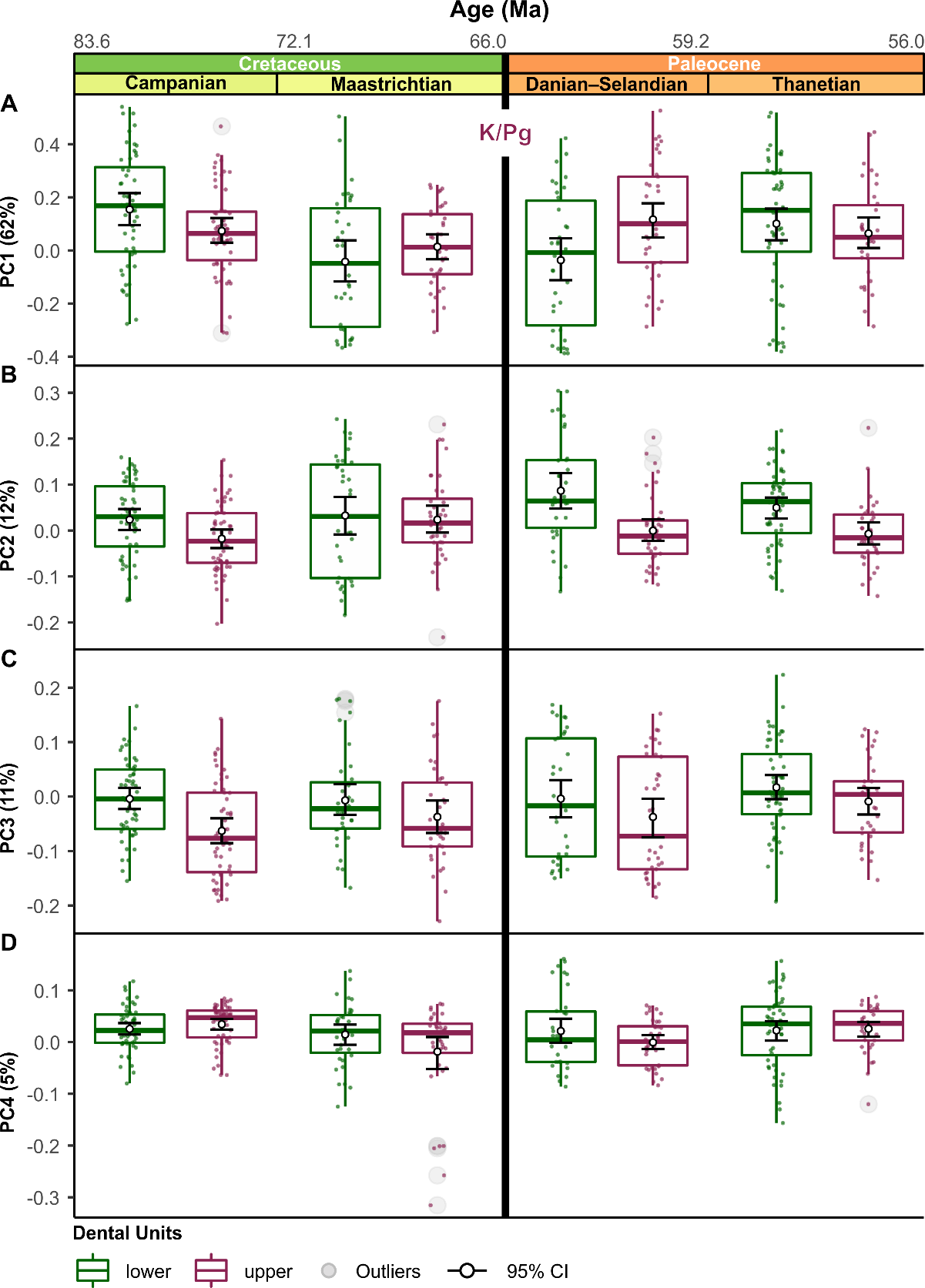


**Figure Z.** **Effects of dignathic heterodonty.** Morphospace analysis based on dental units (N=334) using the four-age time-binning scheme.


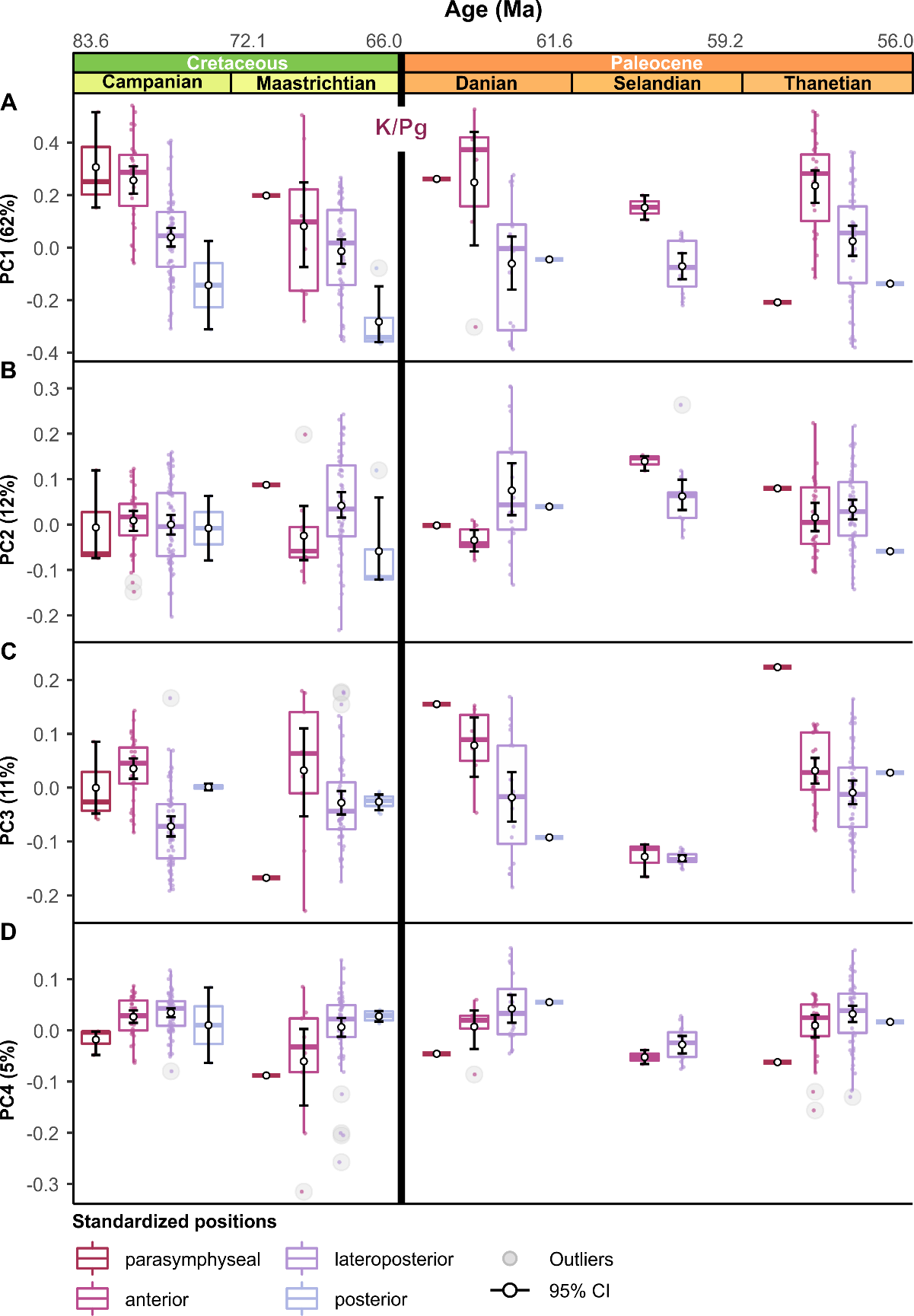


**Figure AA.** **Effects of monognathic heterodonty.** Morphospace analysis based on standardized tooth positions (N=858) using the five-age time-binning scheme. Intermediate tooth positions omitted.


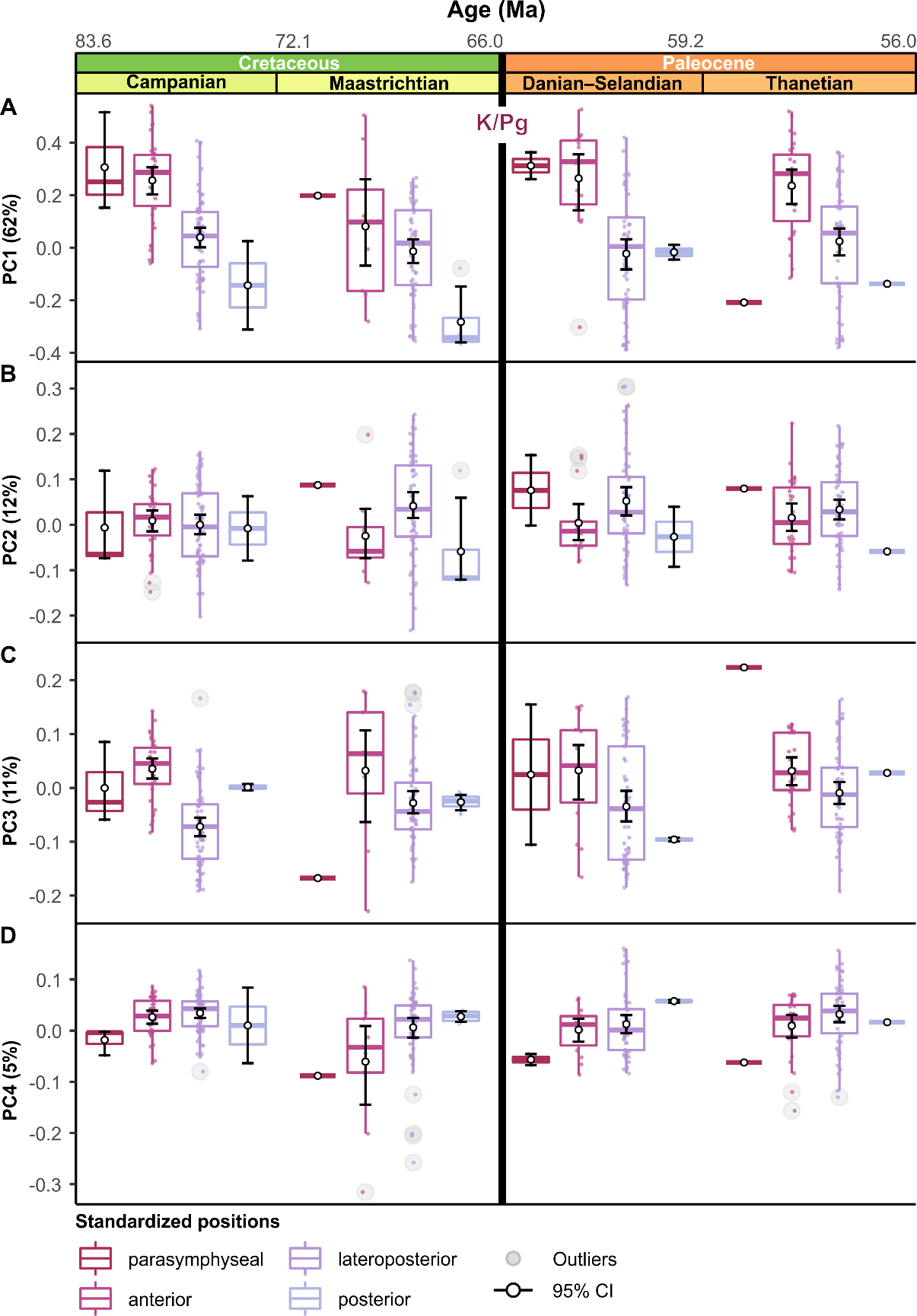


**Figure BB.** **Effects of monognathic heterodonty.** Morphospace analysis based on standardized tooth positions (N=897) using the four-age time-binning scheme. Intermediate tooth positions omitted.


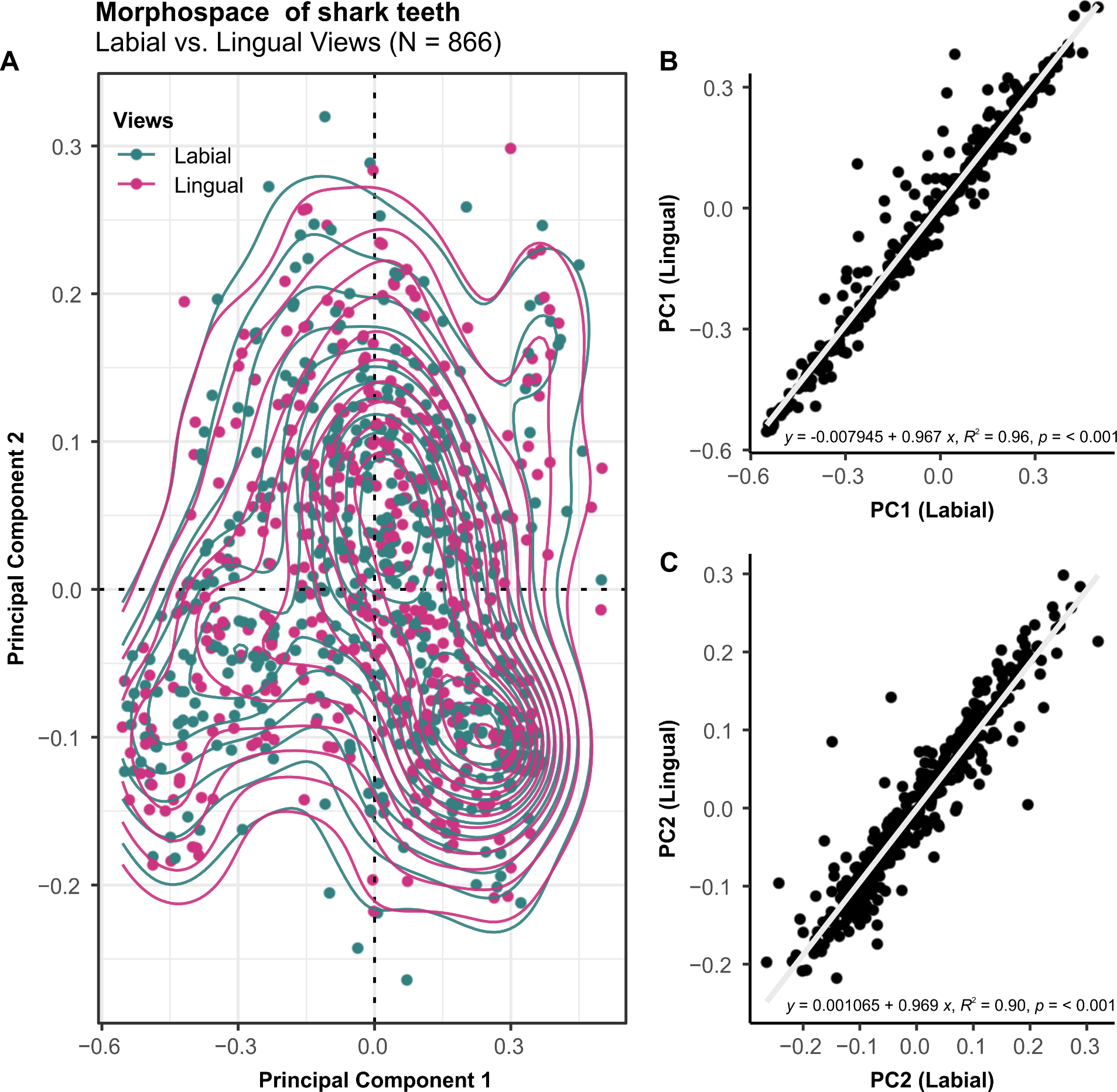


**Figure CC. Sensitivity analysis. (A)** Differences in morphospace occupation between lingual and labial views. **(B–C)** OLS-regression showing the linear relationship between scores.


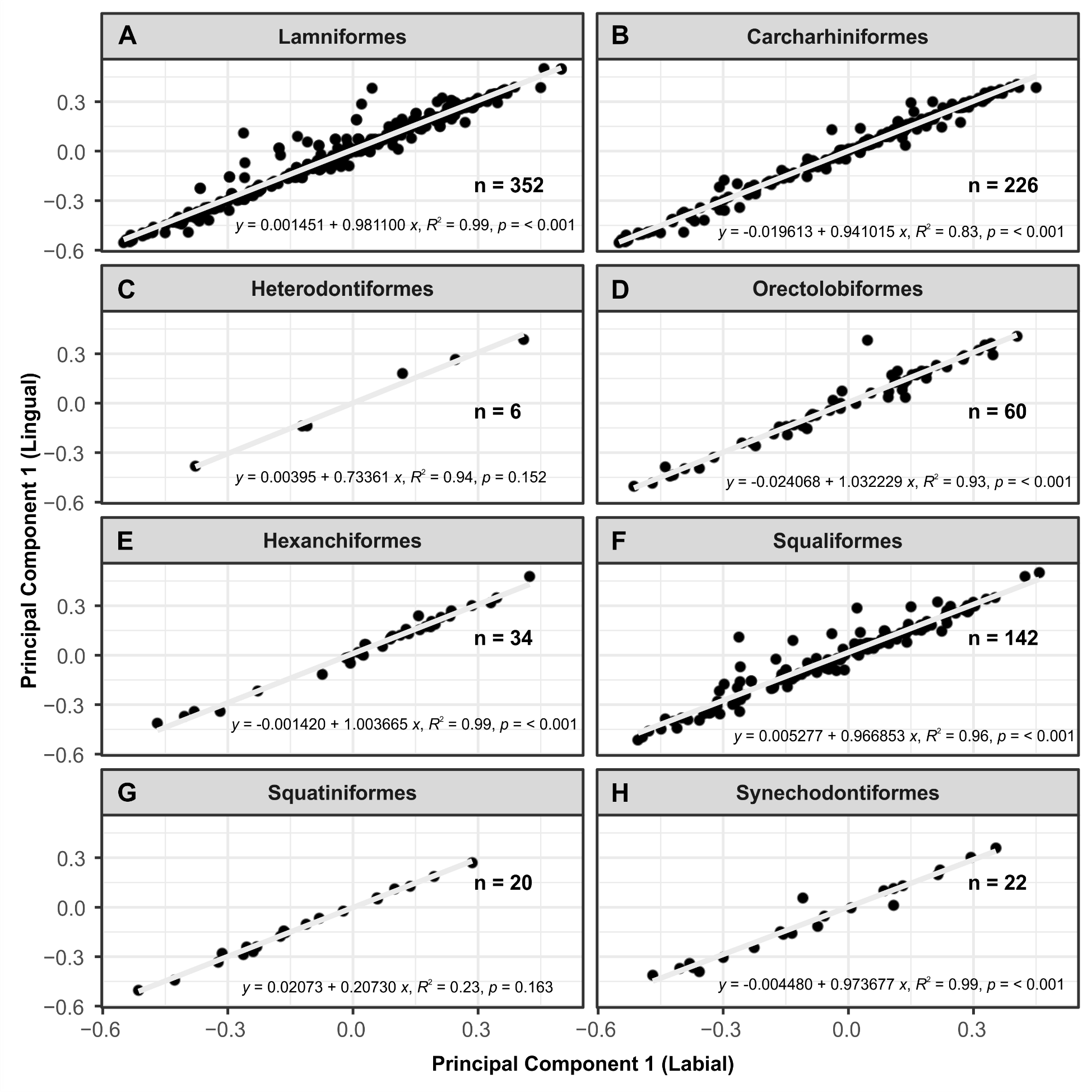


**Figure DD. Results of linear regression models. (A–H)** Relationship between lingual and labial views partitioned into order-level clades.

**Supplementary REferences**

1. Arnqvist G, Mårtensson T. Measurement error in geometric morphometrics: Empirical strategies to assess and reduce its impact on measures of shape. Acta Zool Acad Sci Hungaricae. 1998.

2. Fruciano C. Measurement error in geometric morphometrics. Dev Genes Evol. 2016;226: 139–158. doi:10.1007/s00427-016-0537-4

3. Claude J. Morphometrics with R. Springer Science & Business Media; 2008. Available: https://www.researchgate.net/publication/258885233

4. Cappetta H. Handbook of Paleoichthyology Volume 3E: Chondrichthyes · Mesozoic and Cenozoic Elasmobranchii: Teeth. Munich, Germany: Verlag Dr. Friedrich Pfeil; 2012.

5. Bazzi M, Kear BP, Blom H, Ahlberg PE, Campione NE. Static Dental Disparity and Morphological Turnover in Sharks across the End-Cretaceous Mass Extinction. Curr Biol. 2018. doi:10.1016/j.cub.2018.05.093

6. Siverson M. A new large lamniform shark from the uppermost Gearle Siltstone (Cenomanian, Late Cretaceous) of Western Australia. Trans R Soc Edinburgh, Earth Sci. 1999;90: 49–66. doi:10.1017/S0263593300002509

7. Shimada K. Dentition of the late cretaceous lamniform shark, cretoxyrhina mantelli from the niobrara chalk of kansas. J Vertebr Paleontol. 1997;17: 269–279. doi:10.1080/02724634.1997.10010974

8. Bookstein FL. Morphometric Tools for Landmark Data. Morphometric Tools for Landmark Data. Cambridge University Press; 1991. doi:10.1017/cbo9780511573064

9. Brownrigg R. maps: Draw Geographical Maps. 2018. Available: https://cran.r-project.org/package=maps

10. Wickham H. ggplot2 Elegant Graphics for Data Analysis. Journal of the Royal Statistical Society: Series A (Statistics in Society). 2016. doi:10.1007/978-3-319-24277-4

11. Barfort S. mapDK: Maps of Denmark. 2015.
