# Supplementary material for: Tooth morphology elucidates shark evolution across the end-Cretaceous mass extinction
Source: PLoS Biol. 2021 Aug 10;19(8):e3001108. doi: 10.1371/journal.pbio.3001108 (PMC8354442; doi:10.1371/journal.pbio.3001108)
Supplement: S5 Data — (PDF) [file pbio.3001108.s006.pdf]

Tooth morphology elucidates shark evolution across the end-cretaceous mass extinction

Mohamad Bazzi, Nicolás E. Campione, Henning Blom, Per E. Ahlberg, and Benjamin P. Kear
Department of Organismal Biology, Uppsala University.

Mohamad.Bazzi@ebc.uu.se

Code compiled and maintained by Mohamad Bazzi

Contact: Mohamad.Bazzi@ebc.uu.se, Mohammed_Bazzi@hotmail.com

Compiled: 2020-07-29

Last updated: 2021-06-30

Code files included: read points function.R: Function to generate evenly spaced points. Confidence and Prediction Intervals.r: Function to estimate parametric confidence and prediction intervals. Morphological Disparity with Bootstrap.R: Function to compute Procrustes variance and rarefaction. btShapes.R: Function to generate a backtransform morphospace (modified from Olsen 2017) gms.R: Function to compute descriptive statistics.

Data files included: S1 Data.xlsx - Global occurrence dataset.

All analyses were done using R version 4.0.5 (R Core Team 2021).

This document is an annotated version of the R code used to run geometric morphometric analyses

Text Blurb

Bazzi et al. analyse the tooth morphology of sharks across the end-Cretaceous mass extinction, 66 million years ago. They show that while generally unaffected, some apex predator shark lineages were selectively impacted. Changing habitats and the differential survival of 'fish-eating' sharks also reveals responses to ecological cataclysm.

Setup

```
options(width=60)
knitr::opts_chunk$set(echo=TRUE,tidy=T,fig.show="hide",dev="jpeg",tidy.opts=list(width.cutoff=60))
Sys.setenv(RSTUDIO_PDFLATEX = "latexmk")
```

Required Libraries

```
packages <- c("devtools", "sp", "xlsx", "geomorph", "ggplot2",
    "scatterpie", "ggrepel", "viridis", "Morpho", "Momocs", "colorspace",
    "reshape2", "tidyverse", "dplyr", "plotrix", "rcartocolor",
    "pals", "smatr", "ggpubr", "car", "shapes", "harrypotter")
```

```
lapply(packages, library, character.only = TRUE)
```

Functions

```
# Re-sample and make points equidistant.
source(file = "Core R Functions/read points function.R")
# Confidence and prediction intervals.
source(file = "Core R Functions/Confidence and Prediction Intervals.r")
# Function to compute Procrustes variance, rarefaction, and
# bootstrap statistic.
source(file = "Core R Functions/Morphological Disparity with Bootstrap.R")
# Backtransform morphospace.
source(file = "Core R Functions/btShapes.R")
# Numerical descriptive statistics.
source(file = "Core R Functions/gms.R")
# Function to compute the modal value.
estimate_mode <- function(x) {
    d <- hist(x, plot = FALSE)
    mode <- d$mids[which.max(d$counts)]
    return(mode)
}
# Disparity with permutation.
disparity.calc <- function(gpa, data, ages) {
    nas <- which(is.na(data[, ages]))
    if (length(nas) > 0)
        gm.data <- geomorph.data.frame(coords = gpa$coords[,
            , rownames(data)[-nas]], ages = data[-nas, ages]) else gm.data <- geomorph.data.frame(coord
        , rownames(data)], ages = data[, ages])
    disp <- morphol.disparity(coords ~ ages, groups = ~ages,
        iter = 999, data = gm.data)
    return(disp)
}
# ANOVA with RRPP.
anova.test <- function(scores, bins, axes) {
    # Clean up data.
    nas <- which(is.na(bins))
    if (length(nas) > 0) {
        scores <- scores[-nas, ]
        bins <- bins[-nas]
    }
    # General: applied across all axes.
    rrpp.data.all <- rrpp.data.frame(scores = scores, bins = bins)
    anova.all <- lm.rrpp(f1 = scores ~ bins, iter = 999, RRPP = T,
        print.progress = TRUE, data = rrpp.data.all)
    pw.all <- pairwise(fit = anova.all, groups = interaction(rrpp.data.all$bins))
    s.pw <- summary(pw.all, test.type = "dist", confidence = 0.95,
        stat.table = TRUE)
    p.values.all <- s.pw$pairwise.tables$P
    p.values.all[lower.tri(p.values.all)] <- p.adjust(p.values.all[lower.tri(p.values.all)],
        method = "fdr")
    axes.res <- vector(mode = "list", length = length(axes))
    # Axes specific and rounded to 3 digits.
    for (i in 1:length(axes)) {
```

```
        rrpp.ax <- rrpp.data.frame(scores = scores[, axes[i]],
            bins = bins)
        anova.ax <- lm.rrpp(f1 = scores ~ bins, data = rrpp.ax,
            iter = 999, print.progress = TRUE)
        pw.ax <- pairwise(fit = anova.ax, groups = interaction(rrpp.ax$bins))
        o.summary <- summary(pw.ax, test.type = "dist", confidence = 0.95,
            stat.table = TRUE)
        p.values.ax <- o.summary$pairwise.tables$P
        p.values.ax[lower.tri(p.values.ax)] <- p.adjust(p.values.ax[lower.tri(p.values.ax)],
            method = "fdr")
        axes.res[[i]] <- round(p.values.ax, 3)
    }
    return(list(anova.results = anova.all, adjusted.p = p.values.all,
        axes.results = axes.res))
}
```

Data wrangling

We relied mostly on the primary literature of fossil sharks to obtain images of teeth. This resulted in three different datasets: 1) a global occurrence dataset (N=1239) summarizing detailed information (e.g., provenance, age, and jaw position) for each distinct specimen; 2) a library of orientation standardized tooth images used subsequently to digitize landmarks and sliding semi-landmarks; and 3) a TPS-file containing the coordinate data of landmarks. All landmark configurations are re-sampled and made equidistant using the **read2Dtps.noLMs** function (see details below).

```
# 1. Landmark data.
LMs <- read2Dtps.noLMs(file = "Image Library/tpsKPg.TPS", ncurve = 2,
    divide.curve = TRUE, curve.1.pts = 79, curve.2.pts = 81)
# 2. Occurrence data frame.
Data <- read.xlsx(file = "Morphometric & Other Excel Datasets/Tooth DataFrame.xlsx",
    sheetIndex = 1)
Data <- subset(x = Data, subset = Data$Used == "x")
# 3. Set rownames.
rownames(Data) <- paste(Data$File.Name, Data$File.Type, sep = "")
# 4. Re-arrange the levels in the order of appearance in
# the data frame.
Data$Order <- factor(Data$Order, levels = c("Lamniformes", "Carcharhiniformes",
    "Heterodontiformes", "Orectolobiformes", "Hexanchiformes",
    "Squaliformes", "Echinorhiniformes", "Squatiniformes", "Synechodontiformes"))

# 5. Subset landmark coordinates to match rownames of the
# data frame.
Landmarks <- LMs$coords[, , rownames(Data)]
# 6. Write landmarks to TPS.file and accompanying sliders
# file.
writeland.tps(Landmarks, "Coordinates.tps", scale = NULL, specID = TRUE)
write.table(x = as.matrix(LMs$sliders[-80, ]), file = "Sliders.txt")
```

Exploratory Data Analysis (EDA)

Sampling is uneven among clades. Lamniformes (Mackerel sharks) and Carcharhiniformes (Ground sharks) are particularly well represented. Orectolobiformes (Carpet sharks) and Squaliformes (Dogfish sharks) are moderately represented. Heterodontiformes (Bullhead sharks), Hexanchiformes (cow and frilled sharks),

Echinorhiniformes (Bramble sharks), Squatiniformes (Angel sharks), and Synechodontiformes are poorly represented.

```
# 1. Visualization of global sample size.
v <- barplot(summary(Data$Order), xaxt = "n", xlab = "", ylab = "Counts",
    legend = F, axis.lty = 3, main = "Sample size by clade",
    cex.lab = 0.7, cex.axis = 0.7)

text(v, par("usr")[3], labels = levels(Data$Order), font = 1,
    srt = 45, adj = c(1.1, 1.1), xpd = TRUE, cex = 0.7, font.lab = 2)
```

```
dev.off()
```

```
## null device
##           1
```

```
# 2. Taxonomic data.
data_mtrx <- read.xlsx(file = as.matrix("Morphometric & Other Excel Datasets/Global Selachimorpha Sampl
    sheetIndex = 1, header = T)
rownames(data_mtrx) <- data_mtrx[, 1]
data_mtrx[, 1] <- NULL
data_mtrx <- as.matrix(data_mtrx)
# Color vector.
col.box <- c("#E84646", "#377EB8", "darkorange", "purple4", "#9D9E9E",
    "green4", "goldenrod1", "violetred4", "darkcyan")
# 3. Plot sample sizes
layout(matrix(c(1, 1), 1, 1), respect = T)
bar <- barplot(data_mtrx, col = scales::alpha(col.box, 0.45),
    border = col.box, space = 0.15, font.axis = 2, xlab = "",
    ylab = "Sample Size (N)", axes = FALSE, axisnames = FALSE,
    axis.lty = 3, font.lab = 2, legend.text = levels(Data$Re.Order),
    args.legend = list(x = "topright", bty = "n"))
text(bar, par("usr")[3], labels = c("Campanian", "Maastrichtian",
    "Danian", "Selandian", "Thanetian", "D/S"), font = 1, srt = 45,
    adj = c(1.1, 1.1), xpd = TRUE)
axis(2, las = 3, tick = 0.25, font = 1, lty = 1, lwd = 1)

abline(v = 5.85, col = "black", lwd = 1.5, lty = 2)
dev.off()
```

```
## null device
##           1
```

Spatiotemporal visualization of data sources

```
# 1. Read in data frame.
Map.Data <- read.xlsx(file = "Morphometric & Other Excel Datasets/Geographic Material Graph.xlsx",
    sheetIndex = 1, header = T)
Map.Data <- Map.Data[-1]
# 2. Define the basic characteristics of the plot.
world <- map_data("world")
world <- fortify(world)
p <- ggplot()
p <- p + geom_polygon(data = world, aes(x = long, y = lat, group = group),
    alpha = 0.8, colour = "lightgray", fill = "darkgrey", size = 0.15) +
    coord_quickmap(expand = T)
```

```r
# 3. Plot.
p <- p + geom_scatterpie(aes(x = lon, y = lat, group = region,
    r = sqrt(totalSample)/2), data = Map.Data, cols = LETTERS[1:4],
    alpha = 0.8, sorted_by_radius = T) + theme_bw() + theme(legend.position = "top") +
    scale_fill_hp(discrete = TRUE, option = "ronweasley2") +
    geom_text_repel(data = Map.Data, aes(x = lon, y = lat, label = region),
        size = 3, box.padding = unit(0.35, "lines"), point.padding = unit(0.3,
            "lines"))
print(p)
```

Digitization scheme using ggplot

Type 1 landmarks delimited the crown-root junction (n=2), with Type 2 landmarks defining the point of maximum curvature at the tooth apex.

```r
# 1. Prepare labels for sequential landmark points.
ldlab <- paste("LM", 1:160, sep = "")
# 2. Convert array to data frame.
Cretalamna <- as.data.frame(LMs$coords[, , "Cretalamna-maroccana_Case-et-al-2017_3E1.jpg"])
# 3. Plot.
ggplot(Cretalamna, aes(x = V1, y = V2)) + geom_point(shape = 21,
    size = 3, fill = "grey90", colour = "black") + geom_text(aes(label = ldlab),
    hjust = 0, vjust = 0, cex = 1.5, colour = "purple", fontface = 2) +
    labs(title = "Resampled and Scaled Points", xlab = "", ylab = "",
        subtitle = "") + theme_classic(base_size = 12) + coord_fixed() +
    annotate("text", x = 650, y = 350, label = "Type 1", colour = "black",
        fontface = 2) + annotate("text", x = 650, y = 500, label = "Type 2",
    colour = "black", fontface = 2)
```

Count and proportion of teeth in labial and lingual views

Anatomical views are shown by order and age. Teeth photographed in labial perspective constitute the main source of shape variation.

```r
# Data frame.
df.views <- read.xlsx(file = "Morphometric & Other Excel Datasets/Lingual vs. Labial Teeth.xlsx",
    sheetIndex = 1)
# Re-level orders.
df.views$Orders <- factor(df.views$Orders, levels = c("Lamniformes",
    "Carcharhiniformes", "Heterodontiformes", "Orectolobiformes",
    "Hexanchiformes", "Squaliformes", "Echinorhiniformes", "Squatiniformes",
    "Synechodontiformes"))
# Re-level stages.
df.views$Facet <- factor(df.views$Facet, levels = c("Campanian",
    "Maastrichtian", "Danian-Selandian", "Thanetian"))
melt.view <- melt(df.views)
```

```
## Using Orders, Stage, Facet as id variables
```

```r
# Grouped stacked bar plot.
ggplot(melt.view, aes(y = value, x = Facet, fill = variable,
    colour = variable)) + geom_bar(position = "dodge", stat = "identity",
    colour = "black") + xlab("") + ylab("Count") + scale_color_viridis(discrete = TRUE,
    option = "A") + scale_fill_viridis(discrete = TRUE, option = "A") +
    facet_wrap(~Orders, scales = "free_y", shrink = T) + theme_bw() +
    theme(axis.title = element_text(color = "#666666", face = "bold",
```

```
        size = 8), axis.text.y = element_text(angle = 90, size = 8),
        axis.text.x = element_text(angle = 45, vjust = 0.5, hjust = 0.75,
            size = 8), axis.line = element_line(linetype = "solid"),
        panel.spacing = unit(0, "lines"), aspect.ratio = 3/4)
```

Selandian sample

```
Selandian.Df <- subset(Data, subset = Data$Age == "Selandian")
Selandian.Df <- (table(Selandian.Df$Locale, Selandian.Df$Genus))
Sel.melt <- melt(Selandian.Df)
Sel.melt$Var1 <- relevel(x = Sel.melt$Var1, ref = "Maret, Orp-le-Grand")
# Stacked bar plot
ggplot(data = Sel.melt, aes(x = Var1, y = value, fill = Var2)) +
    geom_bar(stat = "identity") + ylab("Sample size") + scale_fill_manual(values = sequential_hcl(5,
    palette = "PuBuGn")) + scale_color_manual(values = sequential_hcl(5,
    palette = "PuBuGn")) + theme_grey() + labs(title = "Taxonomic composition of Selandian occurences")
    theme(axis.title = element_text(size = 8), strip.text.x = element_text(size = 8,
        face = "bold"), axis.text.y = element_text(angle = 90,
        size = 8), axis.text.x = element_text(size = 8, angle = 45,
        vjust = 0.8), aspect.ratio = 2)
```

Analysis

Procrustes superimposition

Result: 6 GPA iterations to converge. The dimensionality for 2D data is twice the number of landmarks minus four: **Here, Dp=160*2-4. The scaling of landmark configurations to unit centroid size is commonly referred to as the partial GPA (Dryden & Mardia 2016). The alignment procedure produce a curved (non-linear:** a Riemannian manifold**) multidimensional space that is related to Kendall's shape space.

```
# 1. GPA using minimizing bending energy.
GPA <- gpagen(A = Landmarks, curves = as.matrix(LMs$sliders[-80,
    ]), ProcD = FALSE, max.iter = NULL)
```

```
##
## Performing GPA
##   |                                                             |
##
## Making projections... Finished!
```

```
# 2. Procrustes-aligned specimens.
plotAllSpecimens(A = GPA$coords, mean = TRUE)
title(main = "Generalized Procrustes Analysis", col.main = "black",
    font = 2, font.lab = 2, asp = 1)
```

```
dev.off()
```

```
## null device
##           1
```

Experimental GPA

Here we compare obtained Q-values and consensus configurations from different GPA iterations. Anything above 11 iterations starts making the alignment process to behave strange (i.e., the points drift apart). For

reproducibility set seed.

```
num.iter <- 20
Q.res <- matrix(nrow = num.iter, ncol = 2)
colnames(Q.res) <- c("Q.value", "iterations")
for (i in 1:num.iter) {
    pGPA <- gpagen(A = Landmarks, curves = as.matrix(LMs$sliders[-80,
        ]), ProcD = FALSE, max.iter = i)
    Q.res[i, ] <- c(pGPA$Q, pGPA$iter)
}

# Plot results.
plot(Q.res[, 2], Q.res[, 1], xlab = "GPA iterations", ylab = "Q-statistic",
    type = "b", col = "black", pch = 21, lty = 2, font.lab = 2,
    main = "Landmark dataset (p = 160)", ylim = c(0, 7))
# Required iterations to reach convergence
abline(v = 6, lty = 1, lwd = 2, col = "grey50")
# Minimum and maximum Q-value.
abline(h = c(min(Q.res[, 1][1:20]), max(Q.res[, 1][1:20])), col = "red",
    lwd = 2)
points(6, GPA$Q, col = "black", pch = 19, cex = 2)
text(x = 12, y = 4.5, labels = "Iteration-level to convergence",
    font = 2, cex = 1)
```

Digitization measurement error

We re-digitized a random sub-sample (N=30) from our global tooth dataset to compare the accuracy in point placement. Comparability is here assessed using a 1) one-way ANOVA design from which parameter estimates are used to calculate the interclass correlation coefficient [$r = Va/(Va + Vw)$] and 2) a 2B-PLS regression approach. The percentage of measurement error was computed following *Claude (2008)*.

```
# 1. ME landmark data.
ME.TPS <- read2Dtps.noLMs(file = "Measurement Error Analysis (ME)/Measurement Error.TPS",
    ncurve = 2, divide.curve = TRUE, curve.1.pts = 79, curve.2.pts = 81)
# 2. GPA on ME landmark data.
ME.GPA <- gpagen(A = ME.TPS$coords, curves = as.matrix(ME.TPS$sliders[-80,
    ]), ProcD = FALSE)
```

```
##
## Performing GPA
##   |                                                           |
##
## Making projections... Finished!
```

```
# 3. Set difference between original and ME landmark data.
original.LMs <- intersect(dimnames(ME.TPS$coords)[[3]], dimnames(LMs$coords)[[3]])
Set.1 <- LMs$coords[, , original.LMs]
# 4. Re-do GPA on the original landmark data.
Or.GPA <- gpagen(A = Set.1, curves = as.matrix(LMs$sliders[-80,
    ]), ProcD = FALSE)
```

```
##
## Performing GPA
##   |                                                           |
##
## Making projections... Finished!
```

```r
# 5. Compare mean shapes
or.gg <- data.frame(Or.GPA$consensus)
me.gg <- data.frame(ME.GPA$consensus)

# 6. Plot.
u1 <- ggplot(data = or.gg, aes(X, Y)) + geom_point() + coord_fixed()
u2 <- ggplot(data = me.gg, aes(X, Y)) + geom_point() + coord_fixed()
ggarrange(u1, u2, ncol = 2)
```

```r
# 7. Calculate Procrustes distance.
dist.gpa <- procdist(Or.GPA$consensus, ME.GPA$consensus)

# 8. Combine coordinates.
newProc <- rbind(two.d.array(ME.GPA$coords), two.d.array(Or.GPA$coords))
fa <- as.factor(rep(dimnames(ME.GPA$coords)[[3]], 2))
fa <- paste(fa, sep = "")
rep <- as.factor(c(rep("R1", 30), rep("R2", 30)))
# 9. Geomorph data frame.
error.gdf <- geomorph.data.frame(coords = newProc, gp = as.factor(fa),
    rep = factor(rep))
t <- lm.rrpp(f1 = coords ~ gp + rep, iter = 999, RRPP = TRUE,
    SS.type = "II", data = error.gdf)
t.x <- anova(t)
# 10. Intraclass correlation coefficient
expectedwithin <- t.x[[1]][3, 3]  # residual mean sum of squares.
expectedindividual <- (t.x[[1]][1, 3] - expectedwithin)/2  # group sum of squares.
res <- expectedwithin/(expectedwithin + expectedindividual)  # 2% error.
scales::percent(res)
```

```
## [1] "3%"
```

```r
# 11. Pearson product-moment correlation.
cor.test(x = ME.GPA$coords, y = Or.GPA$coords, method = "pearson",
    conf.level = 0.95)
```

```
##
##  Pearson's product-moment correlation
##
## data:  ME.GPA$coords and Or.GPA$coords
## t = 1536.6, df = 9598, p-value < 2.2e-16
## alternative hypothesis: true correlation is not equal to 0
## 95 percent confidence interval:
##  0.9978909 0.9980530
## sample estimates:
##       cor
## 0.9979736
```

```r
# Two.Block Partial Least Squares.
PLS <- two.b.pls(Or.GPA$coords, ME.GPA$coords, iter = 999, print.progress = FALSE)
summary(PLS)
```

```
##
## Call:
## two.b.pls(A1 = Or.GPA$coords, A2 = ME.GPA$coords, iter = 999,
##     print.progress = FALSE)
##
```

```
##
##
## r-PLS: 0.997
##
## Effect Size (Z): 4.6722
##
## P-value: 0.001
##
## Based on 1000 random permutations
```

```
plot(PLS, lwd = 2, col = "black", pch = 19, font.lab = 2)
text(x = 0, y = 0.25, labels = "r-PLS: 0.997, P-value: 0.001, Effect Size: 7.136")
```

Multivariate normality assessment

Henze-Zirkler multivariate normality test. **NB** Load the *MVN* package separately. If the data is multivariate normal, the test statistic HZ is approximately log-normally distributed.

Pre vs. post GPA-alignment of select specimens

A comparison of the configuration shape space vs. Procrustes shape space.

```
layout.matrix <- matrix(c(1, 2), nrow = 1, ncol = 2)
layout(mat = layout.matrix, heights = c(3, 3), widths = c(2,
    2), respect = TRUE)
plot(LMs$coords[, , "Notidanodon-dentatus_Bogan-et-al-2016_2B.jpg"],
    pch = 19, asp = 1, cex = 0.5, col = "#CC3380E6", frame = FALSE,
    xlab = "", ylab = "", main = "Configuration space")
plot(GPA$coords[, , "Notidanodon-dentatus_Bogan-et-al-2016_2B.jpg"],
    pch = 19, asp = 1, cex = 0.5, col = "#CC3380E6", frame = FALSE,
    xlab = "", ylab = "", main = "Procrustes shape space")
```

```
dev.off()
```

```
## null device
##           1
```

Multivariate ordination using PCA

The PCA is performed on the variance-covariance (VCV) matrix of GPA-aligned coordinates using singular value decomposition. PCA results are based on all shape data. That includes both temporally ambiguous (e.g., Danian/Thanetian) and resolved specimens. The former are filtered-out (i.e., omitted) prior to the morphospace and disparity time-series analyses.

```
# 1. Ordination.
PCA <- gm.prcomp(A = GPA$coords)
# 2. Proportion of Variance.
var_exp <- PCA$sdev^2/sum(PCA$sdev^2)
round(var_exp[1:4] * 100, 2)  #  89.28% from 4 PCs.
```

```
## [1] 61.65 12.15 10.83  4.49
```

```
round(var_exp[1:10] * 100, 2) %>%
    sum()  # ~ 96.81% from 10 PCs.
```

```
## [1] 96.69
```

```
# 3. Cumulative Proportion.
cum_pr <- cumsum(PCA$sdev^2/sum(PCA$sdev^2))
# 4. Set PC-axis labels.
xlab <- paste("Principal Component 1 ", "(", round(var_exp[1] *
    100, 1), "%)", sep = "")
ylab <- paste("Principal Component 2 ", "(", round(var_exp[2] *
    100, 1), "%)", sep = "")
```

Non-phylogenetic backtransform morphospace

Procedure adopted after Olsen (2017) to generate backtransform shapes.

```
# Convert array to matrix for PCA.
gpa_mat <- t(apply(GPA$coords, 3, function(y) matrix(t(y), 1)))
# Perform non-phylogenetic PCA.
resEig <- eigen(cov(gpa_mat))
# Get PC scores.
scores <- gpa_mat %*% resEig$vectors
# Get percent variance explained along each axis.
percent.var <- (resEig$values/sum(resEig$values)) * 100
# Define function to draw shape.
plot_tooth_crowns <- function(xy, coor, size = 1, col = "grey90") {
    # Get just x,y coordinates (orthographic projection
    # into xy-plane).
    coor <- coor[, 1:2]
    # Get plot aspect ratio.
    w <- par("pin")[1]/diff(par("usr")[1:2])
    h <- par("pin")[2]/diff(par("usr")[3:4])
    asp <- w/h
    # Correct for plot aspect ratio not necessarily being
    # 1:1.
    coor[, 1] <- coor[, 1] * (1/asp)
    # Scale points and place back in position.
    coor <- coor * size
    # Center about zero based on range of coordinates.
    coor <- coor - matrix(colMeans(apply(coor, 2, range)), nrow = nrow(coor),
        ncol = ncol(coor), byrow = TRUE)
    # Move shape to PC score
    coor <- coor + matrix(xy, nrow(coor), ncol(coor), byrow = TRUE)
    # Create filled polygon
    polygon(coor, col = col, border = gray(0.5))
}
# Set PCs to plot
pcs <- 1:2

# Graphic layout.
layout.matrix <- matrix(c(1, 1, 2, 3), nrow = 2, ncol = 2, byrow = FALSE)
layout(mat = layout.matrix, heights = c(2.5, 2.5), widths = c(4,
    2), respect = TRUE)
par(bg = "white")
# Plot.
plot(scores, type = "n", xlab = paste0("PC", pcs[1], " (", round(percent.var[pcs[1]]),
    "%)"), ylab = paste0("PC", pcs[2], " (", round(percent.var[pcs[2]]),
    "%)"), font.lab = 2, frame = FALSE)
```

```r
abline(h = 0, v = 0, lty = 2, lwd = 2)
title("2D Tooth Shape Morphospace", adj = 0, line = 0.5)

# Plot backtransform shapes.
btShapes(scores = scores, vectors = resEig$vectors, fcn = plot_tooth_crowns,
    pcs = pcs, n = c(5, 7), m = dim(GPA$coords)[2], row.names = dimnames(GPA$coords)[[1]],
    pc.margin = c(0.06, 0.05), size = 0.18, col = gray(0.9))

# Data points by order.
for (i in levels(Data$Order)) {
    # C. vectors for taxonomic grouping.
    order.colours <- c("#E84646", "#377EB8", "darkorange", "purple4",
        "#9D9E9E", "green4", "goldenrod1", "violetred4", "darkcyan")
    names(order.colours) <- levels(Data$Order)
    o.c <- order.colours[match(Data$Order, names(order.colours))]
    points(scores[, 1:2], col = o.c, pch = 19, cex = 0.7)
}

# Box plots corresponding to PC1 and PC2.  S. vector.
sample.size <- c("690", "232", "23", "116", "43", "89", "6",
    "23", "17")
# L. vector.
box.labels <- c("Lamniformes", "Carcharhiniformes", "Heterodontiformes",
    "Orectolobiformes", "Hexanchiformes", "Squaliformes", "Echinorhiniformes",
    "Squatiniformes", "Synechodontiformes")
# PC1.
par(mar = c(6, 4, 1, 1))
boxplot(scores[, 1] ~ Data$Order, col = "white", border = order.colours,
    frame.plot = F, boxwex = 0.8, staplewex = 0.5, boxlwd = 2,
    medlwd = 2, ylab = expression(bold("PC1 (62%)")), xlab = expression(bold("Orders")),
    outcol = scales::alpha(order.colours, 0.5), outpch = 19,
    notch = FALSE, xaxt = "n")
abline(h = mean(scores[, 1]), lwd = 2, col = "black", lty = 2)

# Compute and plot group mean.
uCI <- c(0.0941, 0.0219, -0.1622, -0.0603, -0.1853, -0.1891,
    -0.1402, 0.0143, 0.2216)
iCI <- c(0.062, -0.0246, -0.3187, -0.1163, -0.3062, -0.2489,
    -0.344, -0.0767, -0.0696)
# Alternatively use 'segments' or 'lines'
arrows(1:9, iCI, 1:9, uCI, lwd = 2, angle = 90, code = 3, length = 0.05,
    col = "black")
points(tapply(scores[, 1], Data$Order, mean), pch = 21, cex = 1.5,
    bg = "white", col = "black")
# PC2.
boxplot(scores[, 2] ~ Data$Order, col = "white", border = order.colours,
    frame.plot = F, xaxt = "n", boxwex = 0.8, staplewex = 0.5,
    boxlwd = 2, medlwd = 2, ylab = expression(bold("PC2 (12%)")),
    xlab = "", outcol = scales::alpha(order.colours, 0.5), outpch = 19,
    font.lab = 2)
abline(h = mean(scores[, 2]), lwd = 2, col = "black", lty = 2)

# Compute and plot group mean.
```

```
PC2.uCI <- c(0.0036, 0.0137, 0.0735, 0.0415, 0.1742, -0.0457,
    0.0518, 0.0649, 0.0899)
PC2.iCI <- c(-0.0097, -0.0128, 0.0182, 0.0132, 0.104, -0.0872,
    -0.2972, -0.0037, -0.0254)
arrows(1:9, PC2.iCI, 1:9, PC2.uCI, lwd = 2, angle = 90, code = 3,
    length = 0.05, col = "black")
points(tapply(scores[, 2], Data$Order, mean), pch = 21, cex = 1.5,
    bg = "white", col = "black")

# Add text labels for groups on x-axis.
axis(1, at = 1:9, labels = FALSE, tck = -0.02)
text(x = 1:9, y = par()$usr[3] - 0.025 * (par()$usr[4] - par()$usr[3]),
    labels = box.labels, srt = 45, adj = 1, xpd = TRUE)
# Add text labels for sample sizes.
mtext("Sample size (N)", side = 3, line = 2, cex = 0.85, col = "black",
    font = 2)
mtext(sample.size, at = c(1:9), side = 3, line = 1, cex = 0.5,
    col = "black")
```

```
dev.off()
```

```
## null device
##           1
```

```
# 1. Swap axes to match eigenvector direction of bt-PCA.
PCA$x[, 1:2] <- PCA$x[, 1:2] * -1
PCA$x[, 4] <- PCA$x[, 4] * -1
# 2. Check that scores from btPCA and geomorph PCA are
# consistent.
par(mfrow = c(2, 2))
plot(scores[, 1] ~ PCA$x[, 1])
plot(scores[, 2] ~ PCA$x[, 2])
plot(scores[, 3] ~ PCA$x[, 3])
plot(scores[, 4] ~ PCA$x[, 4])
```

```
dev.off()
```

```
## null device
##           1
```

Screeplot showing the distribution of eigenvalues

```
layout.matrix <- matrix(c(1, 2), nrow = 1, ncol = 2, byrow = FALSE)
layout(mat = layout.matrix, heights = c(6, 6), widths = c(5,
    3), respect = TRUE)
# Proportion of Variance.
barplot(var_exp[1:10]/1, space = 0.05, beside = TRUE, horiz = FALSE,
    main = expression(paste("Distribution of eigenvalues" ~ (lambda[k]))),
    font.main = 4, las = 1, xpd = FALSE, xlab = "Principal Components",
    ylab = "Percentage of variances", col = "grey70", border = "black",
    font.lab = 2)
# Add connected line segments to the plot.
lines(var_exp[1:10]/1, type = "b", pch = 19, col = scales::alpha("purple",
    0.5), cex = 1.5)
abline(h = mean(var_exp[1:10]), lwd = 2, lty = "dashed", col = "black")
```

```r
legend(x = 2, y = 0.4, lty = 2, legend = "Average eigenvalue",
    lwd = 2, bty = "n")
# Cumulative Proportion.
plot(cum_pr[1:10], type = "o", pch = 21, main = "Cumulative Proportion",
    lty = 1, lwd = 2, xlab = "Principal Components", ylab = "",
    col = "black", bg = "grey70", font.lab = 2, bty = "n")
```

```r
dev.off()
```

```
## null device
##           1
```

Extreme shapes

Determine the maximum and minimum range for select axes based on the pc-scores.

```r
PC1min <- GPA$consensus + matrix(-0.5208907 * (resEig$vectors[,
    1]), byrow = T, 160, 2)
PC1max <- GPA$consensus + matrix(0.5485006 * (resEig$vectors[,
    1]), byrow = T, 160, 2)
PC2min <- GPA$consensus + matrix(-0.3451388 * (resEig$vectors[,
    2]), byrow = T, 160, 2)
PC2max <- GPA$consensus + matrix(0.3041162 * (resEig$vectors[,
    2]), byrow = T, 160, 2)
PC3min <- GPA$consensus + matrix(-0.2921642 * (resEig$vectors[,
    3]), byrow = T, 160, 2)
PC3max <- GPA$consensus + matrix(0.2830423 * (resEig$vectors[,
    3]), byrow = T, 160, 2)
PC4min <- GPA$consensus + matrix(-0.3153873 * (resEig$vectors[,
    4]), byrow = T, 160, 2)
PC4max <- GPA$consensus + matrix(0.1612285 * (resEig$vectors[,
    4]), byrow = T, 160, 2)
```

Shape visualization with Morpho

```r
layout(matrix(c(1:4), nrow = 2, ncol = 2, byrow = T), respect = T)
deformGrid2d(PC1min, PC1min, pch = 19, cex1 = 1, cex2 = 1, lwd = 1,
    lines = F, col1 = "#AA1936", col2 = "#AA1936")
mtext(text = "PC1min", side = 1, line = 2)
deformGrid2d(PC1max, PC1max, pch = 19, cex1 = 1, cex2 = 1, lwd = 1,
    lines = F, col1 = "#AA1936", col2 = "#AA1936")
mtext(text = "PC1max", side = 1, line = 2)
deformGrid2d(PC2min, PC2min, pch = 19, cex1 = 1, cex2 = 1, lwd = 1,
    lines = F, col1 = "#AA1936", col2 = "#AA1936")
mtext(text = "PC2min", side = 1, line = 2)
deformGrid2d(PC2max, PC2max, pch = 19, cex1 = 1, cex2 = 1, lwd = 1,
    lines = F, col1 = "#AA1936", col2 = "#AA1936")
mtext(text = "PC2max", side = 1, line = 2)
```

```r
dev.off()
```

```
## null device
##           1
```

Shape visualization with Momocs

- Deformation isolines using Thin Plate Splines. The TPS-formalism is similar for the semilandmark algorithm (Mitteroecker & Guns 2009).

```
heat_col <- sequential_hcl(15, "Heat")
par(mfcol = c(2, 2), mar = c(4, 4, 4, 4), oma = c(1.5, 2, 1,
    1))
tps_iso(PC1max, PC1min, poly = F, iso.nb = 200, amp = 0.05, shp = T,
    cont = T, grid = TRUE, shp.border = c("white", "black"),
    shp.lwd = c(2, 2), legend = TRUE, legend.text = c("maximum",
        "minimum"), palette = colorRampPalette(heat_col))
title(main = "Principal component 1 (62%)")
tps_iso(PC2min, PC2max, poly = F, iso.nb = 200, amp = 0.05, shp = T,
    cont = T, grid = TRUE, shp.border = c("white", "black"),
    shp.lwd = c(2, 2), legend = TRUE, legend.text = c("maximum",
        "minimum"), palette = colorRampPalette(heat_col))
title(main = "Principal component 2 (12%)")
tps_iso(PC3min, PC3max, poly = F, iso.nb = 200, amp = 0.05, shp = T,
    cont = T, grid = TRUE, shp.border = c("white", "black"),
    shp.lwd = c(2, 2), legend = TRUE, legend.text = c("maximum",
        "minimum"), palette = colorRampPalette(heat_col))
title(main = "Principal component 3 (11%)")
tps_iso(PC4min, PC4max, poly = F, iso.nb = 200, amp = 0.05, shp = T,
    cont = T, grid = TRUE, shp.border = c("white", "black"),
    shp.lwd = c(2, 2), legend = TRUE, legend.text = c("maximum",
        "minimum"), palette = colorRampPalette(heat_col))
title(main = "Principal component 4 (5%)")
mtext(text = "Deformation isolines using Thin Plate Splines",
    outer = TRUE, cex = 1, font = 2)
```

```
dev.off()
```

```
## null device
##           1
```

Deformation 'vector fields' using Thin Plate Splines

```
par(mfrow = c(1, 2))
tps_arr(GPA$consensus, PCA$shapes$shapes.comp1$min, arr.nb = 100,
    palette = col_sari, amp = 1e-04, grid = T, over = 1.5, poly = F,
    shp = T, shp.lwd = 5, legend = T, shp.col = "maroon", arr.lwd = 2)
tps_arr(fr = PCA$shapes$shapes.comp1$min, to = PCA$shapes$shapes.comp1$max,
    arr.nb = 70, arr.levels = 2, palette = pal_qual_solarized,
    amp = 0.1, grid = T, over = 1.5, poly = F, shp = T, shp.col = c("#338080E6",
        "#CC3380E6"), shp.lwd = c(5, 5), shp.lty = c(1, 1), legend = F,
    arr.lwd = 2, shp.border = col_qual(2))
```

```
dev.off()
```

```
## null device
##           1
```

Analysis of variance (ANOVA)

The test-statistics presented here are based on the total data space irrespective of temporal affiliation. The null expectation is that groups (i.e., shark orders) do not occupy significantly different areas of morphospace. To obtain axis specific results re-run the linear model **(Proc.aov)** with the desired axis (e.g. PC1). All p-values from the pairwise test have been FDR-adjusted.

Diagnostic plots

Procrustes residuals from the linear model fit.

```
par(mfrow = c(2, 2))
plot(Proc.aov, type = "diagnostics", outliers = TRUE)
```

```
dev.off()
```

```
## null device
##           1
```

Morphospace time-series analysis

This section explores temporal patterns in total-shark group (i.e. Selachimorpha) across the K/Pg boundary using the global five-stage and four-stage time binning schemes along major axes of variation. To obtain results for PC3 and PC4 replace the axis value inside the relevant data frame below.

```
# 1. Create data frames.
FiveBin.Sel <- data.frame(pc1 = scores[, 1], pc2 = scores[, 2],
    groups = Data$Clade, age = Data$Age)
FourBin.Sel <- data.frame(pc1 = scores[, 1], pc2 = scores[, 2],
    groups = Data$Clade, age = Data$Age.3)
# 2. Arrange time-bins.
FiveBin.Sel$age <- factor(FiveBin.Sel$age, levels = c("Campanian",
    "Maastrichtian", "Danian", "Selandian", "Thanetian"))
FourBin.Sel$age <- factor(FourBin.Sel$age, levels = c("Campanian",
    "Maastrichtian", "DanSelCombined", "Thanetian"))
# 3. Molten data frame.
molten5.df <- melt(FiveBin.Sel)
```

```
## Using groups, age as id variables
```

```
molten4.df <- melt(FourBin.Sel)
```

```
## Using groups, age as id variables
```

```
# 4. Five-stage time binning scheme.
ggplot(na.omit(molten5.df), aes(x = age, y = value, fill = groups)) +
    geom_point(position = position_jitterdodge(dodge.width = 0.5),
        alpha = 0.5) + geom_boxplot(outlier.size = 3, alpha = 0.2,
    fill = "white", colour = "black") + stat_summary(fun.data = mean_cl_boot,
    geom = "errorbar", width = 0.1, lwd = 1, linetype = 1, col = "#CC3380E6") +
    stat_summary(fun = "mean", geom = "point", lwd = 2, position = position_dodge(width = 1),
        color = "#CC3380E6", pch = 21) + stat_summary(fun = "estimate_mode",
    geom = "point", col = "#4D897C", pch = 19) + facet_wrap(. ~
    variable, nrow = 2, ncol = 1, scales = "free_y") + labs(x = "Age",
    y = "Principal Component") + theme_grey() + theme(axis.title = element_text(color = "#666666",
    face = "bold", size = 8), axis.text.y = element_text(angle = 90,
    size = 8), axis.text.x = element_text(angle = 45, vjust = 0.5,
    hjust = 0.75, size = 8), axis.line = element_line(linetype = "solid"),
```

```
    legend.position = "none", panel.spacing = unit(0, "lines"),
    aspect.ratio = 2/4)
```

```
# 5. Four-stage time binning scheme.
ggplot(na.omit(molten4.df), aes(x = age, y = value, fill = groups)) +
    geom_point(position = position_jitterdodge(dodge.width = 0.5),
        alpha = 0.5) + geom_boxplot(outlier.size = 3, alpha = 0.2,
    fill = "white", colour = "black") + stat_summary(fun.data = mean_cl_boot,
    geom = "errorbar", width = 0.1, lwd = 1, linetype = 1, col = "#CC3380E6") +
    stat_summary(fun = "mean", geom = "point", lwd = 2, position = position_dodge(width = 1),
        color = "#CC3380E6", pch = 21) + stat_summary(fun = "estimate_mode",
    geom = "point", col = "#4D897C", pch = 19) + facet_wrap(. ~
    variable, nrow = 2, ncol = 1, scales = "free_y") + labs(x = "Age",
    y = "Principal Component") + theme_grey() + theme(axis.title = element_text(color = "#666666",
    face = "bold", size = 8), axis.text.y = element_text(angle = 90,
    size = 8), axis.text.x = element_text(angle = 45, vjust = 0.5,
    hjust = 0.75, size = 8), axis.line = element_line(linetype = "solid"),
    legend.position = "none", panel.spacing = unit(0, "lines"),
    aspect.ratio = 2/4)
```

Modal computation and visualization

Compute the modal-value for each age from the first 4 axes of variation. Positive-values on PC1 are broad teeth and negative values are narrow teeth **(see TPS Grids)**. We are losing some of the cusplet geometry on this axis. The crown length decrease and the tooth size enlarges.

```
# Re-level factor.
Data$Age <- factor(Data$Age, levels = c("Campanian", "Maastrichtian",
    "Danian", "Selandian", "Thanetian"))
pc.axes <- c("PC1", "PC2", "PC3", "PC4")
ages <- c(1:5)
mode.value <- matrix(nrow = 5, ncol = 4, dimnames = list(ages,
    pc.axes), byrow = T)
for (i in 1:length(ages)) {
    axes <- 1:4
    for (j in 1:length(axes)) {
        res <- estimate_mode(scores[Data$Age == levels(Data$Age)[ages[i]],
            axes[j]])
        mode.value[[i, j]] <- res
    }
}

# Modal-shape along PC1.
CMDPC1 <- GPA$consensus + matrix(-0.15 * (resEig$vectors[, 1]),
    byrow = T, 160, 2)
SPC1 <- GPA$consensus + matrix(-0.05 * (resEig$vectors[, 1]),
    byrow = T, 160, 2)
TPC1 <- GPA$consensus + matrix(0.05 * (resEig$vectors[, 1]),
    byrow = T, 160, 2)
# Modal-shape along PC2.
CPC2 <- GPA$consensus + matrix(0.025 * (resEig$vectors[, 2]),
    byrow = T, 160, 2)
MPC2 <- GPA$consensus + matrix(-0.075 * (resEig$vectors[, 2]),
    byrow = T, 160, 2)
```

```r
DPC2 <- GPA$consensus + matrix(0.075 * (resEig$vectors[, 2]),
    byrow = T, 160, 2)
SPC2 <- GPA$consensus + matrix(0.075 * (resEig$vectors[, 2]),
    byrow = T, 160, 2)
TPC2 <- GPA$consensus + matrix(-0.025 * (resEig$vectors[, 2]),
    byrow = T, 160, 2)
# Modal-shape along PC3.
CPC3 <- GPA$consensus + matrix(-0.075 * (resEig$vectors[, 3]),
    byrow = T, 160, 2)
MPC3 <- GPA$consensus + matrix(-0.025 * (resEig$vectors[, 3]),
    byrow = T, 160, 2)
DPC3 <- GPA$consensus + matrix(-0.025 * (resEig$vectors[, 3]),
    byrow = T, 160, 2)
SPC3 <- GPA$consensus + matrix(-0.125 * (resEig$vectors[, 3]),
    byrow = T, 160, 2)
TPC3 <- GPA$consensus + matrix(0.025 * (resEig$vectors[, 3]),
    byrow = T, 160, 2)
# Modal-shape along PC3.
CPC3 <- GPA$consensus + matrix(-0.075 * (resEig$vectors[, 3]),
    byrow = T, 160, 2)
MPC3 <- GPA$consensus + matrix(-0.025 * (resEig$vectors[, 3]),
    byrow = T, 160, 2)
DPC3 <- GPA$consensus + matrix(-0.025 * (resEig$vectors[, 3]),
    byrow = T, 160, 2)
SPC3 <- GPA$consensus + matrix(-0.125 * (resEig$vectors[, 3]),
    byrow = T, 160, 2)
TPC3 <- GPA$consensus + matrix(0.025 * (resEig$vectors[, 3]),
    byrow = T, 160, 2)
# Modal-shape along PC4.
CMDTPC4 <- GPA$consensus + matrix(0.025 * (resEig$vectors[, 4]),
    byrow = T, 160, 2)
SPC4 <- GPA$consensus + matrix(-0.075 * (resEig$vectors[, 4]),
    byrow = T, 160, 2)
```

Modal Shapes

```r
# Plot grids.
layout(matrix(1:3, nrow = 1, ncol = 3))
tps_grid(CMDPC1, CMDPC1, grid.size = 10, shp = T, legend = T,
    shp.lwd = 6, legend.text = "Modal Shape For Cmp-Maa-Dan on PC1")
tps_grid(SPC1, SPC1, grid.size = 10, shp = T, legend = T, shp.lwd = 6,
    legend.text = "Modal Shape For Sel on PC1")
tps_grid(TPC1, TPC1, grid.size = 10, shp = T, legend = T, shp.lwd = 6,
    legend.text = "Modal Shape For Tha on PC1")
```

```r
dev.off()
```

```
## null device
##           1
```

```r
layout(matrix(1:5, nrow = 1, ncol = 5))
tps_grid(CPC2, CPC2, grid.size = 10, shp = T, legend = T, shp.lwd = 6,
    legend.text = "Modal Shape For Cmp on PC2")
tps_grid(MPC2, MPC2, grid.size = 10, shp = T, legend = T, shp.lwd = 6,
```

```
    legend.text = "Modal Shape For Maa on PC2")
tps_grid(DPC2, DPC2, grid.size = 10, shp = T, legend = T, shp.lwd = 6,
    legend.text = "Modal Shape For Dan on PC2")
tps_grid(SPC2, SPC2, grid.size = 10, shp = T, legend = T, shp.lwd = 6,
    legend.text = "Modal Shape For Sel on PC2")
tps_grid(TPC2, TPC2, grid.size = 10, shp = T, legend = T, shp.lwd = 6,
    legend.text = "Modal Shape For Tha on PC2")
dev.off()
```

```
## null device
##           1
```

```
layout(matrix(1:5, nrow = 1, ncol = 5))
tps_grid(CPC3, CPC3, grid.size = 10, shp = T, legend = T, shp.lwd = 6,
    legend.text = "Modal Shape For Cmp on PC3")
tps_grid(MPC3, MPC3, grid.size = 10, shp = T, legend = T, shp.lwd = 6,
    legend.text = "Modal Shape For Maa on PC3")
tps_grid(DPC3, DPC3, grid.size = 10, shp = T, legend = T, shp.lwd = 6,
    legend.text = "Modal Shape For Dan on PC3")
tps_grid(SPC3, SPC3, grid.size = 10, shp = T, legend = T, shp.lwd = 6,
    legend.text = "Modal Shape For Sel on PC3")
tps_grid(TPC3, TPC3, grid.size = 10, shp = T, legend = T, shp.lwd = 6,
    legend.text = "Modal Shape For Tha on PC3")

layout(matrix(1:2, nrow = 1, ncol = 2))
tps_grid(CMDTPC4, CMDTPC4, grid.size = 10, shp = T, legend = T,
    shp.lwd = 6, legend.text = "Modal Shape For Cmp-Maa-Dan-Tha on PC4")
tps_grid(SPC4, SPC4, grid.size = 10, shp = T, legend = T, shp.lwd = 6,
    legend.text = "Modal Shape For Sel on PC4")
dev.off()
```

```
## null device
##           1
```

Age and group specific data frames

```
# 1. Main time-binning schemes for total-clade
# Selachimorpha.
Five.Df <- Data[Data$Age %in% c("Campanian", "Maastrichtian",
    "Danian", "Selandian", "Thanetian"), ]
Four.Df <- Data[Data$Age.3 %in% c("Campanian", "Maastrichtian",
    "DanSelCombined", "Thanetian"), ]
Three.Df <- Data[Data$Country %in% "Denmark", ]
# 2. Group-specific data frames.
Galeo.Df <- Data[Data$Superorder %in% "Galeomorphii", ]
Galeo.Df <- Galeo.Df[Galeo.Df$Age.3 %in% c("Campanian", "Maastrichtian",
    "DanSelCombined", "Thanetian"), ]
Squali.Df <- Data[Data$Superorder %in% "Squalomorphii", ]
Squali.Df <- Squali.Df[Squali.Df$Age.3 %in% c("Campanian", "Maastrichtian",
    "DanSelCombined", "Thanetian"), ]
```

```
geo.ages <- c("Campanian", "Maastrichtian", "DanSelCombined",
    "Thanetian")
```

Time series linear model evaluation

Anova statistics applied to principal component axes. Differences between group LS-means (i.e., time-bins) are examined using RRPP. Type I SS "sequential" are used. All final p-values are corrected using FDR-adjustment. Replace the f1 argument to access axes-specific results.

```
# 1. Time-bin specific data frames.

# Geomorph data frames.  Global five stage time-binning
# scheme: N = 1156 specimens.
Sel.5.Bin <- geomorph.data.frame(pcs = scores[rownames(Five.Df),
    ], pc1 = scores[rownames(Five.Df), 1], pc2 = scores[rownames(Five.Df),
    2], pc3 = scores[rownames(Five.Df), 3], pc4 = scores[rownames(Five.Df),
    4], ages = factor(Five.Df$Age))
# Global four stage time-binning scheme: N = 1198
# specimens.
Sel.4.Bin <- geomorph.data.frame(pcs = scores[rownames(Four.Df),
    ], pc1 = scores[rownames(Four.Df), 1], pc2 = scores[rownames(Four.Df),
    2], pc3 = scores[rownames(Four.Df), 3], pc4 = scores[rownames(Four.Df),
    4], ages = factor(Four.Df$Age.3))
# Regional three stage time-binning scheme: N = 153
# specimens.
Sel.3.Bin <- geomorph.data.frame(pcs = scores[rownames(Three.Df),
    ], pc1 = scores[rownames(Three.Df), 1], pc2 = scores[rownames(Three.Df),
    2], pc3 = scores[rownames(Three.Df), 3], pc4 = scores[rownames(Three.Df),
    4], ages = factor(Three.Df$Sub))

# 2. Selachimorpha 5-bin:
Sel.5.Bin.aov <- lm.rrpp(f1 = pcs ~ ages, iter = 999, RRPP = TRUE,
    SS.type = "I", data = Sel.5.Bin)
anova(Sel.5.Bin.aov)
# 3. Selachimorpha 4-bin.
Sel.4.Bin.aov <- lm.rrpp(f1 = pcs ~ ages, iter = 999, RRPP = TRUE,
    SS.type = "I", data = Sel.4.Bin)
anova(Sel.4.Bin.aov)
# 4. Selachimorpha 3-bin: regional sub-sample.
Sel.3.Bin.aov <- lm.rrpp(f1 = pcs ~ ages, iter = 999, RRPP = TRUE,
    SS.type = "I", data = Sel.3.Bin)
anova(Sel.3.Bin.aov)

# 5. Pairwise statistics.
PW.lm <- pairwise(fit = Sel.5.Bin.aov, groups = interaction(Sel.5.Bin$ages))
# 6. Character.
age.attr.a <- c("Campanian", "Danian", "Maastrichtian", "Selandian",
    "Thanetian")
age.attr.b <- c("Campanian", "DanSelCombined", "Maastrichtian",
    "Thanetian")
age.attr.c <- c("early", "late", "middle")
# 7. Pairwise distances between means.
sPW <- summary.pairwise(PW.lm, test.type = "dist", confidence = 0.95,
    stat.table = TRUE)
# 8. P-value adjustment.
round(matrix(p.adjust(sPW$pairwise.tables$P, method = "fdr"),
    nrow = 5, dimnames = list(age.attr.a, age.attr.a)), 3)
```

Continuation of model fitting

Anova statistics is calculated on the basis of comparing age differences among superorders namely Galeomorphii and Squalomorphii. The following analysis is based on the global four-stage time binning scheme and regional three-stage time binning scheme. We have included Synechodontiformes here as part of Galeomorphii.

```
# 1. Galeomorphii: N = 1043 specimens.
Gal.4.Bin <- geomorph.data.frame(pcs = scores[rownames(Galeo.Df),
    ], pc1 = scores[rownames(Galeo.Df), 1], pc2 = scores[rownames(Galeo.Df),
    2], pc3 = scores[rownames(Galeo.Df), 3], pc4 = scores[rownames(Galeo.Df),
    4], ages = factor(Galeo.Df$Age.3))
# 2. Squalomorphii: N = 155 specimens.
Squ.4.Bin <- geomorph.data.frame(pcs = scores[rownames(Squali.Df),
    ], pc1 = scores[rownames(Squali.Df), 1], pc2 = scores[rownames(Squali.Df),
    2], pc3 = scores[rownames(Squali.Df), 3], pc4 = scores[rownames(Squali.Df),
    4], ages = factor(Squali.Df$Age.3))

# 3. Galeomorphii 4-bin.
Gal.aov <- lm.rrpp(f1 = pcs ~ ages, iter = 999, RRPP = TRUE,
    SS.type = "I", data = Gal.4.Bin)
anova(Gal.aov)
# 4. Squalomorphii 4-bin.
Squ.aov <- lm.rrpp(f1 = pcs ~ ages, iter = 999, RRPP = TRUE,
    SS.type = "I", data = Squ.4.Bin)
anova(Squ.aov)
# 5. Pairwise statistics.
super.PW <- pairwise(fit = Squ.aov, groups = interaction(Squ.4.Bin$ages))
# 6. Pairwise distances between means.
sp.PW <- summary.pairwise(super.PW, test.type = "dist", confidence = 0.95,
    stat.table = TRUE)
# 7. P-value adjustment.
round(matrix(p.adjust(sp.PW$pairwise.tables$P, method = "fdr"),
    nrow = 4, ncol = 4, dimnames = list(age.attr.b, age.attr.b)),
    3)
```

Regional shark morphospace

Regional sub-sample: Stevns Klint, Denmark (N=153). Sub-stages include the late Maastrichtian, early and middle Danian.

```
# 1. Data frame.
Denmark.Df <- data.frame(scores = scores[rownames(Three.Df),
    1:2], groups = factor(Three.Df$Clade), age = factor(Three.Df$Sub))
# 2. Arrange time-bins.
Denmark.Df$age <- factor(Denmark.Df$age, levels = c("late", "early",
    "middle"))
# 3. Melt.
melt.Reg <- melt(Denmark.Df)
```

```
## Using groups, age as id variables
```

```
# 4. Plot-
ggplot(na.omit(melt.Reg), aes(x = age, y = value, fill = groups)) +
    geom_point(position = position_jitterdodge(dodge.width = 0.5),
        alpha = 0.5) + geom_boxplot(width = 0.5, outlier.size = 3,
    alpha = 0.2, fill = "white", colour = "black") + stat_summary(fun.data = mean_cl_boot,
```

```
    geom = "errorbar", width = 0.1, lwd = 1, linetype = 1, col = "#CC3380E6") +
    stat_summary(fun = "mean", geom = "point", lwd = 2, position = position_dodge(width = 1),
        color = "#CC3380E6", pch = 21) + stat_summary(fun = "estimate_mode",
    geom = "point", col = "#4D897C", pch = 19) + facet_wrap(. ~
    variable, nrow = 2, ncol = 1, scales = "free_y") + labs(x = "Age",
    y = "Principal Component") + theme_grey() + theme(axis.title = element_text(color = "#666666",
    face = "bold", size = 8), axis.text.y = element_text(angle = 90,
    size = 8), axis.text.x = element_text(angle = 45, vjust = 0.5,
    hjust = 0.75, size = 8), axis.line = element_line(linetype = "solid"),
    legend.position = "none", panel.spacing = unit(0, "lines"),
    aspect.ratio = 3/4)
```

Superorder morphospace through time

Temporal visualization along PC1-PC2. The following plot shows temporal variation in Galeomorphii and Squalomorphii across the K/Pg boundary using the global four-stage time binning scheme.

```
# Data frame.
ggPCA.time <- data.frame(scores = scores[, 1:2], groups = factor(Data$Superorder),
    age = factor(Data$Age.3))
# Re-level categories.
ggPCA.time$age = factor(ggPCA.time$age, levels = geo.ages)
# Molten data frame.
SO.melt <- melt(ggPCA.time)
```

```
## Using groups, age as id variables
```

```
SO.melt$groups <- factor(SO.melt$groups, c("Galeomorphii", "Squalomorphii"))
# Time series.
ggplot(na.omit(SO.melt), aes(x = groups, y = value, fill = groups,
    colour = groups)) + geom_boxplot(varwidth = F, outlier.size = 3,
    alpha = 0.2, fill = "white") + geom_point(position = position_jitterdodge(dodge.width = 0.9),
    alpha = 0.5, size = 0.2) + stat_summary(fun.data = mean_cl_boot,
    geom = "errorbar", width = 0.25, lwd = 0.5, linetype = 1,
    col = "black") + stat_summary(fun = "mean", geom = "point",
    lwd = 1, position = position_dodge(width = 0.25), bg = "white",
    color = "black", pch = 21) + scale_fill_manual(values = c("#233B43",
    "#A76281", "#61A375")) + scale_color_manual(values = c("#233B43",
    "#A76281", "#61A375")) + facet_grid(variable ~ age, scales = "free",
    margins = FALSE) + labs(x = "Time (Ma)", y = "PC") + theme_classic() +
    theme(legend.position = "right") + theme(axis.title = element_text(color = "#666666",
    face = "bold", size = 12)) + theme(axis.title.x = element_blank(),
    axis.text.x = element_blank(), axis.ticks.x = element_blank(),
    legend.position = "none", aspect.ratio = 1.5)
```

Selachimorpha sub-clade (order) morphospace

Echinorhiniformes is removed from the analysis because of insufficient sample size (N=6). The following plot shows temporal variation in all shark clades across the K/Pg boundary using the global four-stage time binning scheme.

```
# 1. Characters.
orders <- c("Lamniformes", "Carcharhiniformes", "Heterodontiformes",
    "Orectolobiformes", "Hexanchiformes", "Squaliformes", "Squatiniformes",
    "Synechodontiformes")
```

```r
# 2. Data frame.
clade.Df <- Data[Data$Order %in% orders, ]
clade.Df$Order <- factor(clade.Df$Order)

# 3. Create data frame.
ggdf.1 <- data.frame(scores = scores[rownames(clade.Df), 1:2],
    groups = factor(clade.Df$Order), age = factor(clade.Df$Age.3))
# 4. Arrange time-bins.
ggdf.1$age <- factor(ggdf.1$age, levels = geo.ages)
# 5. Re-order the levels in the order of appearance in the
# data.frame:
ggdf.1$groups <- factor(ggdf.1$groups, orders)
# 6. Melt.
melt.p1 <- melt(ggdf.1)
```

```
## Using groups, age as id variables
```

```r
# 7. Plot.
ggplot(na.omit(melt.p1), aes(x = age, y = value, fill = groups)) +
    geom_boxplot(outlier.colour = "grey", alpha = 0.45) + stat_summary(fun.data = mean_cl_boot,
    geom = "errorbar", width = 0.25, lwd = 0.5, linetype = 1,
    col = "black") + stat_summary(fun = "mean", geom = "point",
    lwd = 1, position = position_dodge(width = 0.25), bg = "grey",
    color = "black", pch = 21) + stat_summary(fun = mean, geom = "smooth",
    aes(group = 1), lwd = 1, col = "black") + scale_fill_manual(values = c("#E84646",
    "#377EB8", "darkorange", "purple4", "black", "green4", "violetred4",
    "darkcyan")) + stat_summary(fun = "estimate_mode", geom = "point",
    col = "blue", pch = 19) + facet_grid(variable ~ groups, scales = "free",
    margins = FALSE) + labs(x = "Age (Ma)", y = "Principal Component") +
    theme_classic() + theme(legend.position = "none") + theme(axis.title = element_text(color = "#66666
    face = "bold", size = 12)) + theme(axis.title.x = element_blank(),
    axis.text.x = element_blank(), axis.ticks.x = element_blank())
```

Select family-level morphospace

```r
# Prepare color.
colors_border <- c(rgb(0.2, 0.5, 0.5, 0.9), rgb(0.8, 0.2, 0.5,
    0.9))
# Data frame: Maastrichtian and early Paleocene Anacoracids
# and Hexchanids.
Maas.Dan <- Data[Data$Age.3 %in% c("Maastrichtian", "DanSelCombined"),
    ]
fams <- which(Maas.Dan$Family == "Anacoracidae" | Maas.Dan$Order ==
    "Hexanchiformes")
AnaHex <- Maas.Dan[fams, ]
# Data frame.
ggdf.2 <- data.frame(pc1 = scores[rownames(AnaHex), 1], pc2 = scores[rownames(AnaHex),
    2], groups = factor(AnaHex$Family), age = factor(AnaHex$Age.3))

melt.p2 <- melt(ggdf.2)
```

```
## Using groups, age as id variables
```

```
# PLot.
ggplot(melt.p2, aes(x = value, color = groups)) + geom_boxplot() +
    scale_fill_manual(values = c("#E84646", "black", "blue",
        "green", "purple")) + scale_color_manual(values = c("#E84646",
    "black", "blue", "green", "purple")) + facet_grid(age ~ variable,
    scales = "free", space = "free_x") + theme(aspect.ratio = 1/3)
```

IQR's

```
# Orectolobiformes.
IQR(PCA$x[which(Orect.Df$Age.3 == "Maastrichtian"),2])
IQR(PCA$x[which(Orect.Df$Age.3 == "DanSelCombined"),2])
# Heterodontiformes
IQR(PCA$x[which(Orect.Df$Age.3 == "Maastrichtian"),2])
IQR(PCA$x[which(Orect.Df$Age.3 == "DanSelCombined"),2])
```

Anova statistics applied to shark subclades

```
anova.test(scores = scores[Data$Order == "Lamniformes", ], bins = Data$Age[Data$Order ==
    "Lamniformes"], axes = 1:4)
anova.test(scores = scores[Data$Order == "Carcharhiniformes",
    ], bins = Data$Age[Data$Order == "Carcharhiniformes"], axes = 1:4)
anova.test(scores = scores[Data$Order == "Heterodontiformes",
    ], bins = Data$Age[Data$Order == "Heterodontiformes"], axes = 1:4)
anova.test(scores = scores[Data$Order == "Orectolobiformes",
    ], bins = Data$Age[Data$Order == "Orectolobiformes"], axes = 1:4)
anova.test(scores = scores[Data$Order == "Hexanchiformes", ],
    bins = Data$Age[Data$Order == "Hexanchiformes"], axes = 1:4)
anova.test(scores = scores[Data$Order == "Squaliformes", ], bins = Data$Age[Data$Order ==
    "Squaliformes"], axes = 1:4)
anova.test(scores = scores[Data$Order == "Squatiniformes", ],
    bins = Data$Age[Data$Order == "Squatiniformes"], axes = 1:4)
anova.test(scores = scores[Data$Order == "Synechodontiformes",
    ], bins = Data$Age[Data$Order == "Synechodontiformes"], axes = 1:4)
```

Procrustes variance and partial disparity analyses

Here we compute the disparity (PV) of clades (e.g., Superorder,Order,Family) at different time-bins using a subsampling-based approach (i.e., rarefaction). Pseudoreplicate data is generated by re-sampling. **Note:** The time to run all disparity analyses with bootstrap statistics (while it may vary between computer processors) will take ~4 weeks.

```
# 1a. Global-level analysis of total Selachimorpha using
# the five-stage time binning scheme.  Sample size = 1156
Disp.A <- error.plot(gpa.coords = GPA$coords[, , rownames(Five.Df)],
    blank = FALSE, groups = Five.Df$Age, order = c(1, 2, 3, 4,
        5), replicates = 999, rarefy.par = list(min.N = 28, reps = 999))

# 1b.Permutations re-sampling procedure test.
Perm.S5.Bin <- disparity.calc(gpa = GPA, data = Data, ages = "Age")
Perm.S5.Bin$Procrustes.var[c(1, 3, 2, 4, 5)]
Perm.S5.Bin$PV.dist.Pval
# Adjust p-value.
round(matrix(p.adjust(Perm.S5.Bin$PV.dist.Pval, method = "fdr"),
```

```r
    nrow = 5, dimnames = list(age.attr.a, age.attr.a)), 3)
```

```r
# 2a. Global-level analysis of total Selachimorpha using
# the four-stage time binning scheme.  Sample size = 1198
Disp.B <- error.plot(gpa.coords = GPA$coords[, , rownames(Four.Df)],
    blank = FALSE, groups = Four.Df$Age.3, order = c(1, 3, 2,
        4), replicates = 999, rarefy.par = list(min.N = 187,
        reps = 999))

# 2b.Permutations re-sampling procedure test.
Perm.S4.Bin <- disparity.calc(gpa = GPA, data = Data, ages = "Age.3")
Perm.S4.Bin$Procrustes.var[c(1, 3, 2, 4)]
Perm.S4.Bin$PV.dist.Pval
# Adjust p-value.
round(matrix(p.adjust(Perm.S4.Bin$PV.dist.Pval, method = "fdr"),
    nrow = 4, dimnames = list(age.attr.b, age.attr.b)), 3)
```

The phylogenetic affinity of synechodontiforms as either the sister taxon to elasmobranchs or as part of Galeomorphii remains unresolved (Maisey 1985; Klug et al. 2009; Klug 2010; Maisey 2012). As a result, we performed a sensitivity analysis, whereby synechodontiforms were excluded from galeomorph-specific disparity trajectories to test the effect on both empirical morphospace occupation and disparity-levels.

```r
# 3a. Exclusion of Synechodontiformes from the global-level
# Selachimorpha analysis.  Four-stage time binning scheme.
# Sample size = 1182
Synechodontiformes <- which(Four.Df$Order == "Synechodontiformes")
subData <- Four.Df[-Synechodontiformes, ]
subData$Order <- factor(subData$Order)
# Fit disparity model.
Disp.C <- error.plot(gpa.coords = GPA$coords[, , rownames(subData)],
    blank = FALSE, groups = subData$Age.3, order = c(1, 3, 2,
        4), replicates = 999, rarefy.par = list(min.N = 187,
        reps = 999))
# 3b. Permutation test.
Perm.subS4.bin <- disparity.calc(gpa = GPA, data = subData, ages = "Age.3")
Perm.subS4.bin$Procrustes.var
Perm.subS4.bin$PV.dist.Pval
```

Global rarefaction graphic

```r
# lapply(names(Disp.A$rarefaction.results),function(x)
# write.xlsx(Disp.A$rarefaction.results[[x]],'output.xlsx',
# sheetName=x, append=TRUE))
rare.plot <- read.xlsx(file = "Morphometric & Other Excel Datasets/Global Shark Rarefaction.xlsx",
    sheetIndex = 1)
rare.plot <- rare.plot[rare.plot$Age %in% c("Campanian", "Maastrichtian",
    "Danian", "Thanetian"), ]
rare.plot$Age <- factor(rare.plot$Age)
rare.plot$Age <- factor(rare.plot$Age, levels = c("Campanian",
    "Maastrichtian", "Danian", "Thanetian"))

# Plot.
ggplot(rare.plot, mapping = aes(x = N2, y = rare.disp, colour = Age,
    group = Age)) + geom_errorbar(aes(ymin = lower.PI, ymax = upper.PI)) +
```

```
    geom_point(shape = 21, fill = "white") + geom_vline(xintercept = 28,
    linetype = "dashed", color = "black", size = 0.5) + scale_fill_manual(values = c("#E16A86",
    "#909800", "#00AD9A", "#9183E6")) + scale_color_manual(values = c("#E16A86",
    "#909800", "#00AD9A", "#9183E6")) + labs(title = "Selachimorpha",
    subtitle = "Sample rarefaction curves") + xlab("Sample Size") +
    ylab("Procrustes Variance") + theme(strip.text.x = element_text(size = 7,
    face = "bold"), axis.title = element_text(color = "#666666",
    face = "bold", size = 7), axis.text.y = element_text(angle = 90,
    size = 7, ), axis.text.x = element_text(size = 7), title = element_text(size = 7)) +
    theme(axis.text.x = element_text(angle = 45, vjust = 1, hjust = 1),
        panel.spacing = unit(0, "lines"), aspect.ratio = 2.5/3)
```

Partial Foote's Disparity

Calculating partial disparities served to investigate the effects of differential sampling between selachimorph clades in a given time-bin and determine their relative contribution to the overall pattern. For graphic results see the next section.

```
# 1. Split data frame by Age.
split.ls.a <- split(Five.Df, factor(Five.Df$Age))
split.ls.b <- split(Four.Df, factor(Four.Df$Age.3))

# 2. Partial disparity.
partial.disps <- vector(mode = "list", length = length(split.ls.b))
for (i in 1:length(split.ls.b)) {
    levs <- droplevels(split.ls.b[[i]][, 13])  # Orders.
    gdf.split <- geomorph.data.frame(coords = GPA$coords[, ,
        rownames(split.ls.b[[i]])], grp = levs)
    if (length(levels(levs)) < 2)
        disp <- morphol.disparity(coords ~ 1, data = gdf.split) else disp <- morphol.disparity(coords ~
        data = gdf.split, partial = TRUE)
    partial.disps[[i]] <- disp
}

names(partial.disps) <- c("Campanian", "Maastrichtian", "Danian",
    "Selandian", "Thanetian")
```

Family-level disparity assessment

Main rule: Families with sample <5 from any time bin is omitted prior to analysis. Specimens assigned as "Family incertae sedis" and "Incertae familiae" were also removed from this analysis. Resulting data: a total of 26 families and 1039 specimens when using the global four-stage time binning scheme.

```
omit.fam <- which(Four.Df$Family == "Family incertae sedis" |
    Four.Df$Family == "Incertae familiae")
Family.Frame <- Four.Df[-omit.fam, ]
Family.Frame <- Family.Frame[complete.cases(Family.Frame[, 14]),
    ]
# Create a new variable that represent a match of families
# and ages.
"%%" <- function(x, y) {
    paste(x, y, sep = "")
}
Family.Frame$famAge <- NULL
Family.Frame$famAge <- as.factor(Family.Frame$Family%%Family.Frame$Age.3)
```

```r
Trim.Fam <- Family.Frame %>%
    rownames_to_column("X") %>%
    group_by(famAge) %>%
    filter(n() >= 5) %>%
    column_to_rownames("X")
# Split by age.
Family.Split <- split(Trim.Fam, factor(Trim.Fam$Age.3))
# Partial disparity.
fam.disps <- vector(mode = "list", length = length(Family.Split))
for (i in 1:length(Family.Split)) {
    f.levs <- droplevels(Family.Split[[i]][, 14])  # Families
    f.split <- geomorph.data.frame(coords = GPA$coords[, , rownames(Family.Split[[i]])],
        grp = f.levs)
    if (length(levels(f.levs)) < 5)
        MD <- morphol.disparity(coords ~ 1, data = f.split) else MD <- morphol.disparity(coords ~ 1, gr
        partial = TRUE)
    fam.disps[[i]] <- MD
}
```

Plot partial disparity results Grouped bar plot for partial disparity

```r
PD_matrix <- read.xlsx(file = as.matrix("Morphometric & Other Excel Datasets/Partial Disparity.xlsx"),
    header = T, sheetIndex = 1)
rownames(PD_matrix) <- PD_matrix[, 1]
PD_matrix[, 1] <- NULL
PD_matrix <- as.matrix(PD_matrix)
# Bar plot.
layout(matrix(c(1, 1), nrow = 2), respect = TRUE)
bar.x <- barplot(PD_matrix, col = col.box, border = col.box,
    space = 0.15, font.axis = 2, xlab = "", ylab = "Procrustes Variance",
    axes = FALSE, axisnames = FALSE, axis.lty = 3, font.lab = 2,
    main = "Partial Disparity")
text(bar, par("usr")[3], labels = c("Campanian", "Maastrichtian",
    "Danian", "Selandian", "Thanetian", "Danian-Selandian"),
    font = 1, srt = 45, adj = c(1.1, 1.1), xpd = TRUE, cex = 0.9)
axis(2, las = 3, tick = 0.25, font = 1, lty = 1, lwd = 1)
abline(v = 5.85, col = "black", lwd = 1.5, lty = 2)
```

```r
dev.off()
```

```
## null device
##           1
```

```r
# Plot using ggplot.
pd.orders <- read.xlsx(file = "Morphometric & Other Excel Datasets/Partial Disparity.xlsx",
    sheetIndex = 1)
colnames(pd.orders)[1] <- "Orders"
colnames(pd.orders)[7] <- "Danian-Selandian"
# Melt.
pd.melt <- melt(pd.orders)
```

```
## Using Orders as id variables
```

```r
pd.melt$Orders <- factor(pd.melt$Orders, levels = c("Lamniformes",
    "Carcharhiniformes", "Heterodontiformes", "Orectolobiformes",
```

```
    "Hexanchiformes", "Squaliformes", "Echinorhiniformes", "Squatiniformes",
    "Synechodontiformes"))
# Plot.
ggplot(data = pd.melt, mapping = aes(x = variable, y = value,
    color = Orders, fill = Orders)) + geom_bar(stat = "identity",
    color = "black", lwd = 0.2) + coord_flip() + ylab("Procrustes Variance") +
    xlab("") + labs(title = "Ordel-level partial disparity") +
    scale_fill_manual(values = col.box) + geom_vline(xintercept = 5.5,
    linetype = "solid") + geom_vline(xintercept = 2.5, linetype = "dashed") +
    theme(axis.title = element_text(color = "#666666", face = "bold",
        size = 8), axis.text.y = element_text(angle = 0, size = 8),
        axis.text.x = element_text(size = 8), legend.key.size = unit(0.5,
            "lines"), legend.text = element_text(size = 8), legend.title = element_text(size = 10),
        legend.position = "right", panel.spacing = unit(0, "lines"),
        aspect.ratio = 2/3)
```

```
# Import results.
family.disp <- read.xlsx(file = "Morphometric & Other Excel Datasets/Family Disparity.xlsx",
    sheetIndex = 1)
family.disp.melt <- melt(family.disp, id.vars = c("Family", "Orders"))
# Plot.
family.disp.melt$variable <- factor(x = family.disp.melt$variable,
    levels = c("Campanian", "Maastrichtian", "Danian.Selandian",
        "Thanetian"))
family.disp.melt$Orders <- factor(x = family.disp.melt$Orders,
    levels = box.labels)
# Plot.
ggplot(data = family.disp.melt, mapping = aes(x = variable, y = value,
    color = Family, fill = Family)) + geom_bar(stat = "identity",
    color = "black", lwd = 0.2) + coord_flip() + xlab("Age") +
    scale_y_continuous(labels = scales::number_format(accuracy = 0.001,
        decimal.mark = ".")) + scale_fill_viridis(discrete = TRUE,
    alpha = 0.6) + scale_color_viridis(discrete = TRUE, alpha = 0.6) +
    theme(axis.title = element_text(color = "#666666", face = "bold",
        size = 7), axis.text.y = element_text(angle = 0, size = 7),
        axis.text.x = element_text(size = 7), legend.key.size = unit(0.5,
            "lines"), legend.text = element_text(size = 7), legend.position = "right",
        panel.spacing = unit(0, "lines"), aspect.ratio = 2/3) +
    guides(colour = guide_legend(nrow = 1))
```

Plot family disparity

First-order sensitivity analysis

Disparity is here calculated while accounting for heterodonty (i.e., variation in teeth along and between jaws) as a nominal factor. The natural grouping of teeth (i.e., positional categories) between major orders of sharks varies substantially e.g., in Lamniformes anterior & lateroposterior positions can be differentiated [following the row-group terminology of Siverson (1999)]. Differences in applicable terminology further complicates matters **(e.g., Shimada (1997a) uses anterior, intermediates and lateral; and Cappetta (2012) uses anterior, lateral and posterior**. This present a major challenge for how to assess the overall and immediate effect of heterodonty on our evolutionary time-series. Yet, despite these issues, we here attempt to estimate the disparity of all sharks while controlling for different types of heterodonty. Three models are constructed: 1) a monognathic 2) dignathic and 3) an interaction model (i.e., position and

unit) using the global four-stage time binning scheme. Ultimately, we rely on pre-assigned tooth positions for this part of the analysis. Here, monognathic heterodonty (MH) was defined as **parasymphyseal, anterior, lateroposterior, and posterior** tooth positions (NMH=897), whereas dignathic heterodonty (DH) was segregated by the **upper and lower** tooth rows (NDH=334). We recognize however that this terminology is not fully applicable to all sharks (e.g., hexanchids, have three definable categories of teeth in the lower jaw; a single symphyseal tooth, anterolateral teeth (five or six teeth with a very similar morphology) and tiny, molariform teeth near the commissure). Parasymphyseal teeth are present in the upper jaw of hexanchids but not in the lower jaw. The separation of anterior and anterolateral teeth in squalid shark is also not straightforward. As an additional check we estimated disparity using all unambiguous and pre-designed positional categories as a validation test. However, we recognize that difference in nomenclature have not been corrected for. This second trial involved the removal of ambiguous designations (e.g., anterior/parasymphyseal) and correcting spelling differences (e.g., symphyseal vs. symphysial). I have assumed that latero-anterior and anterolateral are the same type of teeth but different from lateroposterior (also equals "posterolateral"). The resulting categories is as follows: **anterior,anterolateral,commissural,intermediate,lateral,lateroposterior,parasymphyseal,posterior,symphyseal**. **'Commissural'** refers to the teeth at the corners of the mouth. **Results:** Using both approaches revealed a significant increase in tooth disparity across the K/Pg boundary in Selachimorpha when accounting for the effect of monognathic heterodonty, but not by a dignathic and combined heterodonty model.

```r
### First strategy.

# 1. Tooth positions.
positions <- c("anterior", "lateroposterior", "parasymphyseal",
    "posterior")
# 2. Data frame: 897 specimens.
monoGnathic.Df <- Four.Df[Four.Df$Standardized.Position %in%
    positions, ]
# 3. Dental units: 334 specimens.
dentalUnit <- c("upper", "lower")
dentalUnit.Df <- monoGnathic.Df[monoGnathic.Df$Dental.Unit %in%
    dentalUnit, ]

# 4. Monognathic data frame.
cov.disp_A <- geomorph.data.frame(gpa.coords = GPA$coords[, ,
    rownames(monoGnathic.Df)], position = factor(monoGnathic.Df$Standardized.Position),
    time = factor(monoGnathic.Df$Age.3), order = factor(monoGnathic.Df$Order))
# 5. Dignathic data frame.
cov.disp_B <- geomorph.data.frame(gpa.coords = GPA$coords[, ,
    rownames(dentalUnit.Df)], unit = factor(dentalUnit.Df$Dental.Unit),
    position = factor(dentalUnit.Df$Standardized.Position), time = factor(dentalUnit.Df$Age.3),
    order = factor(dentalUnit.Df$Order))

# 6. Monognathic Model 1: tooth position as a factor.
m.pos <- morphol.disparity(gpa.coords ~ position + time, groups = ~time,
    iter = 999, data = cov.disp_A)
# 7. P-value adjustment.
round(matrix(p.adjust(m.pos$PV.dist.Pval, method = "fdr"), nrow = 4,
    dimnames = list(age.attr.b, age.attr.b)), digits = 3)
# 8. Dignathic Model 2: dental unit as a factor.
d.pos <- morphol.disparity(gpa.coords ~ unit + time, groups = ~time,
    iter = 999, data = cov.disp_B)
# 9. P-value adjustment.
round(matrix(p.adjust(d.pos$PV.dist.Pval, method = "fdr"), nrow = 4,
    dimnames = list(age.attr.b, age.attr.b)), digits = 3)
```

```r
# 10. Model 3: dental unit + position as factors.
md.pos <- morphol.disparity(gpa.coords ~ position + unit + time,
    groups = ~time, iter = 999, data = cov.disp_B)
# 11. P-value adjustment.
round(matrix(p.adjust(md.pos$PV.dist.Pval, method = "fdr"), nrow = 4,
    dimnames = list(age.attr.b, age.attr.b)), digits = 3)

# 12. Model 4: position + order as factors.
complex.mod <- morphol.disparity(gpa.coords ~ time + position +
    order, groups = ~time, iter = 999, data = cov.disp_A)
round(matrix(p.adjust(complex.mod$PV.dist.Pval, method = "fdr"),
    nrow = 4, dimnames = list(age.attr.b, age.attr.b)), digits = 3)
```

```r
# Sample standardized
x.model <- lm.rrpp(f1 = two.d.array(gpa.coords) ~ time + position,
    iter = 999, RRPP = T, data = cov.disp_B)
y.model <- lm.rrpp(f1 = two.d.array(gpa.coords) ~ time + unit,
    iter = 999, RRPP = T, data = cov.disp_B)
z.model <- lm.rrpp(f1 = two.d.array(gpa.coords) ~ time + position +
    unit, iter = 999, RRPP = T, data = cov.disp_B)
r.model <- lm.rrpp(f1 = two.d.array(gpa.coords) ~ time + position +
    unit + order, iter = 999, RRPP = T, data = cov.disp_B)
u.model <- lm.rrpp(f1 = two.d.array(gpa.coords) ~ time, iter = 999,
    RRPP = T, data = cov.disp_B)
q.model <- lm.rrpp(f1 = two.d.array(gpa.coords) ~ 1, iter = 999,
    RRPP = T, data = cov.disp_B)  # Null model
# Best fit.
modComp <- model.comparison(x.model, y.model, z.model, r.model,
    u.model, q.model, type = "logLik")
summary(modComp)
anova(u.model, x.model, y.model, z.model, r.model)

# Increase sample.
x2.model <- lm.rrpp(f1 = two.d.array(gpa.coords) ~ time + position,
    iter = 999, RRPP = T, data = cov.disp_A)
y2.model <- lm.rrpp(f1 = two.d.array(gpa.coords) ~ time + position +
    order, iter = 999, RRPP = T, data = cov.disp_A)
z2.model <- lm.rrpp(f1 = two.d.array(gpa.coords) ~ time, iter = 999,
    RRPP = T, data = cov.disp_A)
r2.model <- lm.rrpp(f1 = two.d.array(gpa.coords) ~ 1, iter = 999,
    RRPP = T, data = cov.disp_A)  # Null model
# Best fit.
modComp.2 <- model.comparison(x2.model, y2.model, z2.model, r2.model,
    type = "logLik")
summary(modComp.2)
anova(z2.model, x2.model, y2.model)
anova(x2.model, y2.model)  # The most complex model appear to describe things better
```

Second strategy

```r
# New column for tooth positions.
Four.Df$St.Pos.X <- NULL
Four.Df$St.Pos.X <- Four.Df$Relative.Position

# Positions.
```

```r
alt.pos <- c("anterior", "anterolateral", "commissural", "intermediate",
    "lateral", "lateroposterior", "parasymphyseal", "posterior",
    "symphyseal")
# Data frame: 899 specimens.
second.trial <- Four.Df[Four.Df$St.Pos.X %in% alt.pos, ]
# Monognathic data frame.
sec.disp <- geomorph.data.frame(gpa.coords = GPA$coords[, , rownames(second.trial)],
    position = factor(second.trial$St.Pos.X), time = factor(second.trial$Age.3))
# Disparity model: P-value does not need to be sampled
# correct.
m.disp <- morphol.disparity(gpa.coords ~ time + position, groups = ~time,
    iter = 999, data = sec.disp)
```

Plot comparative disparity results

```r
disp.hetero <- read.xlsx(file = "Morphometric & Other Excel Datasets/Disparity vs. Heterodonty.xlsx",
    sheetIndex = 1)
disp.hetero$Age <- factor(disp.hetero$Age, levels = c("Campanian",
    "Maastrichtian", "Danian/Selandian", "Thanetian"))
disp.hetero$Model <- factor(disp.hetero$Model, levels = c("Full PV",
    "Monognathic model", "Dignathic model", "Combined model",
    "Validation model", "Position + Order"))
# Stacked bar plot with multiple groups
ggplot(data = disp.hetero, aes(x = Age, y = Procrustes.Variance,
    fill = Model)) + geom_bar(position = "dodge", stat = "identity") +
    ylab("Procrustes Variance") + scale_fill_manual(values = c("#1B9E77",
    "#D95F02", "#7570B3", "#E7298A", "#66A61E", "#E6AB02")) +
    scale_color_manual(values = c("#1B9E77", "#D95F02", "#7570B3",
        "#E7298A", "#66A61E", "#E6AB02")) + theme_grey() + labs(title = "Dental disparity in Selachimorp
    theme(axis.title = element_text(size = 7), strip.text.x = element_text(size = 7,
        face = "bold"), axis.text.y = element_text(angle = 90,
        size = 7), axis.text.x = element_text(size = 7, angle = 45,
        vjust = 0.7), aspect.ratio = 2/3)
```

Second-order sensitivity analysis

The early Paleocene sample is biased toward occurrences from the Danian section at the World heritage site Stevns Klint in Denmark. Here, we test for a lagerstatten effect on our morphospace and disparity for Selachimorpha traversing the K/Pg boundary. The following analysis is performed using the global four-stage time binning scheme (N=1045). Disparity values are not subjected to FDR-adjusted for small sample size. **Disparity Results:** No significant change across the K/Pg boundary when excluding Danian specimens from Denmark. **Morphospace results:** Pairwise statistics support a significant shift in average morphospace across the K/Pg boundary across all pc-axes and PC1 to PC3, but not PC4.

```r
# First delete all occurrences from Denmark.
ex.Denmark <- which(Data$Country == "Denmark")
ex.Denmark <- Data[-ex.Denmark, ]
# Create new data frame.
ex.Denmark <- ex.Denmark[ex.Denmark$Age.3 %in% geo.ages, ]
# Geomorph data frame.
df.lagerst <- geomorph.data.frame(gpa.coords = GPA$coords[, ,
    rownames(ex.Denmark)], time = factor(ex.Denmark$Age.3))
# Disparity model.
M.disp <- morphol.disparity(gpa.coords ~ time, groups = ~time,
```

```
    iter = 999, data = df.lagerst)
# Morphospace data frame.
df.lagerst.morpho <- geomorph.data.frame(pcs = scores[rownames(ex.Denmark),
    ], pc1 = scores[rownames(ex.Denmark), 1], pc2 = scores[rownames(ex.Denmark),
    2], pc3 = scores[rownames(ex.Denmark), 3], pc4 = scores[rownames(ex.Denmark),
    4], time = factor(ex.Denmark$Age.3))
# Fit anova model.
Stevsklint.aov <- lm.rrpp(f1 = pcs ~ time, iter = 999, RRPP = TRUE,
    data = df.lagerst.morpho)
anova(Stevsklint.aov)
# Pairwise statistics
PW.SK <- pairwise(fit = Stevsklint.aov, groups = interaction(ex.Denmark$Age.3))
SM.PW <- summary(PW.SK, test.type = "dist", confidence = 0.95,
    stat.table = TRUE)
# FDR-adjusted p-values.
round(matrix(p.adjust(SM.PW$pairwise.tables$P, method = "fdr"),
    nrow = 4, dimnames = list(age.attr.b, age.attr.b)), digits = 3)
```

Super-order disparity analysis

In the first iteration Synechodontiformes is considered part of the main analysis for Galeomorphii. Disparity for the Galeomorphii is also evaluated by excluding Synechodontiformes as a sensitivity test. Procrustes variance is here only performed using the four-stage time binning scheme.

```
# 1a. Galeomorphii.  Sample size = 1043.  Sub-sampling
# level used here is the Thanetian.
Galeo.4.Bin <- error.plot(gpa.coords = GPA$coords[, , rownames(Galeo.Df)],
    blank = FALSE, groups = Galeo.Df$Age.3, order = c(1, 3, 2,
        4), replicates = 999, rarefy.par = list(min.N = 169,
        reps = 999))
# 1b. Permutations re-sampling procedure test.
Galeo.PermutationTest <- disparity.calc(gpa = GPA, data = Galeo.Df,
    ages = "Age.3")
Galeo.PermutationTest$Procrustes.var
Galeo.PermutationTest$PV.dist.Pval
# Adjust p-value.
matrix(p.adjust(Galeo.PermutationTest$PV.dist.Pval, method = "fdr"),
    4, 4)
```

- **Excluding Synechodontiformes**.

```
# 2a. Galeomorphii (sensitivity analysis) Sample size =
# 1027
subGaleo.Syn <- Galeo.Df[Galeo.Df$Order != "Synechodontiformes",
    ]
subGaleo.Syn$Order <- factor(subGaleo.Syn$Order)

subGaleo.4.Bin <- error.plot(gpa.coords = GPA$coords[, , rownames(subGaleo.Syn)],
    blank = FALSE, groups = subGaleo.Syn$Age.3, order = c(1,
        3, 2, 4), replicates = 999, rarefy.par = list(min.N = 169,
        reps = 999))

# 2b. Permutations re-sampling procedure test.
Exclu.Galeo.PermutationTest <- disparity.calc(gpa = GPA, data = subGaleo.Syn,
    ages = "Age.3")
```

```r
Exclu.Galeo.PermutationTest$Procrustes.var
Exclu.Galeo.PermutationTest$PV.dist.Pval
# 2c. Adjust p-value.
matrix(p.adjust(Exclu.Galeo.PermutationTest$PV.dist.Pval, method = "fdr"),
    ncol = 4, nrow = 4)
```

```r
# 3a. Squalimorphii.
Squali.Df$Superorder <- factor(Squali.Df$Superorder)
Squali.4.Bin <- error.plot(gpa.coords = GPA$coords[, , rownames(Squali.Df)],
    blank = FALSE, groups = Squali.Df$Age.3, order = c(1, 3,
        2, 4), replicates = 999, rarefy.par = list(min.N = 18,
        reps = 999))
# 3b. Permutations re-sampling procedure test.
Squali.PermutationTest <- disparity.calc(gpa = GPA, data = Squali.Df,
    ages = "Age.3")
Squali.PermutationTest$Procrustes.var
Squali.PermutationTest$PV.dist.Pval
# 3c.Adjust p-value.
matrix(p.adjust(Squali.PermutationTest$PV.dist.Pval, method = "fdr"),
    ncol = 4, nrow = 4)
```

Global sub-stage disparity analysis

Our global occurrence dataset include specimens that have been assigned to specific geological sub-divisions (e.g., early, middle, and late). This presents an opportunity to explore patterns of morphological richness at a more temporally resolved scale. Here, we use a *two-fold division for the Campanian* and the Maastrichtian and a *three-fold for the Danian*. This binning scheme slightly deviates from established chronostratigraphic divisions. This applies mainly to the Campanian that includes a two-fold division (upper and lower) except for in North America. Importantly, however, our binning scheme here is solely predicated on dated assignments and does not seek to create new ages. Sampling level below 10 is insufficient for bootstrap calculations to be completed: hence why the middle Maastrichtian (N=6) has been omitted from this analysis. For the rarefaction analysis the lowest sampled age is the late Danian (N=11).

```r
# 1a. Global Selachimorpha: 659 specimens.
Data$SubAges <- paste(Data$Sub, Data$Age)
Data$SubAges <- factor(Data$SubAges)
# Select levels.
select.levels <- c("early Campanian", "late Campanian", "early Maastrichtian",
    "late Maastrichtian", "early Danian", "middle Danian", "late Danian")

subAge.Data <- Data[Data$SubAges %in% select.levels, ]
subAge.Data$SubAges <- factor(subAge.Data$SubAges)

# Remove North American occurrence from the late Campanian.
# The late Campanian (as used in North America) equals
# roughly the later half of the late Campanian as used
# elsewhere.
rm.lCmpAmer <- which(subAge.Data$SubAges == "late Campanian" &
    subAge.Data$Continent == "North America")
# Subset again.
subAge.Data <- subAge.Data[-rm.lCmpAmer, ]
subAge.Data$SubAges <- factor(subAge.Data$SubAges)
subAge.Data$Continent <- factor(subAge.Data$Continent)
# 1b. DTT over sub-age level time bins.
```

```
subStage.DTT <- error.plot(gpa.coords = GPA$coords[, , rownames(subAge.Data)],
    blank = FALSE, groups = subAge.Data$SubAges, order = c(1,
        7, 4, 3, 6, 2, 8, 5), replicates = 999, rarefy.par = list(min.N = 11,
        reps = 999))

# 1c. Permutation test.
substage.Selachi.Permutation <- disparity.calc(gpa = GPA, data = subAge.Data,
    ages = "SubAges")
substage.Selachi.Permutation$Procrustes.var[c(1, 7, 4, 3, 9,
    6, 2, 8, 5)]
substage.Selachi.Permutation$PV.dist.Pval
```

```
# 2a. Global substage-level analysis of Galeomorphii
# including Synechodontiformes.  N = 568 specimens.
sub.Galeo.Syn <- subset(subAge.Data, subset = subAge.Data$Superorder ==
    "Galeomorphii")
sub.Galeo.Syn$Superorder <- factor(sub.Galeo.Syn$Superorder)
# Remove the middle Maastrichtian and late Danian (N=5).
sub.Galeo.Syn <- sub.Galeo.Syn[sub.Galeo.Syn$SubAges != "middle Maastrichtian" &
    sub.Galeo.Syn$SubAges != "late Danian", ]
# 2b. DTT over substage-level time bins.
Galeo.Syn.subStage.DTT <- error.plot(gpa.coords = GPA$coords[,
    , rownames(sub.Galeo.Syn)], blank = FALSE, groups = sub.Galeo.Syn$SubAges,
    order = c(1, 4, 3, 5, 2, 6), replicates = 999, rarefy.par = list(min.N = 17,
        reps = 999))
```

```
# 3a. Global substage-level analysis of Galeomorphii
# excluding Synechodontiformes.  N = 554 specimens.
sub.Galeo <- sub.Galeo.Syn[sub.Galeo.Syn$Order != "Synechodontiformes",
    ]
sub.Galeo$Order <- factor(sub.Galeo$Order)
# 3b. DTT over substage-level time bins.  Sub-sampling is
# performed on the the middle Campanian sample size (N=38).
Galeo.subStage.DTT <- error.plot(gpa.coords = GPA$coords[, ,
    rownames(sub.Galeo)], blank = FALSE, groups = sub.Galeo$SubAges,
    order = c(1, 4, 3, 5, 2, 6), replicates = 999, rarefy.par = list(min.N = 38,
        reps = 999))
# 3c. Permutation test.
substage.Galeo.Permutation <- disparity.calc(gpa = GPA, data = sub.Galeo,
    ages = "SubAges")
```

```
# 4a. Global substage-level analysis of Squalomorphii.
Squali.Df$SubAges <- paste(Squali.Df$Sub, Squali.Df$Age)
# Select levels.
sq.levels <- c("early Campanian", "early Maastrichtian", "late Maastrichtian",
    "early Danian")
sub.Squali.Df <- Squali.Df[Squali.Df$SubAges %in% sq.levels,
    ]
# N=70
sub.Squali.Df$SubAges <- factor(sub.Squali.Df$SubAges)
# 4b. DTT over stage-level time bins.
Squali.subStage.DTT <- error.plot(gpa.coords = GPA$coords[, ,
    rownames(sub.Squali.Df)], blank = FALSE, groups = sub.Squali.Df$SubAges,
    order = c(1, 3, 4, 2), replicates = 999, rarefy.par = list(min.N = 12,
```

```
        reps = 999))
# 4c. Permutation test.
disparity.calc(gpa = GPA, data = sub.Squali.Df, ages = "SubAges")
```

Plot sub-age disparity results

```
# Graphics.
sub.Disp <- read.xlsx(file = "Morphometric & Other Excel Datasets/Substage Selachimorpha Disparity.xlsx
    sheetIndex = 1)
pd.x <- position_dodge(2.5)
# Clades.
sub.Disp$Group <- factor(sub.Disp$Group, levels = c("Selachimorpha",
    "Galeomorphii", "Squalimorphii"))
# Sub-stages.
sub.Disp$Geological.Substages <- factor(sub.Disp$Geological.Substages,
    levels = c("early Campanian", "late Campanian", "early Maastrichtian",
        "late Maastrichtian", "early Danian", "middle Danian",
        "late Danian"))

# Plot disparity results.
K1 <- ggplot(sub.Disp, aes(x = Geological.Substages, y = Disparity,
    colour = Group, group = Group)) + geom_errorbar(mapping = aes(ymin = LowerBoot.PI,
    ymax = UpperBoot.PI), width = 0.2, size = 1) + geom_line() +
    geom_point(mapping = aes(x = Geological.Substages, y = Disparity)) +
    geom_point(size = 2, shape = 21, fill = "white") + xlab("Sub-stage") +
    ylab("Procrustes Variance") + geom_vline(aes(xintercept = 6.5),
    linetype = "dashed", colour = "black", size = 1.5) + theme_bw() +
    theme(axis.title.x = element_text(face = "bold"), axis.title.y = element_text(face = "bold")) +
    scale_colour_manual(values = c("#233B43", "#A76281", "#61A375")) +
    theme(legend.position = "none", axis.title = element_text(size = 8),
        strip.text.x = element_text(size = 8, face = "bold"),
        axis.text.y = element_text(angle = 90, size = 8), axis.text.x = element_text(size = 8,
            angle = 45, vjust = 1, hjust = 1), aspect.ratio = 2/3) +
    facet_wrap(~Group, nrow = 3, scales = "free_y")

# Bar plot
K2 <- ggplot(sub.Disp, aes(x = Geological.Substages, y = Sample.Size,
    colour = Group, group = Group)) + geom_bar(stat = "identity") +
    xlab("Sub-stages") + ylab("Sample Size") + scale_colour_manual(values = c("#233B43",
    "#A76281", "#61A375")) + scale_fill_manual(values = c("#233B43",
    "#A76281", "#61A375")) + theme(legend.position = "none",
    axis.title = element_text(size = 8), strip.text.x = element_text(size = 8,
        face = "bold"), axis.text.y = element_text(angle = 90,
        size = 8), axis.text.x = element_text(size = 8, angle = 45,
        vjust = 1, hjust = 1), aspect.ratio = 2/3) + facet_wrap(~Group,
    ncol = 1, scales = "free_y")

ggarrange(K1, K2)
```

```
subage.barplot <- read.xlsx(file = "Morphometric & Other Excel Datasets/Substage Selachimorpha Disparit
    sheetIndex = 2)
# Clades.
subage.barplot$Group <- factor(subage.barplot$Group, levels = c("Selachimorpha",
```

```
    "Galeomorphii", "Squalimorphii"))
# Sub-stages.
subage.barplot$Geological.Substages <- factor(subage.barplot$Geological.Substages,
    levels = c("early Campanian", "late Campanian", "early Maastrichtian",
        "late Maastrichtian", "early Danian", "middle Danian",
        "late Danian"))
# Disparity as bar-plots
ggplot(subage.barplot, aes(x = Geological.Substages, y = Disparity,
    colour = Group, group = Group)) + geom_bar(stat = "identity") +
    xlab("Sub-stages") + ylab("Procrustes variance") + scale_colour_manual(values = c("#233B43",
    "#A76281", "#61A375")) + scale_fill_manual(values = c("#233B43",
    "#A76281", "#61A375")) + theme(legend.position = "none",
    axis.title = element_text(size = 8), strip.text.x = element_text(size = 8,
        face = "bold"), axis.text.y = element_text(angle = 90,
        size = 8), axis.text.x = element_text(size = 8, angle = 45,
        vjust = 1, hjust = 1), aspect.ratio = 2/3) + facet_wrap(~Group,
    ncol = 3, scales = "free_y")
```

Regional level disparity analysis

The following section explores variance within our regional sub-sample obtained from Stevns Klint in Denmark. Sample size for total-sharks: late Maastrichitan = 48, early Danian = 80, middle Danian = 25. **Number of replicates need to exceed the number of observations**.

```
# 1a. Regional Selachimorpha: 153 specimens.
reg.Selachi <- error.plot(gpa.coords = GPA$coords[, , rownames(Three.Df)],
    blank = FALSE, groups = Three.Df$Sub, order = c(2, 1, 3),
    replicates = 999, rarefy.par = list(min.N = 25, reps = 999))

disparity.calc(gpa = GPA, data = Three.Df, ages = "Sub")
```

```
# 2a. Regional Galeomorphii including Synechodontiformes.
# 116 specimens: late Maastrichtian = 32; early Danian =
# 61; middle Danian = 23.
reg.Galeo.Syn <- subset(Three.Df, subset = Three.Df$Superorder ==
    "Galeomorphii")
reg.Galeo.Syn$Superorder <- factor(reg.Galeo.Syn$Superorder)
# 2b. Disparity model.
reg.Galeo.Syn.Disp <- error.plot(gpa.coords = GPA$coords[, ,
    rownames(reg.Galeo.Syn)], blank = FALSE, groups = reg.Galeo.Syn$Sub,
    order = c(2, 1, 3), replicates = 999, rarefy.par = list(min.N = 23,
        reps = 999))

disparity.calc(gpa = GPA, data = reg.Galeo.Syn, ages = "Sub")
```

```
# 3a. Regional Galeomorphii excluding Synechodontiformes.
# 106 specimens.
reg.Galeo <- reg.Galeo.Syn[reg.Galeo.Syn$Order != "Synechodontiformes",
    ]
reg.Galeo$Order <- factor(reg.Galeo$Order)
# Disparity model.
reg.Galeo.disp <- error.plot(gpa.coords = GPA$coords[, , rownames(reg.Galeo)],
    blank = FALSE, groups = reg.Galeo$Sub, order = c(2, 1, 3),
    replicates = 999, rarefy.par = list(min.N = 17, reps = 999))
```

```
disparity.calc(gpa = GPA, data = reg.Galeo, ages = "Sub")
```

```
# 4a. Regional Squalimorphii: 153 specimens.  Available
# orders: Hexanchiformes, Squaliformes and Squatiniformes.
# The middle Danian is omitted from this analysis because
# of low sample size (N=2)
reg.Squali <- subset(Three.Df, subset = Three.Df$Superorder ==
    "Squalomorphii")
reg.Squali <- reg.Squali[reg.Squali$Sub != "middle", ]
reg.Squali$Sub <- factor(reg.Squali$Sub)
# Disparity model.
regional.Squali <- error.plot(gpa.coords = GPA$coords[, , rownames(reg.Squali)],
    blank = FALSE, groups = reg.Squali$Sub, order = c(2, 1),
    replicates = 999, rarefy.par = list(min.N = 16, reps = 999))

disparity.calc(gpa = GPA, data = reg.Squali, ages = "Sub")
```

Order-level disparity analysis

Galeomorph sub-clades The representation of synechodontiforms from the Campanian-Thanetian interval is shape biased. It is also clear that many Synechodontids have a clutching/crushing dentition (with emphasis on crushing) throughout their temporal range; a signal that we fail to detect as a result of inadequate sampling of relevant tooth positions (i.e., lateral and posterior teeth of this group). Further, 10 out of 16 synechodontiform specimens are from Denmark.

```
# 1a. Lamniformes.
Lamn.Df <- which(Data$Order == "Lamniformes")
Lamn.Df <- Data[Lamn.Df, ]
Lamn.Df <- Lamn.Df[Lamn.Df$Age.3 %in% geo.ages, ]
# Disparity model.
Lamn.4.Bin <- error.plot(gpa.coords = GPA$coords[, , rownames(Lamn.Df)],
    blank = FALSE, groups = Lamn.Df$Age.3, order = c(1, 3, 2,
        4), replicates = 999, rarefy.par = list(min.N = 82, reps = 999))
# Permutation test.
L.calc <- disparity.calc(gpa = GPA, data = Lamn.Df, ages = "Age.3")
matrix(p.adjust(p = L.calc$PV.dist.Pval, method = "fdr"), 4,
    4)


# 2a. Carcharhiniformes.
Carch.Df <- which(Data$Order == "Carcharhiniformes")
Carch.Df <- Data[Carch.Df, ]
Carch.Df <- Carch.Df[Carch.Df$Age.3 %in% geo.ages, ]
# Disparity model.
Carch.4.Bin <- error.plot(gpa.coords = GPA$coords[, , rownames(Carch.Df)],
    blank = FALSE, groups = Carch.Df$Age.3, order = c(1, 3, 2,
        4), replicates = 999, rarefy.par = list(min.N = 34, reps = 999))
# Permutation test.
C.calc <- disparity.calc(gpa = GPA, data = Carch.Df, ages = "Age.3")
matrix(p.adjust(p = C.calc$PV.dist.Pval, method = "fdr"), 4,
    4)

# 3a. Heterodontiformes.  Sampling across all time-bins is
# below 10. Procrustes variances is estimated without
```

```
# bootstrapping and rarefaction.  Ultimately, it makes no
# sense pseudo-replicating such a small sample size any
# way.
Hetero.Df <- which(Data$Order == "Heterodontiformes")
Hetero.Df <- Data[Hetero.Df, ]
Hetero.Df <- Hetero.Df[Hetero.Df$Age.3 %in% geo.ages, ]
# Geomorph data frame.
Hetero.Gdf <- geomorph.data.frame(coords = GPA$coords[, , rownames(Hetero.Df)],
    age = factor(Hetero.Df$Age.3))
# 4a. Orectolobiformes.
Orect.Df <- which(Data$Order == "Orectolobiformes")
Orect.Df <- Data[Orect.Df, ]
Orect.Df <- Orect.Df[Orect.Df$Age.3 %in% geo.ages, ]
# Disparity model.
Orect.4.Bin <- error.plot(gpa.coords = GPA$coords[, , rownames(Orect.Df)],
    blank = FALSE, groups = Orect.Df$Age.3, order = c(1, 3, 2,
        4), replicates = 999, rarefy.par = list(min.N = 15, reps = 999))
# Permutation test.
o.perm <- disparity.calc(gpa = GPA, data = Orect.Df, ages = "Age.3")
# Adjust p-values.  K/Pg non-significant (adjusted
# p-value).
round(matrix(p.adjust(o.perm$PV.dist.Pval, method = "fdr"), nrow = 4,
    dimnames = list(age.attr.b, age.attr.b)), 3)

# 5a. Synechodontiformes: Sampling across all time-bins is
# very low.
Syn.Df <- which(Data$Order == "Synechodontiformes")
Syn.Df <- Data[Syn.Df, ]
Syn.Df <- dplyr::filter(Syn.Df, !is.na(Age.3))  # remove NA.
# Force rownames back.
rownames(Syn.Df) <- paste(Syn.Df$File.Name, Syn.Df$File.Type,
    sep = "")
# Geomorph data frame.
Syn.Gdf <- geomorph.data.frame(coords = GPA$coords[, , rownames(Syn.Df)],
    age = factor(Syn.Df$Age.3))
# Disparity model.
Syn.3.Bin <- morphol.disparity(f1 = coords ~ age, groups = ~age,
    iter = 999, data = Syn.Gdf)
```

Squalimorph sub-clades According to our data level entries the disparity of Hexanchiformes is estimated principally on the basis of anterolateral (N=18) and lateral (N=12) teeth. Three anterior and symphyseal teeth are also considered. The Danian-Selandian sample is dominated by anterolateral teeth whereas the Thanetian include more lateral tooth positions. It is possible that these two categories are synonymous but used different by shark workers. Our squaliform shark sample is dominated by anterolateral (15), lateral (N=26) and anterior (N=13) tooth positions. The seperation of lateral and anterolateral teeth in squaliforms is doubtful. Sampling for squatiniform sharks comprise mainly lateral teeth (N=7).

```
# 1. Hexanchiformes.  Three-bins only: Maastrichtian (N=5),
# DanSelCombined (N=21), and Thanetian (N=14).
Hex.Df <- which(Four.Df$Order == "Hexanchiformes")
Hex.Df <- Four.Df[Hex.Df, ]
# A total of 40 specimens.
Hex.Df.Bot <- Hex.Df[Hex.Df$Age.3 != "Maastrichtian", ]
```

```r
# Bootstrap and rarefaction applied to the Danian-Selandian
# and Thantian samples only.
Hex.3.Bin <- error.plot(gpa.coords = GPA$coords[, , rownames(Hex.Df.Bot)],
    blank = FALSE, groups = Hex.Df.Bot$Age.3, order = c(1, 2),
    replicates = 999, rarefy.par = list(min.N = 14, reps = 999))
# Permutation test.
disparity.calc(gpa = GPA, data = Hex.Df.Bot, ages = "Age.3")
# MD for Hexanchiformes including the Maastrichtian.
Hex.Frame <- geomorph.data.frame(coords = GPA$coords[, , rownames(Hex.Df)],
    age = factor(Hex.Df$Age.3))
MD.Hex <- morphol.disparity(coords ~ age, groups = ~age, iter = 999,
    data = Hex.Frame)
matrix(p.adjust(MD.Hex$PV.dist.Pval, method = "fdr"), 3, 3)

# 2. Squaliformes.  Four-binning scheme, but like before
# start by omitting levels with sample size <10.  In this
# case the Thanetian (N=3).
Squal.Df <- which(Four.Df$Order == "Squaliformes")
Squal.Df <- Four.Df[Squal.Df, ]
# A total of 86 specimens.
Squal.Df.Bot <- Squal.Df[Squal.Df$Age.3 != "Thanetian", ]
# Disparity model.
Squal.4.Bin <- error.plot(gpa.coords = GPA$coords[, , rownames(Squal.Df.Bot)],
    blank = FALSE, groups = Squal.Df.Bot$Age.3, order = c(1,
        3, 2), replicates = 999, rarefy.par = list(min.N = 12,
        reps = 999))
# Permutation test.
s.perm <- disparity.calc(gpa = GPA, data = Squal.Df, ages = "Age.3")
p.adjust(s.perm$PV.dist.Pval, method = "fdr")
# MD for Squaliformes. including the Thanetian.
Squal.Frame <- geomorph.data.frame(coords = GPA$coords[, , rownames(Squal.Df)],
    age = factor(Squal.Df$Age.3))
MD.Squal <- morphol.disparity(coords ~ age, groups = ~age, iter = 999,
    data = Squal.Frame)
# Adjust P-values.
round(matrix(p.adjust(Squal.Permutation$PV.dist.Pval, method = "fdr"),
    nrow = 4, dimnames = list(age.names, age.names)), 3)

# Squaliforms likewise reduce their negative outliers along
# PC1 in the Maastrichtian time-bin; however, p-value
# correction (p=0.152) supported the null hypothesis of no
# overall temporal shift.

# 3. Squatiniformes.  Three-bins only: Campanian,
# Maastrichtian, and Danian-Selandian. Only one observation
# for the Thanetian.
Squat.Df <- which(Four.Df$Order == "Squatiniformes")
Squat.Df <- Four.Df[Squat.Df, ]
Squat.Df <- Squat.Df[Squat.Df$Age.3 != "Thanetian", ]
# Dispairty model
Squat.Frame <- geomorph.data.frame(coords = GPA$coords[, , rownames(Squat.Df)],
    age = factor(Squat.Df$Age.3))
Squat.3.Bin <- morphol.disparity(coords ~ age, groups = ~age,
```

```
    iter = 999, data = Squat.Frame)
```

Regional sub-clade level disparity analysis

Here we estimate the disparity of Lamniformes (N=27), Carcharhiniformes (N=50), Orectolobiformes (N=23), and Squaliformes (N=22) based on samples collected from Stevns Klint in Denmark.

```
# 1. Lamniformes.
Reg.Lamn.Df <- Three.Df[Three.Df$Order == "Lamniformes", ]
# 2. Disparity model.
Reg.Lamn.Disp <- error.plot(gpa.coords = GPA$coords[, , rownames(Reg.Lamn.Df)],
    blank = FALSE, groups = Reg.Lamn.Df$Sub, order = c(2, 1),
    replicates = 999, rarefy.par = list(min.N = 7, reps = 999))
# Permutation test.
disparity.calc(gpa = GPA, data = Reg.Lamn.Df, ages = "Sub")

# 3. Carcharhiniformes: zero sample for the middle Danian
Reg.Carch.Df <- Three.Df[Three.Df$Order == "Carcharhiniformes",
    ]
# 4. Disparity model.
Reg.Carch.Disp <- error.plot(gpa.coords = GPA$coords[, , rownames(Reg.Carch.Df)],
    blank = FALSE, groups = Reg.Carch.Df$Sub, order = c(2, 1),
    replicates = 999, rarefy.par = list(min.N = 14, reps = 999))
# Permutation test.
disparity.calc(gpa = GPA, data = Reg.Carch.Df, ages = "Sub")

# 5. Orectolobiformes.
Reg.Orec.Df <- Three.Df[Three.Df$Order == "Orectolobiformes",
    ]
# 6. Disparity model: Only the early Danian has a sample
# size (N=13).
Orect.Frame <- geomorph.data.frame(coords = GPA$coords[, , rownames(Reg.Orec.Df)],
    age = factor(Reg.Orec.Df$Sub))
Reg.Orec.Disp <- morphol.disparity(coords ~ age, groups = ~age,
    iter = 999, data = Orect.Frame)
# Permutation test.
disparity.calc(gpa = GPA, data = Reg.Orec.Df, ages = "Sub")

# 7. Squaliformes.
Reg.Squal.Df <- Three.Df[Three.Df$Order == "Squaliformes", ]
Reg.Squal.Disp <- error.plot(gpa.coords = GPA$coords[, , rownames(Reg.Squal.Df)],
    blank = FALSE, groups = Reg.Squal.Df$Sub, order = c(2, 1),
    replicates = 999, rarefy.par = list(min.N = 11, reps = 999))
# Permutation test.
disparity.calc(gpa = GPA, data = Reg.Squal.Df, ages = "Sub")
```

Plot clade disparity

```
Order.Disp <- read.xlsx(file = "Morphometric & Other Excel Datasets/Subclade Disparity Profiles.xlsx",
    sheetIndex = 1)
Order.Disp$Orders <- factor(Order.Disp$Orders, levels = c("Lamniformes",
    "Carcharhiniformes", "Heterodontiformes", "Orectolobiformes",
    "Hexanchiformes", "Squaliformes", "Squatiniformes", "Synechodontiformes"))
pd <- position_dodge(2.5)
```

```
R1 <- ggplot(Order.Disp, aes(x = Midpoint, y = Disparity, colour = Orders,
    group = Spatial.Scale, shape = Spatial.Scale)) + scale_x_reverse() +
    geom_errorbar(mapping = aes(ymin = LowerBoot.PI, ymax = UpperBoot.PI),
        width = 1, position = pd, show.legend = FALSE, size = 1) +
    geom_line() + geom_line(aes(linetype = "Spatial.Scale"),
    size = 0.5) + geom_point(aes(shape = Spatial.Scale, stroke = 1.4),
    fill = "white") + scale_shape_manual(values = c(21, 17)) +
    xlab("Time (Ma)") + ylab("Procrustes Variance") + geom_vline(xintercept = 66,
    color = "black", size = 2) + theme_bw() + theme(axis.title.x = element_text(face = "bold"),
    axis.title.y = element_text(face = "bold")) + scale_colour_manual(values = c("#E84646",
    "#377EB8", "darkorange", "purple4", "black", "green4", "violetred4",
    "darkcyan")) + theme(axis.title.x = element_text(face = "bold",
    size = 10), axis.title.y.left = element_text(face = "bold"),
    axis.text.y = element_text(size = 8, angle = 90), strip.text.x = element_text(size = 8,
        face = "bold"), axis.text = element_text(colour = "#4D4D4D",
        size = 8), legend.justification = c(0.05, 0.95), legend.position = "none",
    aspect.ratio = 1/3) + facet_wrap(~Orders, scales = "free_y",
    shrink = T, ncol = 2)

R1 + scale_y_continuous(labels = scales::number_format(accuracy = 0.01,
    decimal.mark = "."))
```

Oceanic basins N=1160 specimens. 1) African epicontinental basins, 2) Austral Tethys, 3) Eastern Pacific rim, 4) Weddellian Seaway, 5) Eastern Atlantic rim (West African shelf), 6) European epicontinental basins, 7) Mediterranean Tethys, 8) Western Atlantic rim (North American shelf), 9) Western Interior Seaway, 10) Western Pacific rim.

```
# Confirm whether there is a spatial shift in tooth
# disparity across the K/Pg boundary on a global scale and
# across basins.  Oceanic basins with sample size less than
# 10 are removed: xtabs(~Palaeo.Oceanic.Basin+Age.3, data =
# Four.Df)
Four.Df$BasinAge <- NULL
Four.Df$BasinAge <- as.factor(Four.Df$Palaeo.Oceanic.Basin%%Four.Df$Age.3)
Basins.Df <- Four.Df %>%
    rownames_to_column("X") %>%
    group_by(BasinAge) %>%
    filter(n() >= 10) %>%
    column_to_rownames("X")
# Split by age.
basin.split <- split(x = Basins.Df, f = factor(Basins.Df$Age.3))
# Compute the partial disparity sharks from oceanic basins
# across time.
basin.disps <- vector(mode = "list", length = length(basin.split))
for (i in 1:length(basin.split)) {
    b.levs <- droplevels(basin.split[[i]][, 17])
    b.split <- geomorph.data.frame(coords = GPA$coords[, , rownames(basin.split[[i]])],
        grp = b.levs)
    if (length(levels(b.levs)) < 2)
        disp <- morphol.disparity(coords ~ 1, data = b.split,
            print.progress = F) else disp <- morphol.disparity(coords ~ 1, groups = ~grp,
        data = b.split, partial = TRUE, print.progress = F)
    basin.disps[[i]] <- disp
}
```

```r
# Geomorph data frame: n = 1159.
acc.geo <- geomorph.data.frame(coords = GPA$coords[, , rownames(Basins.Df)],
    basins = factor(Basins.Df$Palaeo.Oceanic.Basin), age = factor(Basins.Df$Age.3))
# Selachimorpha disparity accounting for paleo-basins
acc.disp <- morphol.disparity(f1 = coords ~ age + basins, groups = ~age,
    iter = 999, data = acc.geo)
```

```r
BS.Res <- read.xlsx(file = "Morphometric & Other Excel Datasets/PaleoBasin Disparity.xlsx",
    sheetIndex = 1)
BS.Res$Age <- factor(BS.Res$Age, levels = c("Campanian", "Maastrichtian",
    "Danian/Selandian", "Thanetian"))
BS.Res %>%
    ggplot(aes(x = Age, y = Disparity, fill = Oceanic.Basin,
        color = Oceanic.Basin)) + geom_bar(position = position_dodge(preserve = "single"),
    stat = "identity", colour = "white") + theme_bw() + xlab("") +
    ylab("Procrustes Variance") + scale_fill_manual(values = c("slategray",
    "coral", "firebrick3", "orange", "#3b54bb", "springgreen4",
    "black")) + theme(axis.title = element_text(color = "#666666",
    face = "bold", size = 8), axis.text.y = element_text(angle = 90,
    size = 8), axis.text.x = element_text(angle = 45, vjust = 0.5,
    hjust = 0.75, size = 8), axis.line = element_line(linetype = "solid"),
    panel.spacing = unit(0, "lines"), aspect.ratio = 1)
```

Plot paleooceanic results

Sensitivity analyses

- Monognathic heterodonty in Selachimorpha. The positions used are the same as before ("anterior","lateroposterior","parasymphyseal","posterior").

```r
# Data frame with 897 selachimorph specimens.
m1 <- data.frame(scores = scores[rownames(monoGnathic.Df), 1:4],
    position = factor(monoGnathic.Df$Standardized.Position),
    order = factor(monoGnathic.Df$Order), unit = factor(monoGnathic.Df$Dental.Unit),
    time = factor(monoGnathic.Df$Age.3))

# This includes Echinorhiniformes.
new.orders <- c("Lamniformes", "Carcharhiniformes", "Heterodontiformes",
    "Orectolobiformes", "Hexanchiformes", "Squaliformes", "Echinorhiniformes",
    "Squatiniformes", "Synechodontiformes")
# Molten data frame.
me1_melt <- melt(m1)
```

```
## Using position, order, unit, time as id variables
```

```r
me1_melt$position <- factor(me1_melt$position, levels = c("parasymphyseal",
    "anterior", "lateroposterior", "posterior"))
# Monognathic morphospace for all sharks.  Box plots:
# PC1-PC4.
ggplot(me1_melt, aes(x = position, y = value, fill = position,
    colour = position)) + geom_point(position = position_jitterdodge(dodge.width = 0.9),
    alpha = 0.5, size = 0.2) + geom_boxplot(outlier.size = 3,
    alpha = 0.2, fill = "white") + stat_summary(fun.data = mean_cl_boot,
    geom = "errorbar", width = 0.1, lwd = 0.25, linetype = 1,
```

```
    col = "black") + stat_summary(fun = "mean", geom = "point",
    lwd = 2, position = position_dodge(width = 1), bg = "white",
    color = "black", pch = 21) + scale_fill_manual(values = c("#A93154",
    "#BA4B8E", "#BC6EB9", "#B494D5", "#AEB6E5")) + scale_color_manual(values = c("#A93154",
    "#BA4B8E", "#BC6EB9", "#B494D5", "#AEB6E5")) + theme_bw() +
    labs(title = "Monognathic heterodonty", x = "Standardized Positions",
        y = "PC") + theme(axis.title = element_text(color = "#666666",
    face = "bold", size = 8)) + theme(axis.text.x = element_text(angle = 45,
    vjust = 0.5), axis.text.y = element_text(size = 8), axis.text.y.left = element_text(size = 8),
    axis.ticks.x = element_blank(), legend.position = "none") +
    facet_wrap(~variable, ncol = 2, scales = "free_y")
```

```
# Dignathic morphospace for all sharks.  Box plots:
# PC1-PC4.
ggplot(na.omit(me1_melt), aes(x = unit, y = value, fill = unit,
    colour = unit)) + geom_point(position = position_jitterdodge(dodge.width = 0.9),
    alpha = 0.5, size = 0.2) + geom_boxplot(outlier.size = 3,
    alpha = 0.2, fill = "white") + stat_summary(fun.data = mean_cl_boot,
    geom = "errorbar", width = 0.1, lwd = 0.25, linetype = 1,
    col = "black") + stat_summary(fun = "mean", geom = "point",
    lwd = 2, position = position_dodge(width = 1), bg = "white",
    color = "black", pch = 21) + scale_fill_manual(values = c("#006400",
    "#8B2252")) + scale_color_manual(values = c("#006400", "#8B2252")) +
    theme_bw() + labs(title = "Dignathic heterodonty", x = "Dental Units",
    y = "PC") + theme(axis.title = element_text(color = "#666666",
    face = "bold", size = 10)) + theme(axis.text.x = element_text(angle = 45),
    axis.text.y = element_text(face = "bold", size = 10), axis.text.y.left = element_text(size = 8),
    axis.ticks.x = element_blank(), legend.position = "none") +
    facet_wrap(~variable, ncol = 2, scales = "free_y")
```

Statistical analyses of heterdonty

Does "standardized" shark (i.e., Selachimorpha) tooth positions and dental units occupy different areas of morphospace?

```
stat.frame <- geomorph.data.frame(scores = scores[rownames(monoGnathic.Df),
    ], position = factor(monoGnathic.Df$Standardized.Position),
    unit = factor(monoGnathic.Df$Dental.Unit), order = factor(monoGnathic.Df$Order))

unit.frame <- geomorph.data.frame(scores = scores[rownames(dentalUnit.Df),
    ], unit = factor(dentalUnit.Df$Dental.Unit), order = factor(dentalUnit.Df$Order))
# Fit models.
pos.aov <- lm.rrpp(f1 = scores ~ position, iter = 999, RRPP = TRUE,
    SS.type = "I", data = stat.frame)
uni.aov <- lm.rrpp(f1 = scores ~ unit, iter = 999, RRPP = TRUE,
    SS.type = "I", data = unit.frame)

anova(pos.aov)
anova(uni.aov)
```

Monognathic heterodonty over time

```
# Re-level
me1_melt$time <- factor(me1_melt$time, levels = geo.ages)
```

```
# Global four-stage time binning scheme: tooth positions
ggplot(na.omit(me1_melt), aes(x = position, y = value, fill = position,
    colour = position)) + geom_boxplot(outlier.colour = "grey",
    varwidth = F, outlier.size = 3, alpha = 0.2, fill = "white") +
    geom_point(position = position_jitterdodge(dodge.width = 0.9),
        alpha = 0.5, size = 0.2) + stat_summary(fun.data = mean_cl_boot,
    geom = "errorbar", width = 0.25, lwd = 0.5, linetype = 1,
    col = "black") + stat_summary(fun = "mean", geom = "point",
    lwd = 1, position = position_dodge(width = 0.25), bg = "white",
    color = "black", pch = 21) + scale_fill_manual(values = c("#A93154",
    "#BA4B8E", "#B494D5", "#AEB6E5")) + scale_color_manual(values = c("#A93154",
    "#BA4B8E", "#B494D5", "#AEB6E5")) + facet_grid(variable ~
    time, scales = "free_y", margins = FALSE) + labs(x = "Time (Ma)",
    y = "PC") + theme_classic() + theme(legend.position = "right") +
    theme(axis.title = element_text(color = "#666666", face = "bold",
        size = 12)) + theme(axis.title.x = element_blank(), axis.text.x = element_blank(),
    axis.ticks.x = element_blank(), legend.position = "none")
```

Dignathic heterodonty over time

```
# Global four-stage time binning scheme: dental units.
ggplot(na.omit(me1_melt), aes(x = unit, y = value, fill = unit,
    colour = unit)) + geom_boxplot(outlier.colour = "grey", varwidth = F,
    outlier.size = 3, alpha = 0.2, fill = "white") + geom_point(position = position_jitterdodge(dodge.wi
    alpha = 0.5, size = 0.2) + stat_summary(fun.data = mean_cl_boot,
    geom = "errorbar", width = 0.25, lwd = 0.5, linetype = 1,
    col = "black") + stat_summary(fun = "mean", geom = "point",
    lwd = 1, position = position_dodge(width = 0.25), bg = "white",
    color = "black", pch = 21) + scale_fill_manual(values = c("#006400",
    "#8B2252")) + scale_color_manual(values = c("#006400", "#8B2252")) +
    facet_grid(variable ~ time, scales = "free", margins = FALSE) +
    labs(x = "Time (Ma)", y = "PC") + theme_classic() + theme(legend.position = "right") +
    theme(axis.title = element_text(color = "#666666", face = "bold",
        size = 12)) + theme(axis.title.x = element_blank(), axis.text.x = element_blank(),
    axis.ticks.x = element_blank(), legend.position = "none")
```

Monognathic and dignathic heterodonty by clade

Hexanchidae have well pronuced heterodonty of different sorts. Heterodontidae have a disjunct monognathic heterodont dentition that changes through ontogeny.

```
# PC1-PC4.
ggplot(me1_melt, aes(x = position, y = value, fill = order, colour = order)) +
    geom_point(position = position_jitterdodge(dodge.width = 0.9),
        alpha = 0.5, size = 0.2) + geom_boxplot(outlier.size = 3,
    alpha = 0.2, fill = "white") + scale_fill_manual(values = alpha(c("#E84646",
    "#377EB8", "darkorange", "purple4", "black", "green4", "goldenrod1",
    "violetred4", "darkcyan"), 0.45)) + scale_color_manual(values = alpha(c("#E84646",
    "#377EB8", "darkorange", "purple4", "black", "green4", "goldenrod1",
    "violetred4", "darkcyan"), 0.45)) + stat_summary(fun.data = mean_cl_boot,
    geom = "errorbar", width = 0.15, lwd = 0.3, linetype = 1,
    col = "black") + stat_summary(fun = "mean", geom = "point",
    lwd = 0.5, position = position_dodge(width = 1), bg = "white",
    color = "black", pch = 21) + facet_grid(variable ~ order,
```

```
    scales = "free", shrink = T) + theme_bw() + xlab("Standardized Positions") +
    ylab("PC") + theme(axis.text.x = element_text(angle = 45,
    hjust = 1), axis.title.x = element_text(face = "bold", size = 10),
    axis.title.y.left = element_text(face = "bold"), legend.position = "none",
    strip.text.x = element_text(size = 8, face = "bold", angle = 45),
    axis.text = element_text(colour = "#4D4D4D", size = 8))
```

```
# PC1-PC4.
ggplot(na.omit(me1_melt), aes(x = unit, y = value, fill = order,
    colour = order)) + geom_point(position = position_jitterdodge(dodge.width = 0.9),
    alpha = 0.5, size = 0.2) + geom_boxplot(outlier.size = 3,
    alpha = 0.2, fill = "white") + scale_fill_manual(values = alpha(c("#E84646",
    "#377EB8", "black", "green4", "violetred4", "darkcyan"),
    0.45)) + scale_color_manual(values = alpha(c("#E84646", "#377EB8",
    "black", "green4", "violetred4", "darkcyan"), 0.45)) + stat_summary(fun.data = mean_cl_boot,
    geom = "errorbar", width = 0.15, lwd = 0.3, linetype = 1,
    col = "black") + stat_summary(fun = "mean", geom = "point",
    lwd = 0.5, position = position_dodge(width = 1), bg = "white",
    color = "black", pch = 21) + facet_grid(variable ~ order,
    scales = "free", shrink = T) + theme_bw() + xlab("Dental Units") +
    ylab("PC") + theme(axis.text.x = element_text(angle = 45,
    hjust = 1), axis.title.x = element_text(face = "bold", size = 10),
    axis.title.y.left = element_text(face = "bold"), legend.position = "none",
    strip.text.x = element_text(size = 8, face = "bold", angle = 45),
    axis.text = element_text(colour = "#4D4D4D", size = 8))
```

```

# Fit monognathic model.
aov.pos <- lm.rrpp(f1 = scores ~ position + order, iter = 999,
    RRPP = TRUE, SS.type = "I", print.progress = TRUE, data = stat.frame)
anova(aov.pos)
# Pairwise comparisons.
pos.PW <- pairwise(fit = aov.pos, groups = interaction(stat.frame$position))
summary(pos.PW, test.type = "dist", confidence = 0.95, stat.table = TRUE)
# Fit dignathic model.
aov.unit <- lm.rrpp(f1 = scores ~ unit + order, iter = 999, RRPP = TRUE,
    SS.type = "I", print.progress = TRUE, data = unit.frame)
anova(aov.unit)
# Pairwise comparisons.
unit.PW <- pairwise(fit = aov.unit, groups = interaction(unit.frame$unit))
summary(unit.PW, test.type = "dist", confidence = 0.95, stat.table = TRUE)
```

Statistical evalutation of heterodonty by clade

Ordinary Least Squares regression (OLS)

Because some tooth images depicted only lingual or unspecified views, we used ordinary least-squares linear models to test for discrepancies in landmark configurations using a sub-sample with combined labial and lingual views. Sample size considered: **866 specimens.**

```
# Load data frame.
OLS.Df <- read.xlsx(file = "Morphometric & Other Excel Datasets/OLS Regression Lingual vs. Labial Dataf
    sheetIndex = 1)
rownames(OLS.Df) <- paste(OLS.Df$File.Name, OLS.Df$File.Type,
    sep = "")
```

```r
# Subset landmark coordinates to match rownames of data
# frame.
LM.points <- LMs$coords[, , rownames(OLS.Df)]
# GPA.
viewGPA <- gpagen(A = LM.points, curves = as.matrix(LMs$sliders[-80,
    ]), ProcD = FALSE)
```

```
##
## Performing GPA
##   |                                                           |
##
## Making projections... Finished!
```

```r
# PCA.
pca <- gm.prcomp(viewGPA$coords)

# Create variables for OLS analysis.  Labial teeth.
pc1.labial <- pca$x[OLS.Df$View == "labial", 1]
pc2.labial <- pca$x[OLS.Df$View == "labial", 2]
# Lingual teeth.
pc1.lingual <- pca$x[OLS.Df$View == "lingual", 1]
pc2.lingual <- pca$x[OLS.Df$View == "lingual", 2]

# Data frame.
df.views <- data.frame(scores = pca$x, views = OLS.Df$View)
pc.1.df.views <- data.frame(x = pc1.labial, y = pc1.lingual)
pc.2.df.views <- data.frame(x = pc2.labial, y = pc2.lingual)

# Linear model fit.
sma(pc1.labial ~ pc1.lingual, slope.test = 1, elev.test = 0,
    alpha = 0.05, quiet = TRUE)
```

```
## Call: sma(formula = pc1.labial ~ pc1.lingual, alpha = 0.05, slope.test = 1,
##     elev.test = 0, quiet = TRUE)
##
## Fit using Standardized Major Axis
##
## ------------------------------------------------------------
## Coefficients:
##                elevation     slope
## estimate    -0.008037348 0.9883856
## lower limit -0.012568457 0.9697234
## upper limit -0.003506239 1.0074070
##
## H0 : variables uncorrelated
## R-squared : 0.9594556
## P-value : < 2.22e-16
##
## ------------------------------------------------------------
## H0 : slope not different from 1
## Test statistic : r= -0.05792 with 431 degrees of freedom under H0
## P-value : 0.22905
## ------------------------------------------------------------
## H0 : elevation not different from 0
## Test statistic: t= -3.486 with 431 degrees of freedom under H0
```

```
## P-value : 0.0005398
```

```r
sma(pc2.labial ~ pc2.lingual, slope.test = 1, elev.test = 0,
    alpha = 0.05, quiet = TRUE)
```

```
## Call: sma(formula = pc2.labial ~ pc2.lingual, alpha = 0.05, slope.test = 1,
##     elev.test = 0, quiet = TRUE)
##
## Fit using Standardized Major Axis
##
## ------------------------------------------------------------
## Coefficients:
##                elevation     slope
## estimate     0.001084086 1.0184437
## lower limit -0.001984165 0.9906333
## upper limit  0.004152337 1.0470349
##
## H0 : variables uncorrelated
## R-squared : 0.9144564
## P-value : < 2.22e-16
##
## ------------------------------------------------------------
## H0 : slope not different from 1
## Test statistic : r= 0.06237 with 431 degrees of freedom under H0
## P-value : 0.19522
## ------------------------------------------------------------
## H0 : elevation not different from 0
## Test statistic: t= 0.6945 with 431 degrees of freedom under H0
## P-value : 0.48777
```

```r
# slope.test = 1; elev.test = 0
ov <- lm(pc1.labial ~ pc1.lingual, pc.1.df.views)
marginalModelPlots(ov)
```

```r
# Shape-space
a <- ggplot(df.views, aes(x = scores.Comp1, y = scores.Comp2,
    color = views)) + geom_point(pch = 19, alpha = 0.5) + scale_color_manual(values = c("#338080E6",
    "#CC3380E6")) + scale_fill_manual(values = c("#338080E6",
    "#CC3380E6")) + theme_bw() + xlab("Principal Component 1") +
    ylab("Principal Component 2") + ggtitle("Morphospace of shark teeth",
    subtitle = "Labial vs. Lingual Views (N = 866)") + theme(axis.title.x = element_text(face = "bold")
    axis.title.y.left = element_text(face = "bold")) + geom_hline(yintercept = 0,
    linetype = 1) + geom_vline(xintercept = 0, linetype = 1) +
    theme(legend.justification = c(0, 1), legend.position = c(0,
        1), axis.title.x = element_text(size = 10), axis.title.y = element_text(size = 10),
        axis.title = element_text(size = 12)) + geom_density2d()

# Regression plots.
b <- ggplot(pc.1.df.views, aes(x = x, y = y)) + geom_point(pch = 19,
    alpha = 0.5) + xlab("PC1 (Labial)") + ylab("PC1 (Lingual)") +
    theme_classic() + theme(legend.position = "none") + geom_smooth(method = lm,
    se = F, color = "black") + theme(axis.title.x = element_text(face = "bold",
    size = 10), axis.title.y.left = element_text(face = "bold"),
    axis.title.y = element_text(size = 10))
```

```r
c <- ggplot(pc.2.df.views, aes(x = x, y = y)) + geom_point(pch = 19,
    alpha = 0.5) + xlab("PC2 (Labial)") + ylab("PC2 (Lingual)") +
    theme_classic() + geom_smooth(method = lm, se = F, color = "black") +
    theme(legend.position = "none") + theme(axis.title.x = element_text(face = "bold",
    size = 10), axis.title.y.left = element_text(face = "bold"),
    axis.title.y = element_text(size = 10))
# Arrange plots.
ggarrange(a, ggarrange(b, c, labels = c("B", "C"), nrow = 2),
    ncol = 2, labels = "A", widths = 4:3)
```

```
## `geom_smooth()` using formula 'y ~ x'
## `geom_smooth()` using formula 'y ~ x'
```

OLS-regression by order

```r
# Drop Echinorhiniformes from the data frame.
new.OLS.Df <- OLS.Df[OLS.Df$Order != "Echinorhiniformes", ]
# Re-order levels
new.OLS.Df$Re.Order <- factor(new.OLS.Df$Order, levels = orders)
# Subset pc-scores.
new.scores <- pca$x[rownames(new.OLS.Df), ]
pc1LAB <- new.scores[new.OLS.Df$View == "labial", 1]
pc1LIN <- new.scores[new.OLS.Df$View == "lingual", 1]
# Data frame.
order.view <- data.frame(data = new.scores, x = pc1LAB, y = pc1LIN,
    order = new.OLS.Df$Re.Order)

# Facet OLS regression plot.
ggplot(order.view, aes(x = x, y = y)) + geom_point(pch = 19,
    alpha = 0.5) + xlab("Principal Component 2 (Labial)") + ylab("Principal Component 2 (Lingual)") +
    theme_bw() + theme(legend.position = "none") + geom_smooth(method = lm,
    se = F, color = "black") + theme(axis.title.x = element_text(face = "bold",
    size = 10), axis.title.y.left = element_text(face = "bold"),
    legend.position = "top") + facet_wrap(~order, ncol = 2) +
    theme(strip.text.x = element_text(size = 8, face = "bold"))
```

```
## `geom_smooth()` using formula 'y ~ x'
```

The End.
